# Pyrrolopyrimidine derivatives as dual COX-2/ACE2 inhibitors: design, synthesis, and anti-inflammatory evaluation

**DOI:** 10.3389/fmolb.2025.1710650

**Published:** 2026-01-06

**Authors:** Hala Afifi, Samar S. Fatahala, Rania H. Abd El-Hameed, Shahenda Mahgoub, Radwan El-Haggar, Omnia Aly, Amal F. Gharib, Amira I. Sayed, Heba Taha

**Affiliations:** 1 Research Directorate, City University Ajman, Ajman, United Arab Emirates; 2 Department of Pharmaceutical Chemistry, Faculty of Pharmacy, Ain-Shams University, Cairo, Egypt; 3 Pharmaceutical Organic Chemistry Department, Faculty of Pharmacy, Helwan University, Cairo, Egypt; 4 Department of Biochemistry and Molecular Biology, Faculty of Pharmacy, Helwan University, Cairo, Egypt; 5 Department of Pharmaceutical Chemistry, Faculty of Pharmacy, Helwan University, Cairo, Egypt; 6 Medical Biochemistry Department, National Research Centre, Dokki, Egypt; 7 Department of Clinical Laboratory Sciences, College of Applied Medical Sciences, Taif University, Taif, Saudi Arabia; 8 Department of Pharmaceutical Chemistry, Faculty of Pharmacy, ACU University, Cairo, Egypt

**Keywords:** pyrrolopyrimidines, molecular docking/simulation, cytokines, SARS-CoV-2, COX-2 inhibition, ACE2 inhibition

## Abstract

In this study, we report the design and synthesis of a new series of pyrrolopyrimidine derivatives developed as dual-target nonsteroidal anti-inflammatory agents (NSAIDs). The compounds were evaluated for anti-inflammatory properties, cyclooxygenase-1/2 (COX-1/COX-2) inhibitory activity, and angiotensin-converting enzyme 2 (ACE2)–blocking activity in lipopolysaccharide (lipopolysaccharide)-stimulated RAW264.7 cells. Among the synthesized molecules, compounds **5a** and **5b** showed potent dual inhibitory activity, which was supported by molecular docking and molecular dynamics simulations. These findings highlight the potential of selective COX-2 inhibitors with concurrent ACE2 blockade as a promising therapeutic approach for controlling inflammation and modulating pathways relevant to viral entry and other inflammation-associated disorders. While ACE2 inhibition has received particular attention in the context of recent viral infections, the broader anti-inflammatory efficacy of these derivatives supports their potential as multi-target drug candidates.

## Graphical Abstract

**Figure:**
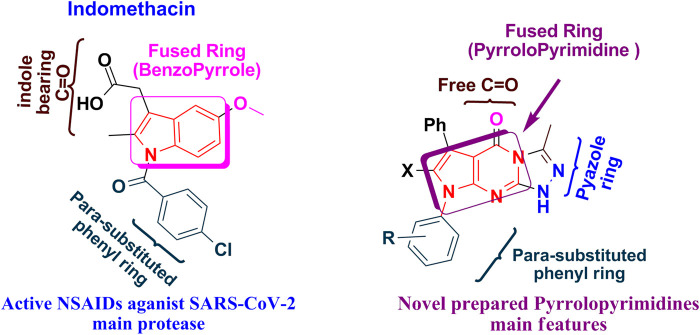

## 1 Introduction

Chronic inflammation is a key contributor to multiple debilitating diseases, including lung injury and other inflammation-associated disorders (Yang et al., 2021; Jose et al., 2022; Forcados et al., 2021; Saeedi-Boroujeni and Mahmoudian-Sani, 2021). Recent global viral outbreaks, including those caused by coronaviruses, pose significant public health risks (Dong et al., 2022). Highly transmissible variants have resulted in several major infection waves worldwide, starting with Omicron (B.1.1.529) in early 2020, which became the predominant variant after the summer of 2021. Additional Omicron sub-lineages (BA.2, BA.3, BA.4, and BA.5) appeared in 2022 (US Centers for Disease Control and Prevention, 2025; Johns Hopkins Coronavirus Resource Center, 2025).

Inflammatory responses are initiated by macrophages, which play critical roles by secreting numerous pro-inflammatory mediators and cytokines, including C-reactive protein (CRP), interleukin-1β (IL-1β), IL-6, and cyclooxygenase-2 (COX-2) (Rashid et al., 2021; Saleh et al., 2021; Masih et al., 2021; Menche, 2021). Early in the inflammatory response, damage-associated molecular patterns (DAMPs) are recognized. Moreover, TLR4-mediated inflammation (Sayed et al., 2022), triggered by DAMPs, is implicated in several pathologies, including sepsis, a potential complication of SARS-CoV-2 infection (Manjili et al., 2020).

**FIGURE 1:**
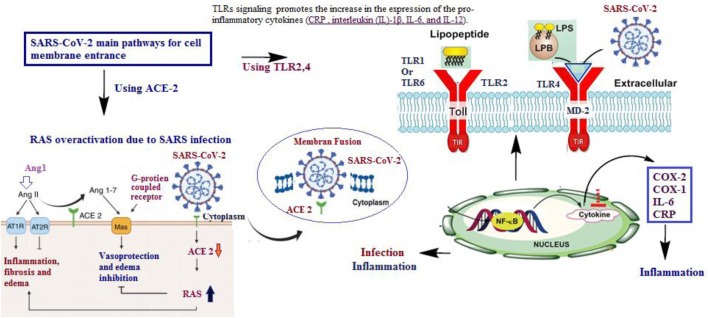
The two main pathways for SARS-CoV-2 effects on the cell are either through infection or induced inflammation (Rashid et al., 2021; Hamet et al., 2021; Fazio and Bellavite, 2023). Toll-like receptors (TLRs) are necessary for the recognition of diacetylated and triacetylated lipopeptides, which are components of microbial cell walls. This recognition is based on the nature of the endotoxin lipopolysaccharide (LPS) and is achieved through accessory molecules like LPS-binding protein (LBP) (Lee et al., 2012; Molinaro et al., 2015).

The inflammatory response in viral, inflammation-associated disorders begins when the spike protein of coronaviruses uses host cell ACE2 to enter target cells (Hoffmann et al., 2020). Following entry, viral replication and lysis of infected cells induce IFN-γ and the release of inflammatory cytokines (e.g., TNF-α and IL-6), as well as free radicals, leading to recruitment and activation of leukocyte subsets that further release cytokines and other mediators (Perico et al., 2021). This response can be sustained by increased angiotensin II resulting from ACE2 downregulation due to continuous recycling of the receptor during viral entry (Vaduganathan et al., 2020). Angiotensin II also promotes inflammation by inducing mononuclear-cell proliferation and recruitment of pro-inflammatory cells (Vaduganathan et al., 2020; Ruiz-Ortega et al., 2001). Because mild to moderate symptoms may reflect intense underlying inflammation, there is interest in early treatment of outpatients with viral, inflammation-associated disorders. Early intervention may help prevent progression to severe disease and reduce long-term complications. Thus, anti-inflammatory drugs in the early stage may be beneficial (Hamet et al., 2021). Inhibitors that block ACE2 binding to the SARS-CoV-2 spike RBD may also offer protection against infection and its inflammatory sequelae (Beyerstedt et al., 2021; Shirbhate et al., 2021; González-rayas et al., 2020; Chatterjee and Thakur, 2020; Teral et al., 2020) (Figure 1).

Novel methods for examining the relationship between TLRs and COX-2 were used to study several inflammatory pathways (Wang et al., 2017; Lin et al., 2016; Marshall et al., 2016; Elisha et al., 2016; Makene and Pool, 2015). The primary focus was on the signs of response to acute inflammatory stimulation, such as leukotrienes and prostaglandins (PG), which were released in response to SARS-CoV-2 and other agents. Blocking PGE production is thought to be a crucial anti-inflammatory tactic (Lin et al., 2016; Chen et al., 2018; Zhao et al., 2022). Two distinct cyclooxygenases (COXs), COX-1 (constitutive) and COX-2 (inducible), use Acetylacetone (AA) to produce PGE2 (Jannus et al., 2021; Abd El-Hameed et al., 2021; Ren et al., 2020; Nie et al., 2019; Shen et al., 2019; Hwang et al., 2019; Wang and Wang, 2018; Flefel et al., 2018; Chen et al., 2017; Lv et al., 2017). Prostaglandin production throughout various inflammatory diseases is attributed to COX-2 (Chen et al., 2018; Zhao et al., 2022; Banerjee et al., 2015). By selectively inhibiting COX-2 or non-selectively inhibiting both COX enzymes, NSAIDs reduce the formation of PGE2. Selective COX-2 inhibitors aim to minimize gastrointestinal and renal adverse effects by preserving gastroprotective prostaglandins produced by COX-1 activity (Khalil et al., 2024).

**FIGURE 2:**
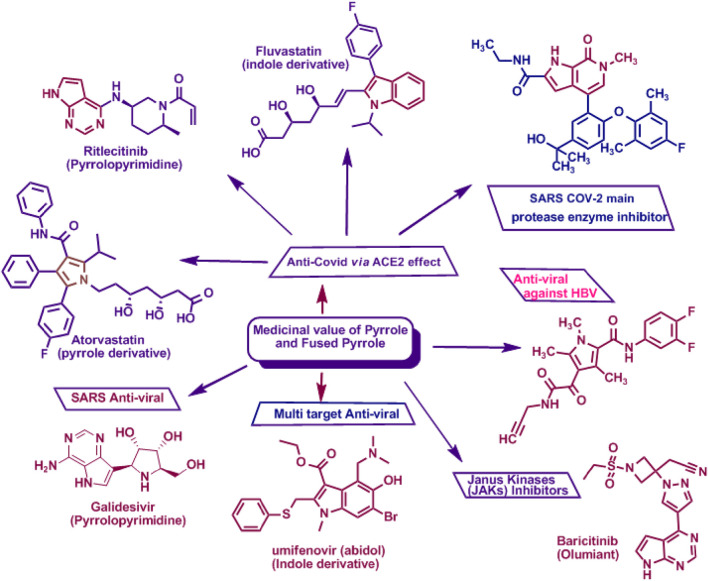
Pyrrole and fused pyrroles as antivirals, including anti-COVID, via affecting ACE2 and multiple antiviral targets (Sayed et al., 2022; Fatahala et al., 2022; Negi et al., 2020).

Due to their biological significance (Kassab, 2025; Tylińska et al., 2024; Wang et al., 2025; Abdollahi et al., 2025; Sahu et al., 2026; Teno and Masuya, 2010; Gries et al., 2025) and potential uses as antiviral agents, particularly against COVID-19 (Guillon et al., 2025; Pfannenstiel et al., 2025; Lei et al., 2022); both pyrrole and pyrrolopyrimidine derivatives have attracted increasing attention for several decades (Bonelli et al., 2023; Kinoshita-Ise et al., 2023). Blocking the viral life cycle and thus preventing the consequent inflammatory responses via targeting ACE2 (Wang et al., 2025) has been the main aim of most recent therapeutics. Among these, the pyrrole derivative, atorvastatin (Lipitor®), which regulates the global expression of ACE2 in cells, prevents the correct interaction of the viral spike protein with its receptor (Al-Horani et al., 2020; Yang et al., 2020) and reduces the expression of ACE2. Ritlecitinib (a pyrrolopyrimidine derivative) is an irreversible inhibitor of JAK3, which is closely correlated with ACE2 and the JAK–STAT pathway in the regulation of immune response signaling to modulate SARS-CoV-2 (Jain et al., 2023). Another pyrrolopyrimidine, galidesivir, is an adenosine analog and RNA polymerase inhibitor, with potential broad-spectrum antiviral activity (Julander et al., 2021). Umifenovir (Arbidol), an antiviral drug with multiple antiviral properties, acts by binding to the SARS-CoV-2 spike S protein (Al-Horani et al., 2020). Baricitinib, another pyrrolopyrimidine, affects SARS-CoV-2 binding to the spike S protein (Al-Horani et al., 2020), as revealed in Figure 2.

**FIGURE 3:**
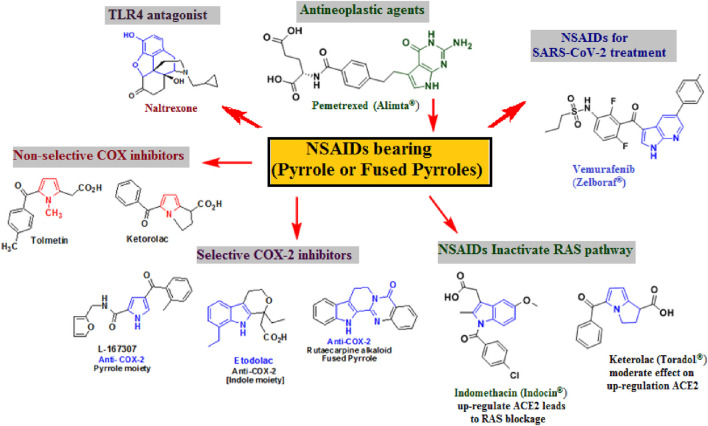
Pyrrole and fused pyrrole as NSAIDs (Manjili et al., 2020; Chatterjee and Thakur, 2020; Negi et al., 2020; Valenzuela et al., 2021; Oh et al., 2021; Battilocchio et al., 2013).

Owing to the great importance of the pyrrole nucleus as a pharmacophore in many drugs (Rashad et al., 2024; Sai Madhurya et al., 2024), scientists were able to find more pyrrole and fused pyrrole derivatives (Rashid et al., 2021; Negi et al., 2020) with amazing biological characteristics (Pathania and Rawal, 2018). Among anti-inflammatory agents (Tylińska et al., 2024; Wang et al., 2025; Teno and Masuya, 2010) are the widely recognized NSAIDs bearing pyrrole or its fused moieties (Mateev et al., 2022; Bindu et al., 2020; Jeelan Basha et al., 2022), as revealed in Figure 3.

The use of NSAIDs to treat COVID-19 has been linked to multiple signaling pathways (Valenzuela et al., 2021; Oh et al., 2021; Battilocchio et al., 2013; La Monica et al., 2022; Küper et al., 2012). One of these pathways is the renin–angiotensin system (RAS) signaling pathway via suppression of the angiotensin I-converting enzyme (ACE), which may help COVID-19 patients experience less tissue damage (Valenzuela et al., 2021; Oh et al., 2021). SARS-CoV-2 uses the ACE2 receptor to infect lung alveolar epithelial cells (Sayed et al., 2022; Beyerstedt et al., 2021; Shirbhate et al., 2021; González-rayas et al., 2020). As a result of the viral internalization, ACE2 is downregulated on the host cell surface, which is linked to a decreased angiotensin 1–7 (Ang 1–7) and increased Ang II, respectively (Beyerstedt et al., 2021; González-rayas et al., 2020; Valenzuela et al., 2021). Angiotensin-related damage to the heart and lungs could result from this imbalance.

Consequently, by minimizing the negative effects connected to Ang II, RAS blocking may help to restore the RAS balance. RAS inhibitors have emerged as a potentially effective approach for treating COVID-19-related acute-severe pneumonia (Oh et al., 2021; Küper et al., 2012). Indomethacin, fused pyrrole analogs, showed the highest ability to control the cytokine storm by inhibiting lung inflammation through the deactivation of the RAS signaling system. The current interest is the non-selective COX inhibitor, indomethacin, due to the rapid recovery of many COVID-19 patients from cough and pain, as well as the control of D-dimer levels (Fazio and Bellavite, 2023; Elmaaty et al., 2021). On the other hand, when ibuprofen or hydroxychloroquine were used, there were no discernible gains to health (Manjili et al., 2020; Oh et al., 2021). Thus, indomethacin appears to offer the best structural features among the NSAIDs for suppressing the pathophysiological pathways that the virus uses to intensify the illness (Fazio and Bellavite, 2023; Elmaaty et al., 2021). Indomethacin has several modes of action that are molecule-specific and make the drug more relevant in COVID-19 (Fazio and Bellavite, 2023; Elmaaty et al., 2021; Prasher et al., 2021). First, the conventional approach involves non-selective inhibition of COX-1 and COX-2, which permits the regulation of pro-inflammatory cytokine and chemokine overproduction and overexpression (La Monica et al., 2022; Chen et al., 2021). Second, its capacity to inhibit the BCL2-associated agonist of cell death, which is connected to the production of cytokines (IL-6 and IL-8), suggested that it can control pathways that lead to inflammation (Fazio and Bellavite, 2023; Consolaro et al., 2022; McCarthy et al., 2021). Third, an excess of Ang II and a deficiency of ACE2 in COVID-19 leads to an imbalance in the regulation of the RAS, which raises bradykinin levels and causes a “bradykinin storm” of bronchoconstriction and vasodilation. Thus, indomethacin is among the best NSAIDs to prevent the consequences of this imbalance (Consolaro et al., 2022; McCarthy et al., 2021; Gomeni et al., 2020; Haybar et al., 2021).

Despite these important benefits, pharmacological interactions between widely used NSAIDS and cardiovascular therapies have recently come under scrutiny in several clinical contexts (Lattuca et al., 2013; Ushiyama et al., 2008). Currently, due to the disruption of the balance between COX-1-derived prothrombotic thromboxane A_2_ and COX-2-derived antithrombotic prostacyclin, COX-2 inhibitors are recommended for symptomatic management at the lowest effective dose in patients with elevated cardiovascular risk. New compounds are added every day to combat the side effects of NSAIDS and to find new, safe anti-inflammatory drugs that target TLRs and/or ACE2 as new, potent anti-inflammatories bearing anti-SARS-CoV-2 activities (Bocheva et al., 2006; Lessigiarska et al., 2005; Khalil et al., 2024).

## 2 Results and discussion

### 2.1 Chemistry

**FIGURE 4:**
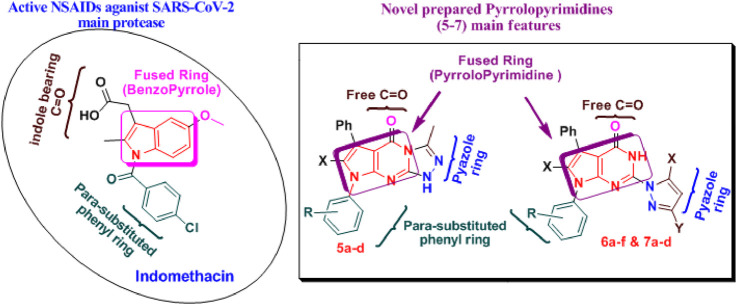
Connection point between active NSAIDs (indomethacin) and the prepared compounds **5–7** (Fazio and Bellavite, 2023; Elmaaty et al., 2021). This figure illustrates the principal structure-activity relationships (SARs) of the NSAIDs, indomethacin, and compounds containing fused pyrrole, which were determined by examining their stability and binding scores to the SARS-CoV-2 main protease. The following outcomes are the result of these studies: 1) it was found that the optimal activity was achieved by keeping fused indoles or pyrroles bearing an oxygen atom, such as -OH or –C=O; 2) it was preferred to add the electron-donating group (EDG) to the para position of the phenyl ring (as OH/OR or EDG as Me); 3) the addition of the halo group to the phenyl ring attached to the primary scaffold increased the activity; 4) the substitution of a pyrazolidine ring increased activity (Fazio and Bellavite, 2023; Elmaaty et al., 2021).

**SCHEME 1:**
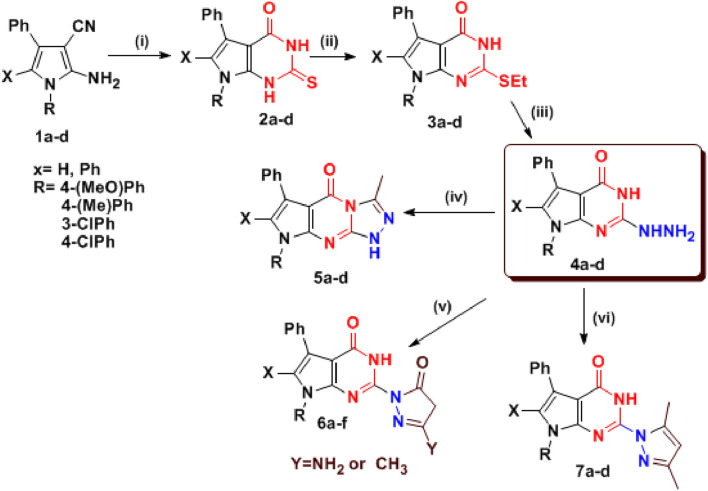
Synthesis of pyrrolopyrimidines and pyrrolotriazolopyrimidines (**2–7**).

The remarkable biological activity of pyrrolopyrimidines and fused pyrrolopyrimidine derivatives (Flefel et al., 2018; Zaki et al., 2016; Hossain and Bhuiyan, 2009; Mohamed et al., 2013) has inspired us to synthesize new derivatives and test their anti-inflammatory activity (Fatahala et al., 2017a; Fatahala et al., 2017b). This article highlights some novel synthetic pathways of pyrrole and its fused compounds, especially pyrrolopyrimidine, as a continuation of our previous work on the preparation of novel anti-inflammatory compounds (Mohame et al., 2012; Mohamed et al., 2010a; Mohamed et al., 2011; Mohamed et al., 2010b). Some novel fused pyrrolopyrimidines **3–7**, structurally resembling indomethacin, namely, pyrrolotriazolopyrimidines and cyclic hydrazones derivatives, were synthesized, docked, and screened for their ACE2 inhibition and anti-inflammatory activities via COX enzyme inhibition. Additionally, molecular dynamic simulations (MDS) were conducted for 100 ns using GROMACS 2.1.1 software using the docking coordinates of COX-2 bound to the most promising compounds **5a** and **5b**. The MD simulation was performed to provide insights into the precise estimation of the binding strength of a docked complex of COX-2, bound to compounds **5a** and **5b**, as revealed in Figure 4. The synthetic strategies for our target compounds are presented in Scheme 1.

To continue our previously reported methods for preparation of bioactive pyrroles and fused pyrroles (Sayed et al., 2022; Mohamed et al., 2013; Fatahala et al., 2017b; Mohamed et al., 2009; Mohamed et al., 2013; El-Hameed et al., 2021; Fatahala et al., 2017c; Fatahala et al., 2018), we previously reported that 2-aminopyrrole-3-carbonitriles **1(a–d)** (Mohamed et al., 2015; Mohamed et al., 2019) were heated under reflux, independently, with thiourea in absolute ethanol in order to prepare pyrrrolopyrimidine-2-thione-4-one derivatives **2(a–d)**. These derivatives were analyzed using different spectral analysis techniques to confirm their structures. An examination of the main criteria in IR spectra of these compounds revealed that the CN absorption bands, characterized for derivatives **1(a–f)**, disappeared in the **2(a–d)** spectra, and other main absorption bands for C=O and C=S appeared. Upon alkylation of **2(a–f)** with ethyl iodide/Na_2_CO_3_, thio-ethyl derivatives **3(a–d)** were afforded and confirmed by all spectral data, especially the appearance of aliphatic proton signals in ^1^H-NMR spectra. Upon reacting **3(a–d)** with hydrazine hydrate in absolute ethanol, the 2-hydrazino-pyrrolopyrimidines **4(a–d)** were afforded and confirmed by spectral data, mainly the appearance of NH and NH_2_ signals in ^1^H-NMR and their characterized absorption bands in IR spectra (See Supplementary Material for full details and a derivatives table.).

2-Hydrazino-pyrrolopyrimidines **4(a–d)** were subsequently used as starting materials for the synthesis of other novel derivatives (Mohamed et al., 2019; Ghoshal et al., 2020). In brief, **pyrrolotriazolopyrimidin-5-one (5a–d)** were obtained via the reaction of hydrazino derivatives **4(a–d)** with acetic anhydride (Hossain and Bhuiyan, 2009; Abdel-Mohsen, 2005). In addition, 2-hydrazino derivatives **4(a–d)** reacted with active methylene compounds (namely, ethyl cyanoacetate [ECA] or ethyl acetoacetate [EAA]) in a strongly basic medium (NaOEt), giving the 2-pyrazolonyl derivatives **6 (a–f)**. Finally, reaction of 2-hydrazino derivatives **4** with acetylacetone [AA] in absolute ethanol using catalytic amounts of glacial acetic acid resulted in the formation of the 2-pyrazolyl-pyrrolopyrimidines **7(a–d)**. All of these derivatives were supported with elemental analysis and spectral data (Flefel et al., 2018; Zaki et al., 2016). Many features were confirmed in spectral data in the form of the disappearance of NH_2_ group absorption bands in the IR spectra, as well as its signal in ^1^H-NMR spectra, also that of the NH group in some of these compounds, and the increasing number of aromatic protons and/or aliphatic protons (See Supplementary Material for full details and the derivatives table.).

#### 2.1.1 Biological evaluation

COX-2 is a key enzyme in numerous physiological and pathological events (Roskosky et al., 2022). It plays a fundamental role in viral infections and controls the expression of abundant serum proteins (Liu et al., 2011). Tissue samples obtained from dead patients of avian influenza H5N1 infection reported overstimulation of COX-2 in the autoptic lung epithelial cells, along with elevated levels of TNF-α and several pro-inflammatory cytokines (Baghaki et al., 2020). In contrast, knocking out the gene encoding for COX-2 in an H3N2 influenza A model in mice resulted in less severe infection via the decreased inflammatory response and cytokine release and better survival than the wild-type mice (Carey et al., 2005). It was also reported that SARS-CoV-2 infection induced the expression of COX-2, which may suggest that COX-2 cascade signaling may be a significant pathway for the regulation of SARS-CoV-2 infection or the virus-induced immune response (Chen et al., 2021).

Likewise, COX-1, COX-2, and prostaglandin E synthase (PTGES) were upregulated in peripheral blood mononuclear cells isolated from COVID-19 patients (Yan et al., 2021). A clinical study showed that administration of celecoxib (a selective COX-2 inhibitor) as adjuvant treatment for 7–14 days to patients with moderate COVID-19 symptoms prevented clinical worsening and improved chest CT scoring compared to the standard therapy (Hong et al., 2020). Moreover, treatment of non-hospitalized patients with early mild or moderate COVID-19 symptoms with selective COX-2 inhibitors reduced the risk of ailment progression and the incidence of consequent hospitalization (Consolaro et al., 2022; Ong et al., 2020).

**FIGURE 5:**
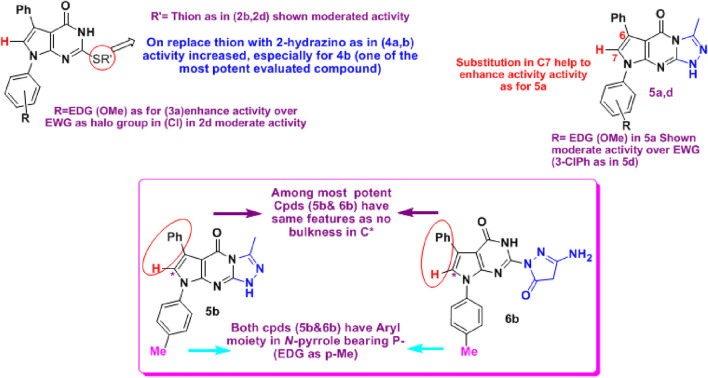
Structure correlation for tested compounds **2–7** as anti-COX-2. The results showed that **4b** (C2-hydrazino derivative), **5b** (triazolo derivative), and **6b** (C2-pyrazolonyl derivative) were among the most active compounds. In contrast, neither a phenyl group on the side of *N*-pyrrole nor *N*-(3 or 4-chlorophenyl) enhanced the activities.

In this article, we evaluated the activity of the newly synthesized pyrrole derivatives [pyrrolopyrimidines **(2–4)**, pyrrolotriazolopyrimidines **(5)**, and 2-substituted-pyrrolopyrimidines **(6–7)**] as COX inhibitors compared to indomethacin (a non-selective COX inhibitor) and celecoxib (a selective COX-2 inhibitor). From the results indicated in Table 1, all tested compounds showed selective COX-2 inhibition activity with high selectivity. **6b (2-pyrazolonyl derivative)** showed the most potent inhibitory effect, which was superior to celecoxib, followed by compound **4b**, which was equipotent to celecoxib. Both compounds showed great selectivity (SI = 797 and 260, respectively) toward the COX-2 enzyme. Compounds **3a (thio derivative), 5a, 5b (triazolo derivatives)**, and **6g (pyrazolone derivative)** showed a promising COX-2 inhibitory effect that was close to that of celecoxib. Compounds **2b, 2d, 5d, 7a**, and **7c** showed similar COX-2 inhibitory effect to the reference drug. Structure correlation is discussed in Figure 5.

**TABLE 1:** Inhibitory activities of the synthesized compounds showing activity against COX enzymes.

Compound	COX-1 [IC_50_ (µM)]	COX-2 [IC_50_ (µM)]	SI^a^
2a	5.50 ± 0.20	0.11 ± 0.005	50
2b	9.00 ± 0.10	0.09 ± 0.001	100
2c	7.00 ± 0.20	0.12 ± 0.01	58.33
2d	10.50 ± 0.20	0.07 ± 0.001	150
3a	12.50 ± 0.20	0.06 ± 0.001	208.33
3c	10.00 ± 0.20	0.15 ± 0.01	66.67
4b	13.00 ± 0.20	0.05 ± 0.001	260
5a	11.00 ± 0.20	0.06 ± 0.001	183.33
5b	11.50 ± 0.20	0.06 ± 0.003	191.67
5c	7.00 ± 0.20	0.15 ± 0.01	46.67
5d	10.00 ± 0.20	0.08 ± 0.001	125
6b	7.97 ± 0.06	0.01 ± 0.001	797
6f	13.47 ± 0.15	0.06 ± 0.001	224.5
7a	9.07 ± 0.12	0.09 ± 0.001	100.78
7b	7.00 ± 0.20	0.13 ± 0.01	53.85
7c	11.00 ± 0.10	0.07 ± 0.001	157.14
Celecoxib	14.50 ± 0.10	0.05 ± 0.002	290.00
Indomethacin	0.10 ± 0.001	0.08 ± 0.001	1.25

^a^ Data are presented as the means of three experiments ± SD.

^b^ SI: COX-1 IC_50_/COX-2 IC_50_.

^c^ Compounds **4a**, **4c**, **4d**, **6a**, **6c–e**, and **7d** showed no activity in any evaluation.

Recently, a new tactic regarding the relationship between ACE2 and COX-2 was investigated in several inflammatory pathways, including inflammatory arthritis, cancer, diabetic nephropathy, and SARS-CoV-2 infection (Shirbhate et al., 2021; Chatterjee and Thakur, 2020; Wang et al., 2017; Lin et al., 2016; Marshall et al., 2016; Elisha et al., 2016; Makene and Pool, 2015; Valenzuela et al., 2021). A good target is inhibiting the interactions between SARS‐CoV‐2 spike RBDs and ACE2 by modulating ACE2 without impairing its enzymatic activity necessary for normal physiological functions (Shin et al., 2022).

Prostaglandins (PG) and leukotrienes are released by the immune system in response to acute inflammatory activation (Lin et al., 2016; Chen et al., 2018; Zhao et al., 2022). The effect of COX-2 inhibition by NSAIDs on SARS-CoV-2 infection *via* regulating ACE2 expression was explored. It was reported that NSAID treatment did not affect ACE2 expression in mice and human cells, nor did it affect viral entry or replication *in vitro* (Chen et al., 2021). Therefore, in this work, the synthesized derivatives were screened for their potential to inhibit ACE2 receptor interactions with SARS‐CoV‐2 spike RBDs, seeking COX-2 inhibitors with ACE2 inhibitory potency. The results compared to quercetin (Liu et al., 2020) are depicted in Table 2 and revealed that 8 of 17 tested compounds were more active than quercetin: **2d, 3a, 3c, 4b, 5b, 5d, 7a**, and **7c**. Whereas **5a** (pyrrolotriazolopyrimidines bearing an EDG at the *N*-aryl moiety and a C*-7 bearing (H), not a bulky phenyl, and **6f** (**C2-pyrazolonyl derivative**) were equipotent to quercetin. **3a** and **4b** were the most potent compounds with the lowest IC_50_ (0.93 ± 0.06 µM and 1.87 ± 0.12 µM, respectively). The former compounds are the fused pyrrolopyrimidines (bearing either an alkylated thione **3a** or a hydrazine **4b** at C-2). However, the effect of the *N*-aryl and the steric effect in *C6 are not affected, which indicates that the activity is more related to the ring itself bearing a less bulky 2-substituted, and the EDG, which has a somewhat basic character.

**TABLE 2:** Inhibitory activities of the synthesized compounds against ACE2.

Compound	ACE2 [ IC_50_ (µM)]	Compound	ACE2 [ IC_50_ (µM)]
2a	8.90 ± 0.10	5b	2.90 ± 0.10
2b	5.90 ± 0.10	5c	5.90 ± 0.10
2c	7.93 ± 0.06	5d	2.87 ± 0.15
2d	2.90 ± 0.10	6b	6.90 ± 0.10
3a	0.93 ± 0.06	6f	4.93 ± 0.06
3c	1.93 ± 0.06	7a	2.90 ± 0.10
4a	6.97 ± 0.06	7b	6.87 ± 0.15
4b	1.87 ± 0.12	7c	3.97 ± 0.06
5a	4.90 ± 0.10	Quercetin	4.83 ± 0.06

^a^ Data are presented as the means of three experiments ± SD.

Numerous studies revealed that prostanoids play an important role in SARS-CoV-2 infection (Gomi et al., 2000; Zhao et al., 2011; Hata and Breyer, 2004; Sander et al., 2017). NSAIDs, including drugs containing pyrrole, suppress the synthesis of PGE2 and deactivate COX enzymes, either selectively (etodolac) or non-selectively (acemetacin, indomethacin, tolmetin, and ketorolac) (Mateev et al., 2022; Bindu et al., 2020; Jeelan Basha et al., 2022). LPS macrophage activation, which is essential for inducing inflammatory processes, has been reported by many different pathways. They are distinguished by elevated COX-2, which is responsible for the majority of PGE production (Lin et al., 2016; Vien et al., 2016). Thus, in the present study, LPS-stimulated RAW macrophages were used to investigate the anti-inflammatory efficacy of the active COX-2/ACE2 inhibitors compared to celecoxib. qRT-PCR was used to measure the pro-inflammatory cytokine production.

**FIGURE 6:**
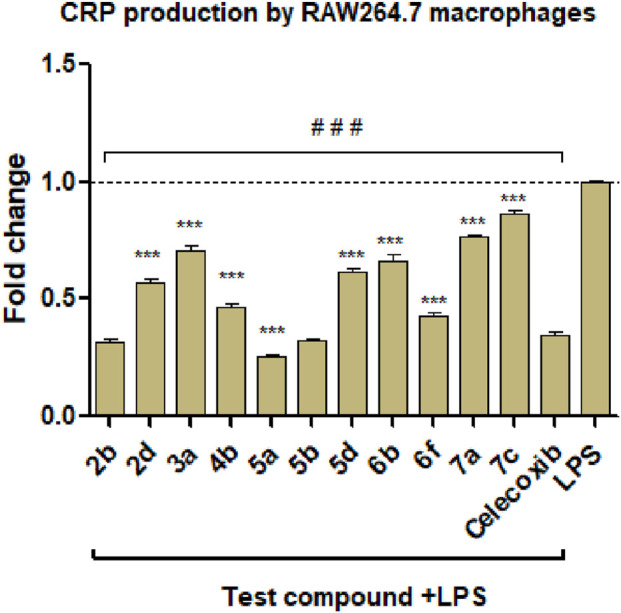
Effect of the selected compounds on CRP production by LPS-stimulated macrophages. ^###,^ ***: significant from LPS control or celecoxib at *p* < 0.001, respectively. Data are shown as mean ± SD (n = 3). CRP, C-reactive protein; LPS, lipopolysaccharide.

**FIGURE 7:**
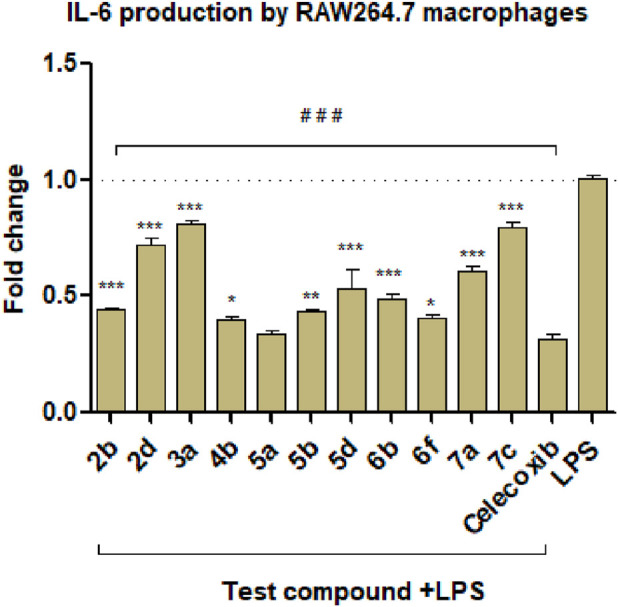
Effect of the selected compounds on IL-6 production by LPS-stimulated cells. ^###^: significant from LPS control at *p <* 0.001; *^,^ **^,^ ***: significant from celecoxib at *p* < 0.05, *p* < 0.01, or *p* < 0.001. Data are shown as mean ± SD (*n* = 3). LPS, lipopolysaccharide.

The results are depicted in Figures 6, 7 and showed that all the selected derivatives at concentrations equivalent to ¼ IC_50_ (IC_50_ values are shown in Table 3) were significantly able to block the production of CRP and IL-6 in LPS-stimulated macrophages with variable degrees compared to the control LPS-treated cells. The most active derivatives for CRP inhibition were **2b** (pyrrolopyrimidine bearing a C-2 thione group), **5a**, and **5b** (both are pyrrolotriazolopyrimidines with a *C-7 bearing H, and the *N*-aryl is EDG substituted). While for IL-6 inhibition, **2b, 4b, 5a**, **5b**, and **6f** were the most active compounds. **5a** showed the greatest inhibitory effect (75% and 66% for CRP and IL-6, respectively), which was higher than or equal to the activity of celecoxib. The anti-inflammatory activity and the cytokine production blockage shown in this study emphasize the promising activity of compounds **5a** and **5b**.

**TABLE 3:** Cytotoxicity of the selected compounds on RAW264.7 cells.

Compound	IC_50_ (µM)
**2b**	428.782 ± 17.6
**2d**	311.861 ± 12.8
**3a**	229.686 ± 9.4
**4b**	151.594 ± 6.21
**5a**	183.228 ± 7.5
**5b**	420.115 ± 17.2
**5d**	240.116 ± 9.83
**6b**	202.817 ± 8.3
**6f**	277.996 ± 11.4
**7a**	345.413 ± 14.1
**7c**	160.046 ± 6.55
Celecoxib	183.513 ± 7.51

Data are presented as the means of three experiments ± SD.

#### 2.1.2 Computational studies

##### 2.1.2.1 Physicochemical properties and toxicity properties

The SwissADME online server (http://www.swissadme.ch/, accessed on 25 March 2025) was used to calculate the physicochemical characteristics, lipophilicity, and drug-likeness of the more powerful derivatives **5a** and **5b** compared with the two standard references used (indomethacin and quercetin) (Moharr et al., 2025). The Molsoft web server (https://molsoft.com/mprop/, accessed on 24 March 2025) was used to predict the drug-likeness model score of the two derivatives **5a** and **5b** and the two reference drugs (Abdelazeem et al., 2024). Table 4 displays the anticipated and calculated outcomes.

**TABLE 4:** Physicochemical properties, lipophilicity, toxicity, and drug-likeness of the two potent derivatives **5a** and **5b** compared to the reference drugs (indomethacin for the Cox-2 assay and quercetin for the ACE2 assay).

PK parameters	Indomethacin	Quercetin	**5a**	**5b**
No. heavy atoms (HA)	25	22	28	27
No. aromatic heavy atoms (AHA)	15	16	24	24
No. H-bond acceptors (HBAs)	4	7	4	3
No. H-bond donors (HBDs)	1	5	1	1
Molar refractivity (MR)	96.12	78.03	107.33	105.81
Topological polar surface area (TPSA)	68.53 Å^2^	131.36 Å^2^	77.21 Å^2^	67.98 Å^2^
Lipophilicity				
Log *P*_o/w_ (iLOGP)	2.76	1.63	3.18	3.21
Log *P*_o/w_ (XLOGP3)	4.27	1.54	2.86	3.25
Log *P*_o/w_ (WLOGP)	3.93	1.99	3.35	3.65
Water solubility				
Log *S* (ESOL)	−4.86	−3.16	−4.38	−4.62
Class	Moderate	Soluble	Moderate	Moderate
Pharmacokinetics^1^ (green color indicates inhibitor effect)				
GI absorption	High	High	High	High
P-gp substrate	No	No	Yes	Yes
CYP1A2 inhibitor	Yes	Yes	Yes	Yes
CYP2C19 inhibitor	Yes	No	No	No
CYP2C9 inhibitor	Yes	No	Yes	Yes
CYP2D6 inhibitor	No	Yes	No	No
CYP3A4 inhibitor	No	Yes	No	No
Drug-likeness^a^				
Lipinski (Ro5)	Yes, 0 violation	Yes, 0 violation	Yes, 0 violation	Yes, 0 violation
Ghose	Yes	Yes	Yes	Yes
Veber	Yes	Yes	Yes	Yes
Egan	Yes	Yes	Yes	Yes
Bioavailability score	0.85	0.55	0.55	0.55
Drug-likeness model score^a^	0.91	0.52	0.03	0.14
pKa (basic/acidic group)	−7.46/3.70	<0.0/6.70	1.92/10.07	1.92/10.07
BBB score^b^	3.79	2.55	3.89	4.21
Toxicity properties^c^				
Ames test	Mutagen	Mutagen	Mutagen	Mutagen
Carcinogenicity in mice	Negative	Negative	Negative	Negative
Carcinogenicity in rats	Negative	Positive	Positive	Negative
hERG inhibition	Medium risk	Medium risk	Medium risk	Medium risk

^a^ https://molsoft.com/mprop/ (accessed 24 March 2025).

^b^ BBB-Score: 6, high; 0, low (DOI: 10.1021/acs.jmedchem.9b01220).

^c^ https://preadmet.webservice.bmdrc.org/toxicity/ (accessed in 24 March 2025).

^1^ 1 is the number of hydrogen bonds (acceptor or donor).

Red color indicates a non-inhibitor, mutagen, and positive carcinogenicity.

The more powerful derivatives **5a** and **5b**’s ADME attributes were predicted using the pkCSM web server (http://biosig.unimelb.edu.au/pkcsm/prediction, viewed on 24 March 2025). The PreADMET web server (https://preadmet.bmdrc.kr/, accessed on 24 March 2025) was also used to estimate the toxicity parameters of the investigated substances. Table 4 displays the anticipated and calculated outcomes of the ADME and toxicity characteristics prediction.

A comprehensive description of the drugs’ physicochemical characteristics is necessary to comprehend their biological and therapeutic effects (Hassan et al., 2023; Hassan et al., 2021). Therefore, in the drug discovery phases, the physicochemical qualities are required for the evaluation process to determine drug-likeness and select oral bioactive candidates. From the former data, the two triazolopyrimidines, **5a** and **5b**, are in compliance with the Lipinski, Ghose, Veber, and Egan guidelines, according to the findings of the drug-likeness evaluation. The compounds also have a favorable score when compared to the two reference standards, quercetin and indomethacin, and using the drug-likeness model score evaluation rule, like two FDA-approved medications. Positive scores are present in both **5a** and **5b**, as seen in Table 4. As a result, these substances could be considered promising drug-like compounds.

### 2.2 Molecular docking and molecular dynamics results and discussion

#### 2.2.1 Molecular docking with COX-2 enzyme for the most active compounds, **5a** and **5b**

**FIGURE 8:**
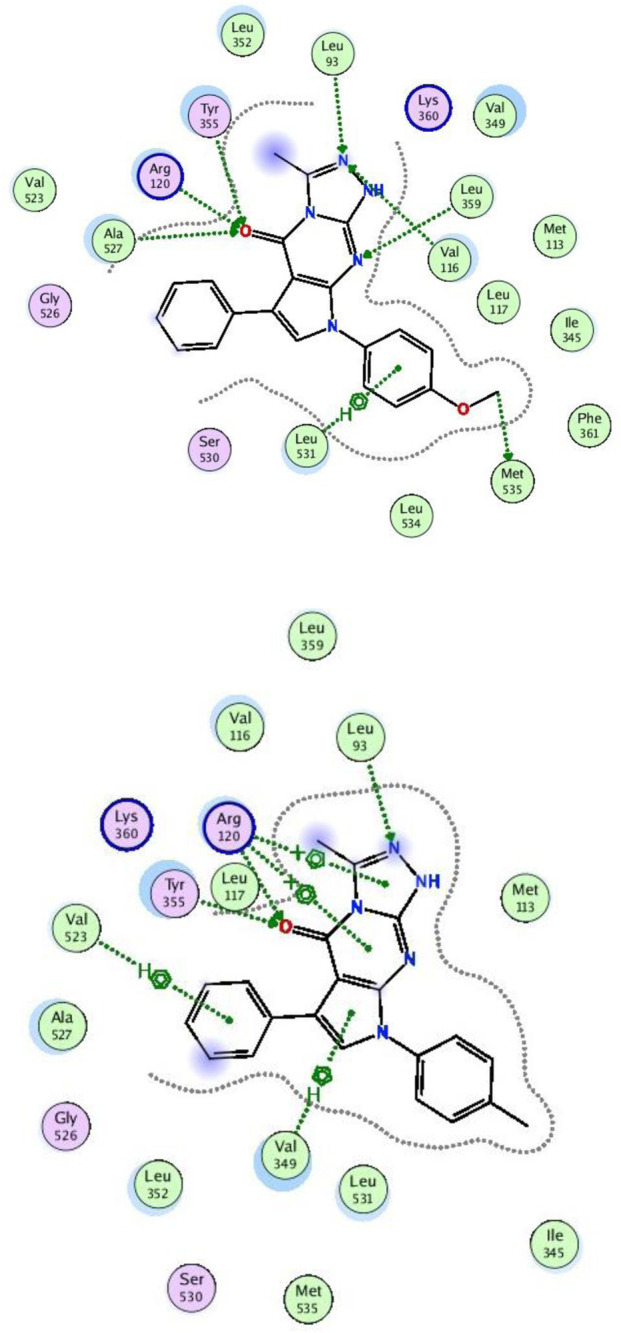
Docking of compounds **5a** (upper) and **5b** (lower) with COX-2 using PDB ID: 3NTG.

To understand the biological results of our active compounds, a molecular docking study was performed using MOE 2019.02 software. Molecular docking screenings were performed after achieving synthesis and characterization of the all-new compounds. Consequently, compounds **5a** and **5b** were subjected to docking analysis within the pre-defined active site of COX-2. The binding affinity of the highly active compounds was calculated inside the enzyme binding sites (Facchini et al., 2018; Wu et al., 2020; ul Ain et al., 2020), as shown in Figure 8.

Interestingly, both compounds **5a** and **5b** demonstrated favorable binding with COX-2, with binding patterns that closely resembled that of the reported pattern. The synthesized compounds **5a** and **5b** achieved docking scores of −12.1 kcal/mol and −11.8 kcal/mol, respectively, which closely approximated the docking score of the co-crystalized ligand (−12.8 kcal/mol). As illustrated in Figure 8, the carbonyl group compound **5a** served as a hydrogen bond acceptor, forming three hydrogen bonds with the key residues Arg120, Tyr355, and Ala527. The nitrogen atoms in the fused ring system formed three hydrogen bonds with Leu93, Val116, and Leu359. In addition, the phenoxy ring of compound **5a** participated in two interactions, one hydrophobic interaction with Leu531 and a hydrogen bond with Met535, through the phenyl ring and the methoxy group, respectively.

Similarly, compound **5b** formed dual hydrogen bonds through its carbonyl group with crucial residues Arg120 and Tyr355. The fused hetero ring system formed one hydrogen bond interaction with Leu93 and two hydrophobic interactions with Arg120 and Val349. Finally, the phenyl ring formed one hydrophobic interaction with Val523.

#### 2.2.2 Molecular docking of the most potent compounds **5a** and **5b** on the ACE2 enzyme

**FIGURE 9:**
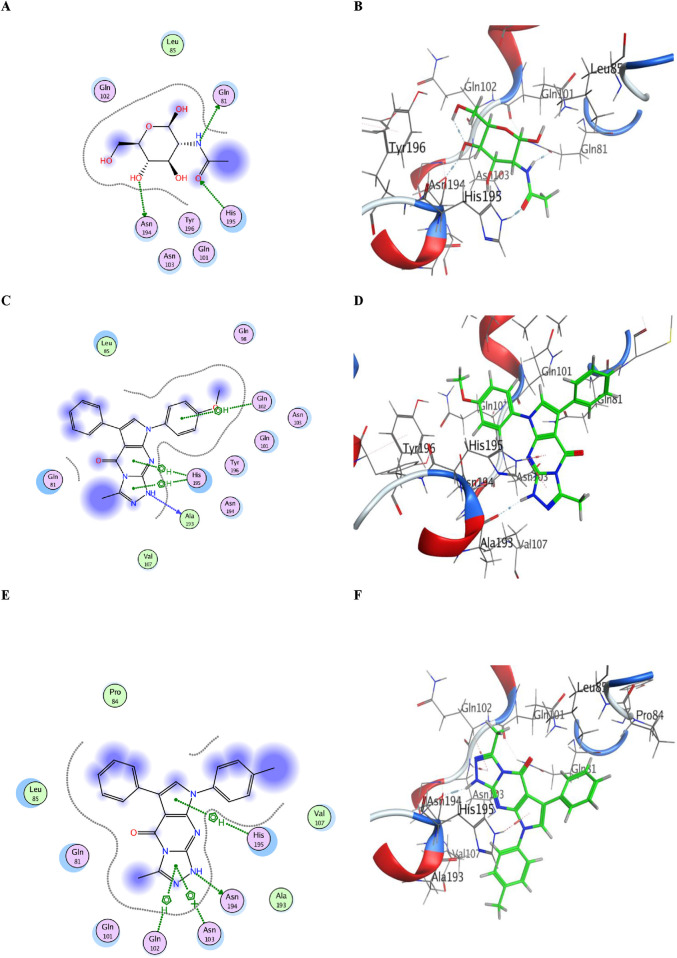
**(A)** Two-dimensional view of the redocked ligand (NAG) on ACE2 (PDB: 1R42). **(B)** Three-dimensional view of the redocked ligand (NAG) on ACE2. **(C)** Two-dimensional view of compound **5a** on the ACE2 active site. **(D)** Three-dimensional view of compound **5a** on ACE2 active site. **(E)** Two-dimensional view of compound **5b** on the ACE2 active site. **(F)** Three-dimensional view of compound **5b** on the ACE2 active site.

Because ACE2 is an essential receptor for this virus’s cell entrance (Moharr et al., 2025; Basu et al., 2020), one viable therapeutic strategy for creating medications against SARS-CoV-2 is to block the binding location of the virus on human ACE2. Here, we used docking research to find novel SARS-CoV-2 inhibitors by examining the binding affinity for the most powerful compounds, **5a** and **5b**, in the ACE2 active site (PDB: 1R42). MOE 2014.09 (Lattuca et al., 2013) was used to conduct the docking study against the human ACE2 active site (PDB: 1R42). The most significant residues that connect with ACE2 (PDB: 1R42) to stop SARS-CoV-2 and ACE2 interactions were determined from the literature. NAG, the co-crystallized ligand, was selected in accordance with the medications’ established properties and indications as ACE inhibitors (Park and Eun, 2022). The co-crystallized ligand, NAG, was redocked to verify the docking process. The resulting (RMSD) value between the ligand and the redocked posture was 1.215 Å, indicating the docking’s reliability. The disclosed ligand was compared with the docking results of the active produced molecules **5a** and **5b**. Figure 9 shows the energy binding scores and binding interaction outcomes.

The redocked ligand (NAG) on ACE2 (PDB: 1R42) revealed that the most important amino acids are Gln81, His195, and Asn194 through the formation of three hydrogen bonds with a docking score of S = −4.6019 kcal/mol. This finding can be compared with that of compound **5a**, which had a docking score of S = −5.1895 kcal/mol and successfully bound with three hydrophobic interactions, two with His195 (through its triazolopyrimidine moiety) and the third with Gln102 (through its *N7*-pyrrole aryl moiety). In addition, **5a** showed an additional binding site in the active pocket involving the amino acid Ala193 (through hydrogen bonding with its *N1*-atom).

For compound **5b**, the docking score was S = −5.1972 kcal/mol, and it had four binding sites, namely, three hydrophobic interactions with His195 (through the pyrrole ring), Gln102, and Asn194 (through the triazole ring). One extra binding site over the ligand was the hydrogen bond between *N1* in **5b** and the amino acid Asn103. A table with full details about the ACE2 docking experiment is included in the S2 file, Materials and methods.

#### 2.2.3 Molecular dynamics

**FIGURE 10:**
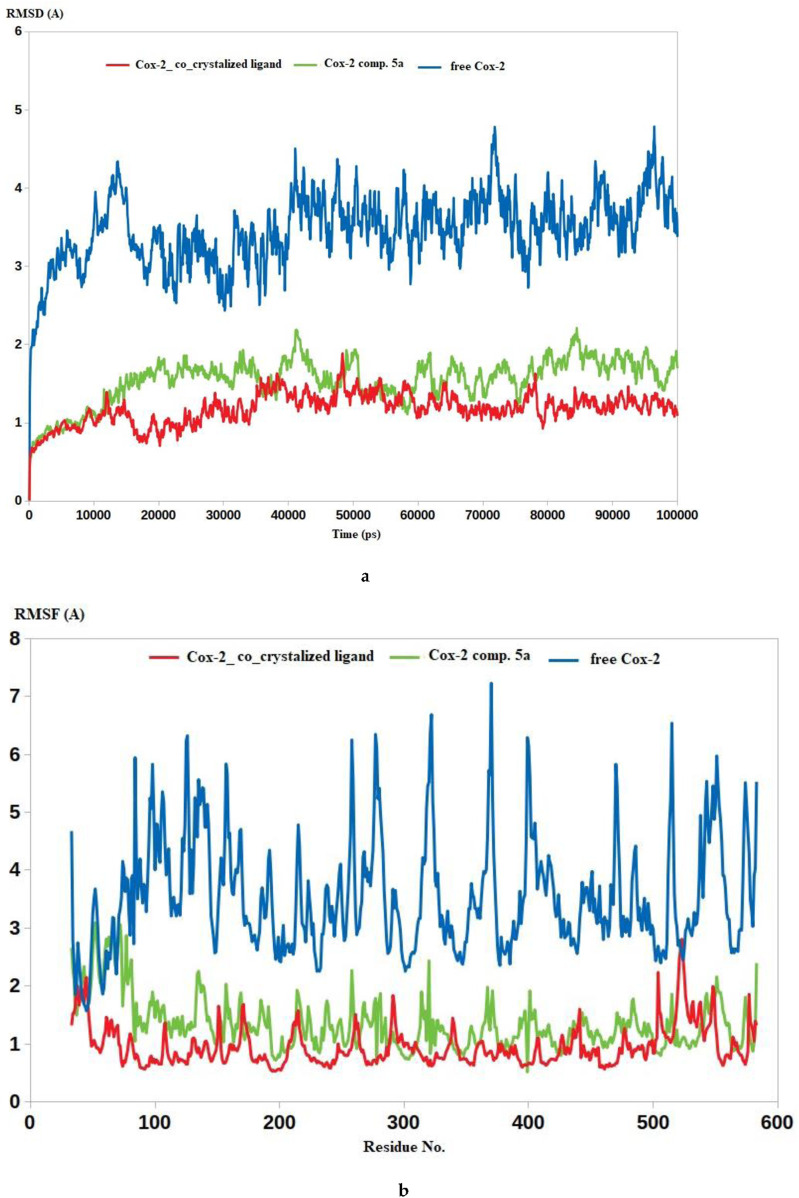
**(a)** RMSD analysis for the MD simulations. **(b)** RMSF analysis for the MD simulations.

Additional computational analyses were conducted through molecular dynamics simulations. Molecular dynamics (MD) simulations offer valuable information and parameters for studying the dynamic behavior of biological systems. Among these details, MD can offer insights into the precise assessment of the binding strength of a docked complex involving a ligand and a target. Consequently, the predicted binding coordinates obtained from the docking of COX-2 with **5a** and the co-crystalized ligand were further subjected to MD simulation. To establish a comparative basis for evaluating the impact of each ligand on the stability of the COX-2 enzyme, the latter was subjected to molecular dynamics simulation (MDS) using the Apo form. As illustrated in Figure 10a, both inhibitors effectively stabilized the COX-2 enzyme, as indicated by their lower root mean square deviation (RMSD) values than the RMSD value of Apo COX-2. The COX-2-5a complex displayed RMSD values of 1.7 Å, which are highly comparable to that of COX-2-co-crystalized ligand (1.4 Å), while the RMSD of Apo COX-2 reached 3.8 Å. The ability of compound **5a** to restrict the dynamic nature of COX-2by forming stable complexes, as indicated by the lower RMSD values, serves as a valid indicator of its inhibitory impact on COX-2, as revealed in Figure 10a.

To further support the RMSD calculations, the root mean square fluctuation (RMSF) values for all residues in the three systems were computed. As anticipated, the RMSF values corroborated the conclusions drawn from the RMSD calculations, wherein the average RMSF of Apo COX-2 residues reached 4.3 Å, while the average RMSF values for COX-2 residues in complex with **5a** and the co-crystalized ligand reached averages of 1.6 Å and 1.2 Å, respectively (Figure 10b). In summary, both RMSD and RMSF values validated the proposed binding mode between **5a** with the COX-2 active site, attributing its inhibitory activity to the ability to form a stable complex with COX-2.

## 3 Materials and methods

The detailed methods and protocols for the synthetic scheme, biological studies, computational studies, molecular docking, and molecular dynamics, as well as statistical analysis, are provided in the Supplementary Material.

## 4 Conclusion

Nonsteroidal anti-inflammatory drugs (NSAIDs) have long been recognized as valuable agents in modulating hyper-inflammatory responses due to their ability to control cytokine production without the immunosuppressive drawbacks of corticosteroids. Building on this foundation, we designed and synthesized novel 2-hydrazinopyrrolopyrimidines and their fused pyrazolo derivatives (**5a–d** and **6–7**) to act as dual-target inhibitors with selective COX-2 activity and additional ACE2 blocking potential. The biological evaluation demonstrated that most of the synthesized derivatives exhibited strong selectivity toward COX-2, while several also showed significant ACE2 inhibition. Compounds **5a** and **5b**, in particular, displayed potent dual activity, supported by cytokine suppression assays (CRP and IL-6), as well as molecular docking and dynamics simulations that confirmed stable binding within both COX-2 and ACE2 active sites. These results highlight the value of pyrrolopyrimidine scaffolds as promising candidates in the development of next-generation anti-inflammatory agents. By integrating selective COX-2 inhibition with ACE2 modulation, these compounds may provide therapeutic benefit not only in viral contexts but also in broader inflammation-driven disorders. Our findings, therefore, support the continued exploration of multi-target NSAIDs as a versatile strategy in medicinal chemistry and drug discovery.

## Data Availability

The original contributions presented in the study are included in the article/Supplementary Material; further inquiries can be directed to the corresponding author.
