# Supplementary material for: Pyrrolopyrimidine derivatives as dual COX-2/ACE2 inhibitors: design, synthesis, and anti-inflammatory evaluation
Source: Front Mol Biosci. 2026 Jan 6;12:1710650. doi: 10.3389/fmolb.2025.1710650 (PMC12815825; doi:10.3389/fmolb.2025.1710650)
Supplement: Supplementary file 1 [file DataSheet1.pdf]

Supplementary Materials

(Article.)

Pyrrolopyrimidine Derivatives as Dual COX-2/ACE2 Inhibitors: Design, Synthesis, and Anti-Inflammatory Evaluation

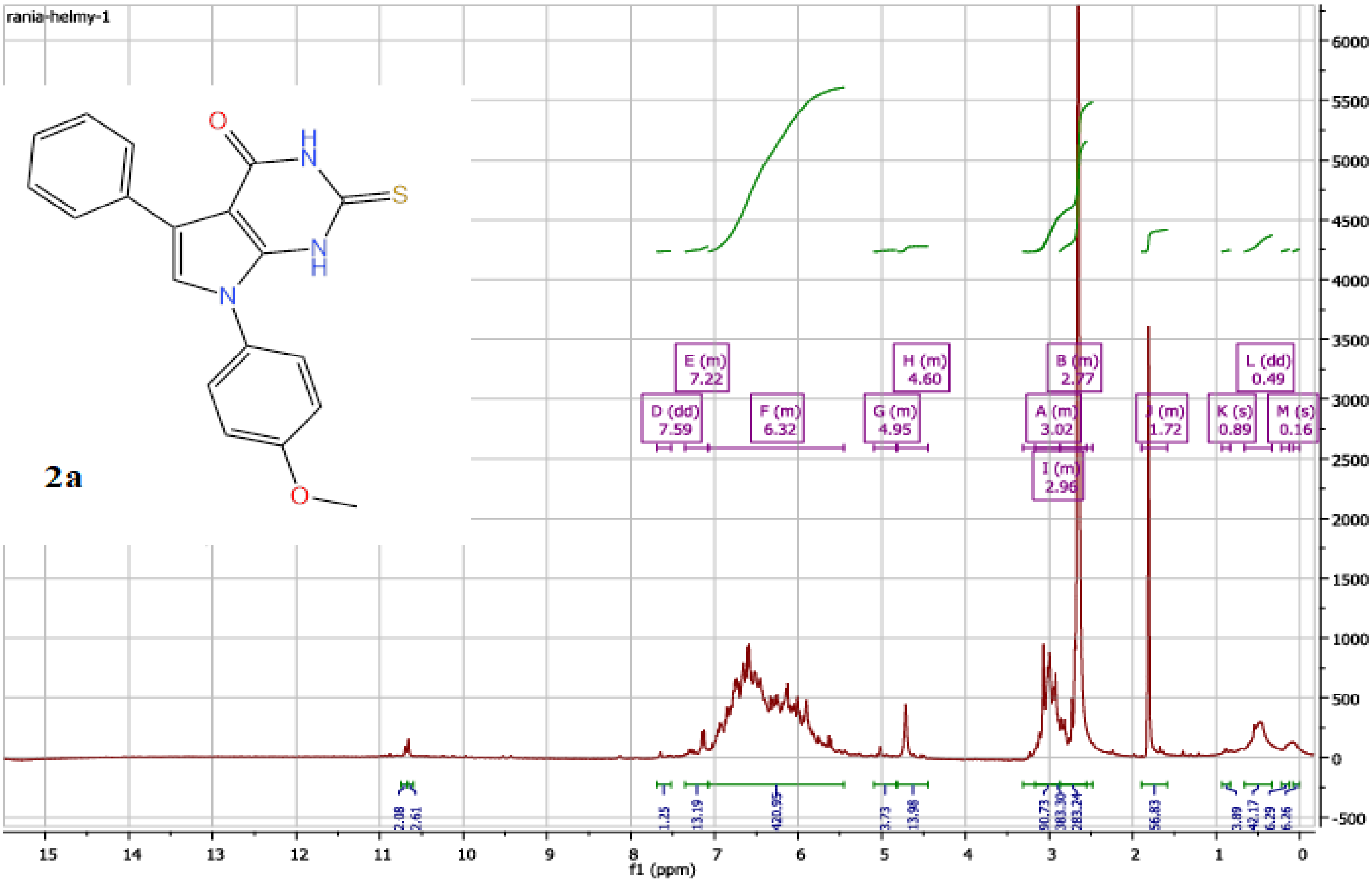
rania-helmy-1
2a
E (m) 7.22
H (m) 4.60
B (m) 2.77
L (dd) 0.49
D (dd) 7.59
F (m) 6.32
G (m) 4.95
A (m) 3.02
J (m) 1.72
K (s) 0.89
M (s) 0.16
I (m) 2.96
2.08
2.61
1.25
13.19
420.95
3.73
13.98
90.73
383.30
283.24
56.83
3.89
42.17
6.29
6.26
f1 (ppm)

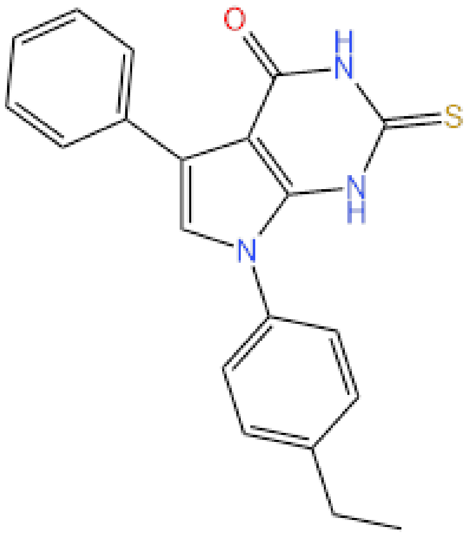

2b

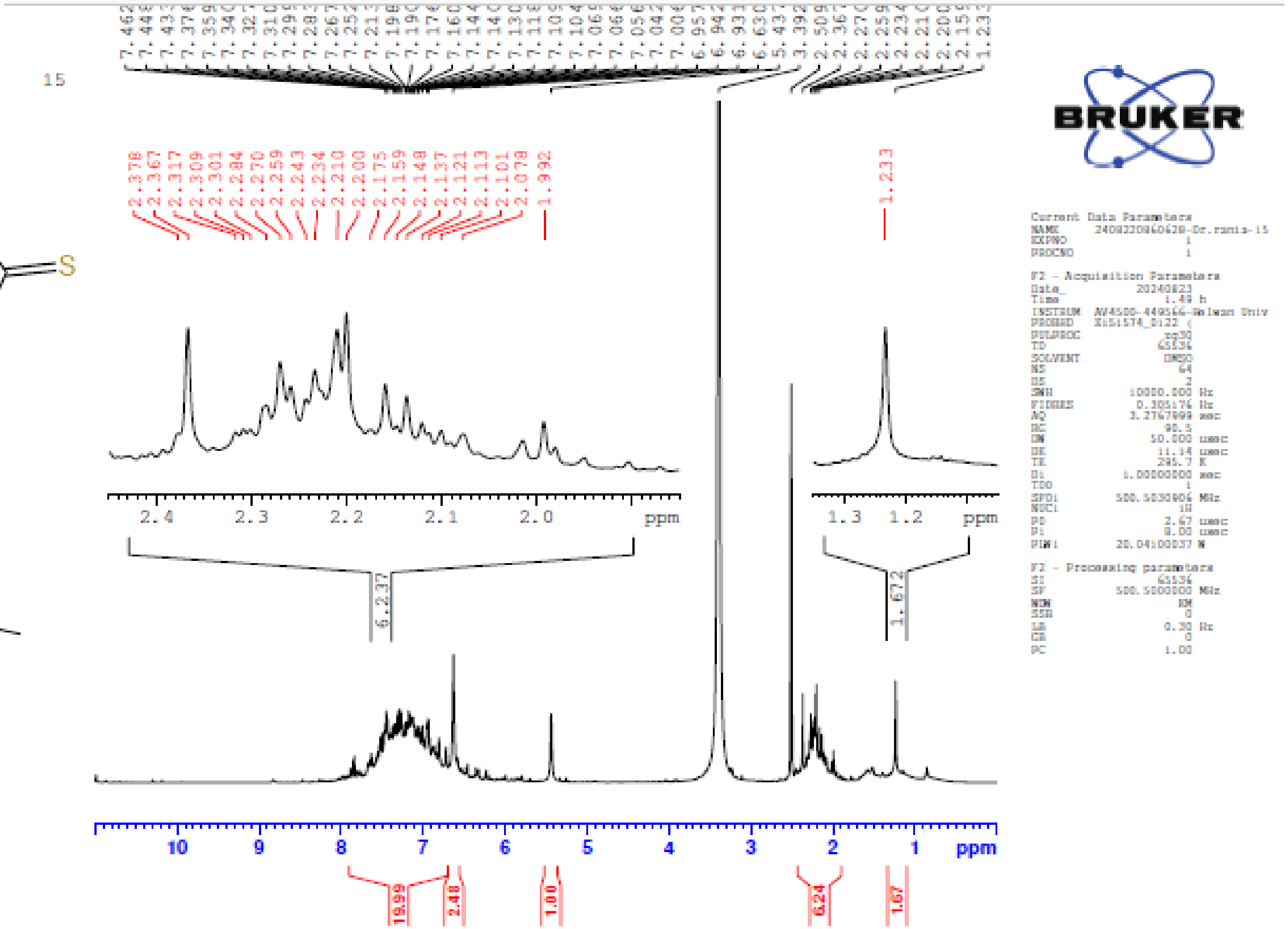

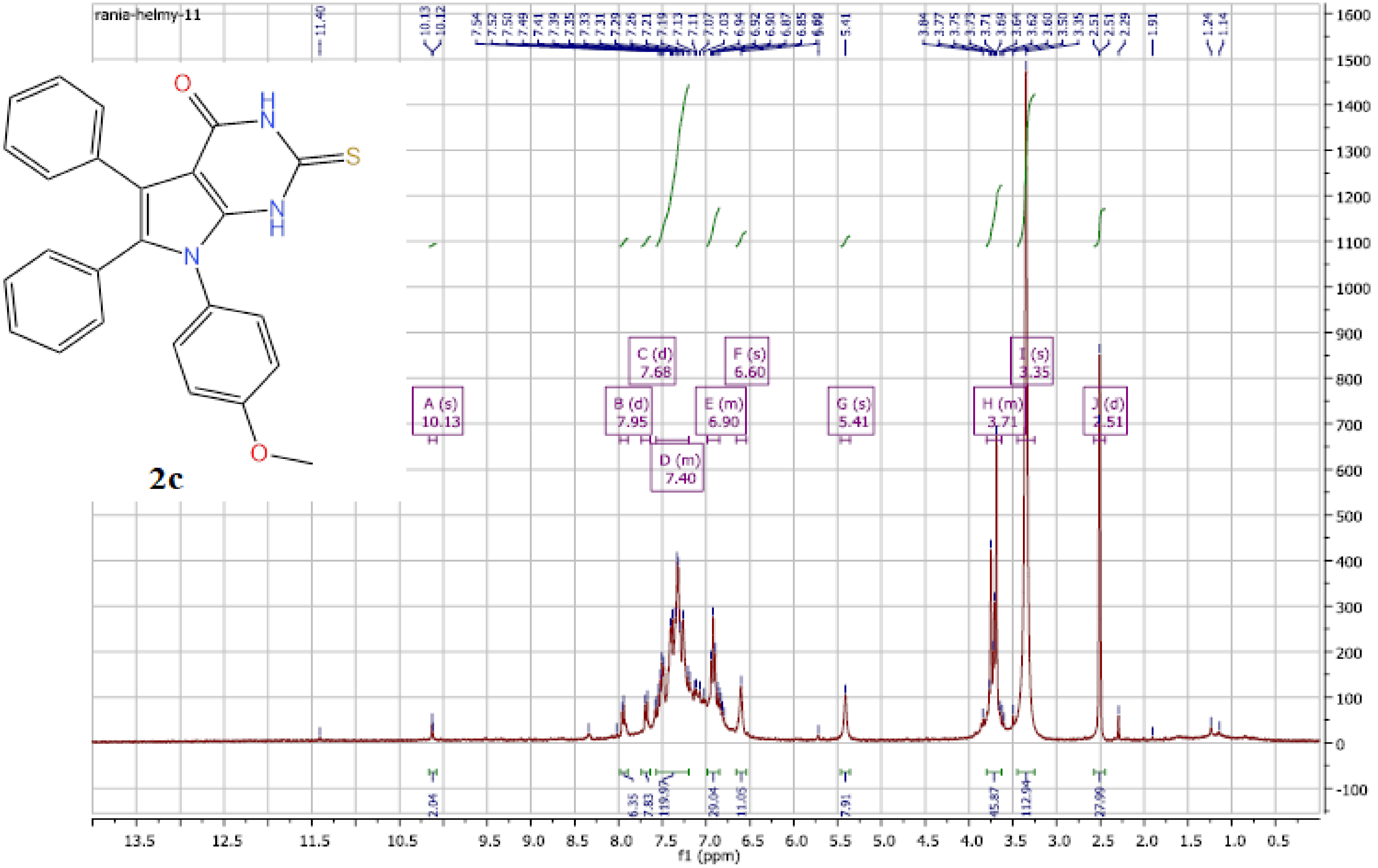
rania-helmy-11
2c
A (s) 10.13
B (d) 7.95
C (d) 7.68
D (m) 7.40
E (m) 6.90
F (s) 6.60
G (s) 5.41
H (m) 3.71
I (s) 3.35
J (d) 2.51
f1 (ppm)

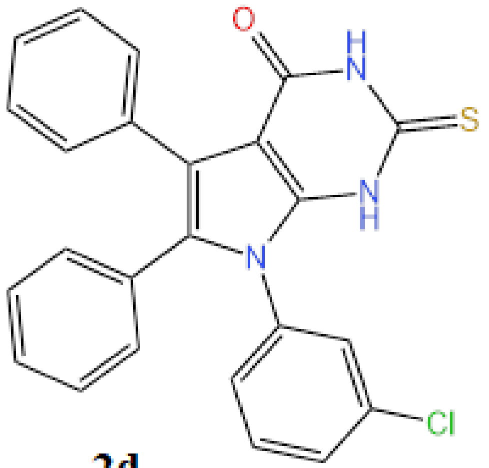
O
H
N
S
N
H
N
Cl

2d

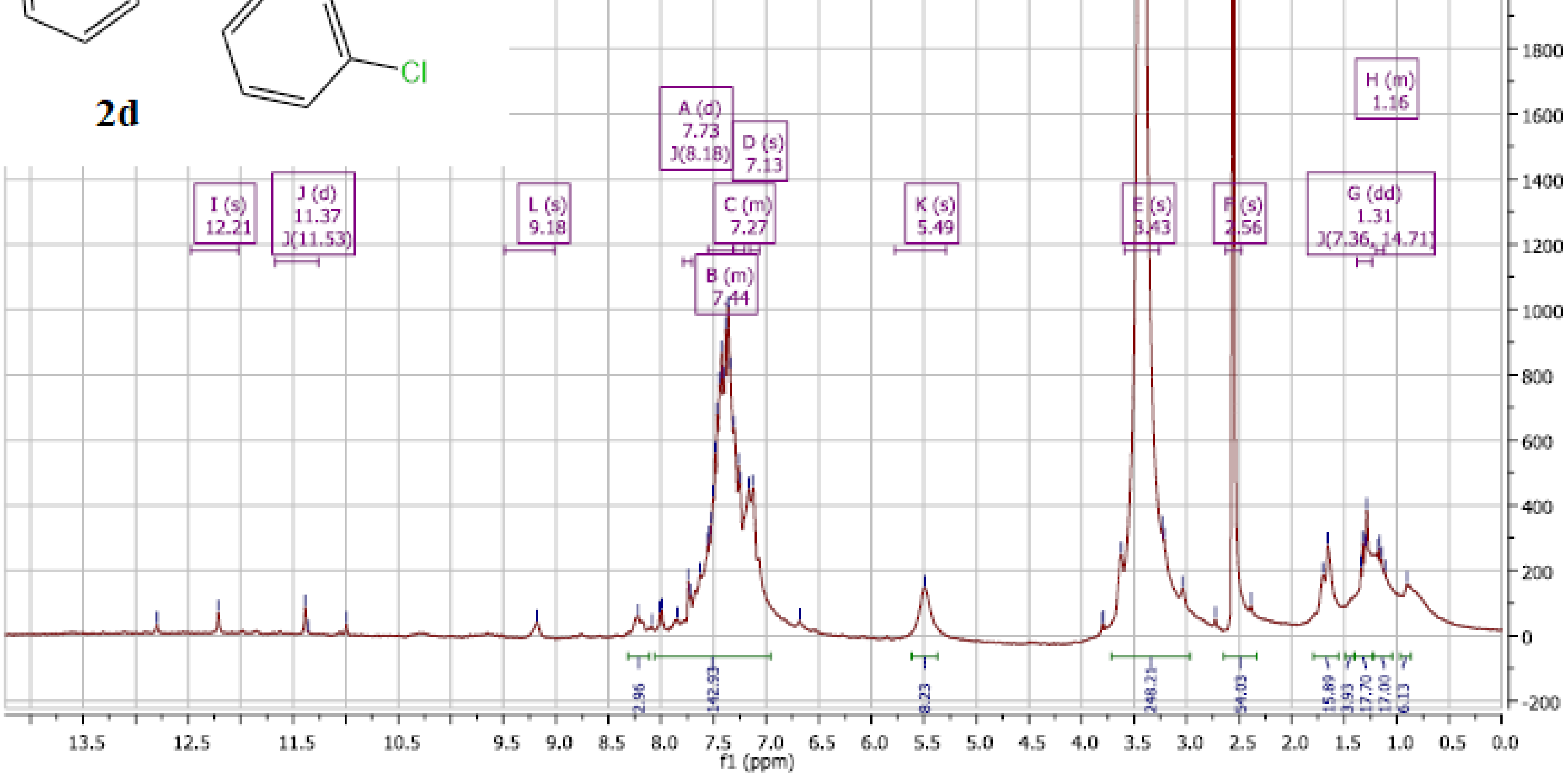
A (d)
7.73
J(8.18)
D (s)
7.13
C (m)
7.27
B (m)
7.44
I (s)
12.21
J (d)
11.37
J(11.53)
L (s)
9.18
K (s)
5.49
E (s)
3.43
F (s)
2.56
G (dd)
1.31
J(7.36, 14.71)
H (m)
1.16
2.96
142.93
8.23
248.21
54.03
15.89
3.93
17.70
17.00
6.13
f1 (ppm)

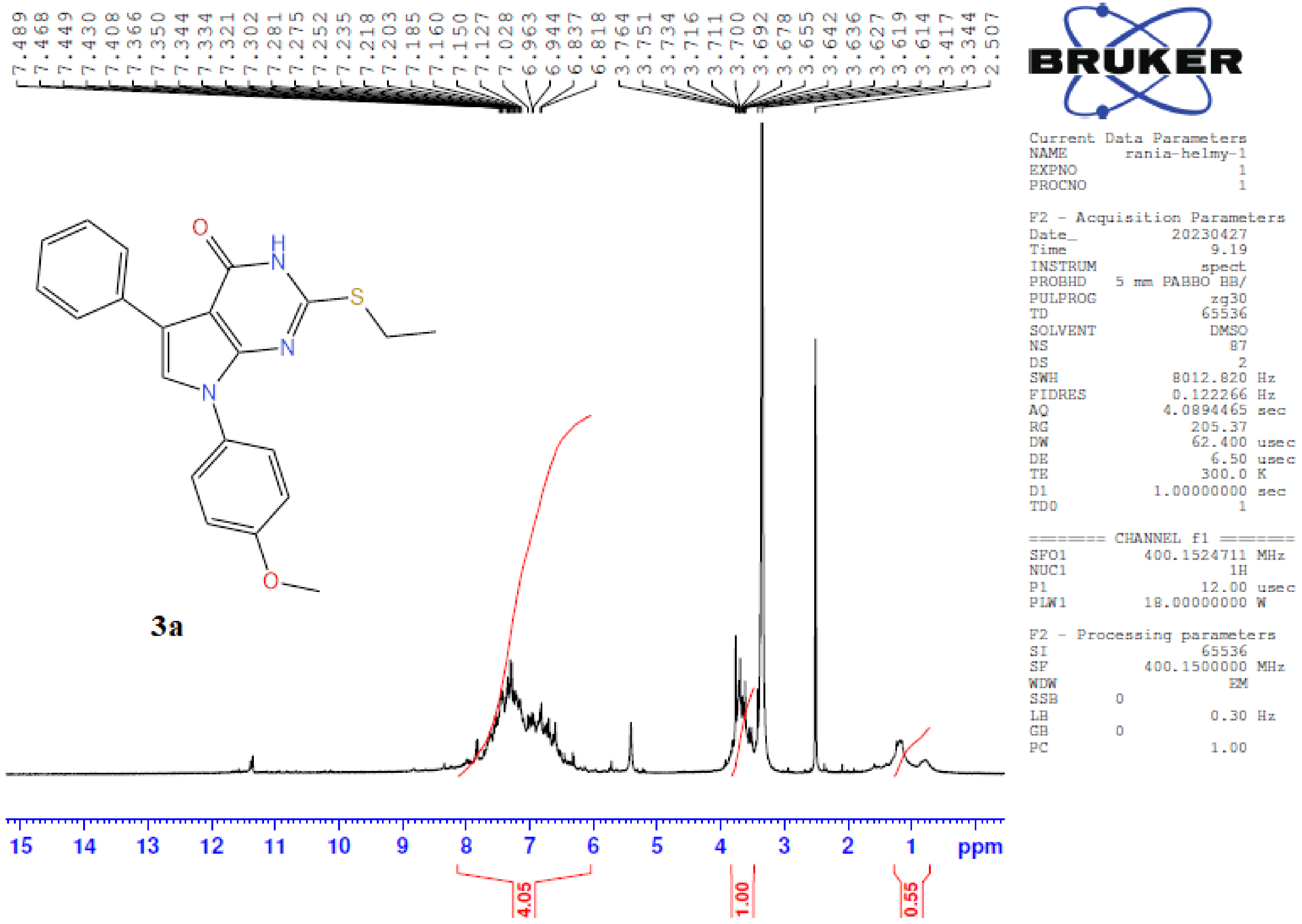
7.489 7.468 7.449 7.430 7.408 7.366 7.350 7.344 7.334 7.321 7.302 7.281 7.275 7.252 7.235 7.218 7.203 7.185 7.160 7.150 7.127 7.028 6.963 6.944 6.837 6.818 3.764 3.751 3.734 3.716 3.711 3.700 3.692 3.678 3.655 3.642 3.636 3.627 3.619 3.614 3.417 3.344 2.507
O
H
N
S
N
N
O
BRUKER
Current Data Parameters
NAME rania-helmy-1
EXPNO 1
PROCNO 1
F2 - Acquisition Parameters
Date_ 20230427
Time 9.19
INSTRUM spect
PROBHD 5 mm PABBO BB/
PULPROG zg30
TD 65536
SOLVENT DMSO
NS 87
DS 2
SWH 8012.820 Hz
FIDRES 0.122266 Hz
AQ 4.0894465 sec
RG 205.37
DW 62.400 usec
DE 6.50 usec
TE 300.0 K
D1 1.00000000 sec
TD0 1
======= CHANNEL f1 =======
SFO1 400.1524711 MHz
NUC1 1H
P1 12.00 usec
PLW1 18.00000000 W
F2 - Processing parameters
SI 65536
SF 400.1500000 MHz
WDW EM
SSB 0
LB 0.30 Hz
GB 0
PC 1.00
15 14 13 12 11 10 9 8 7 6 5 4 3 2 1 ppm
4.05
1.00
0.55

3a

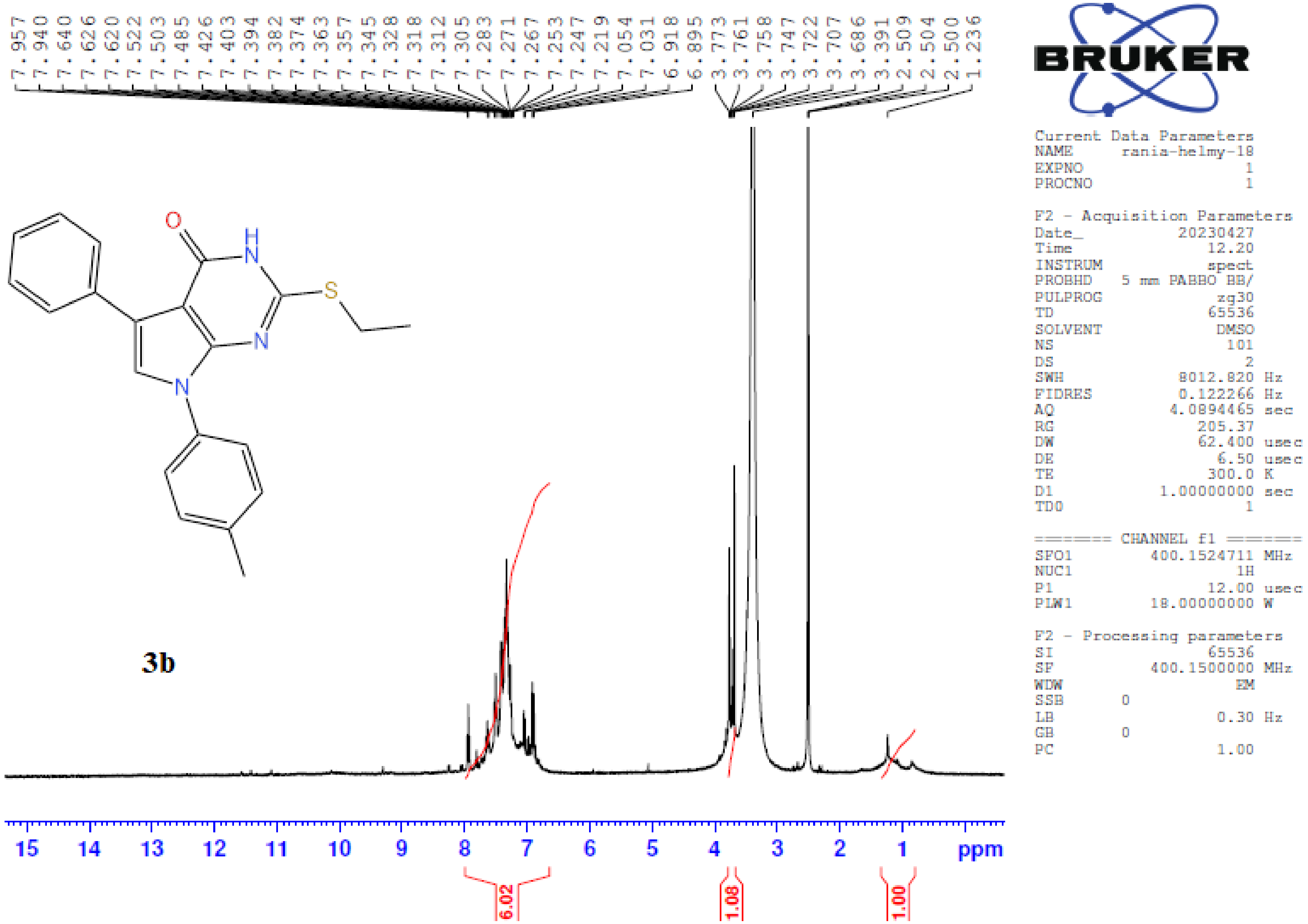
7.957
7.940
7.640
7.626
7.620
7.522
7.503
7.485
7.426
7.403
7.394
7.382
7.374
7.363
7.357
7.345
7.328
7.318
7.312
7.305
7.283
7.271
7.267
7.253
7.247
7.219
7.054
7.031
6.918
6.895
3.773
3.761
3.758
3.747
3.722
3.707
3.686
3.391
2.509
2.504
2.500
1.236
O
H
N
S
N
N
3b
15 14 13 12 11 10 9 8 7 6 5 4 3 2 1 ppm
6.02
1.08
1.00
BRUKER
Current Data Parameters
NAME rania-helmy-18
EXPNO 1
PROCNO 1
F2 - Acquisition Parameters
Date_ 20230427
Time 12.20
INSTRUM spect
PROBHD 5 mm PABBO BB/
PULPROG zg30
TD 65536
SOLVENT DMSO
NS 101
DS 2
SWH 8012.820 Hz
FIDRES 0.122266 Hz
AQ 4.0894465 sec
RG 205.37
DW 62.400 usec
DE 6.50 usec
TE 300.0 K
D1 1.00000000 sec
TD0 1
======== CHANNEL f1 ========
SFO1 400.1524711 MHz
NUC1 1H
P1 12.00 usec
PLW1 18.00000000 W
F2 - Processing parameters
SI 65536
SF 400.1500000 MHz
WDW EM
SSB 0
LB 0.30 Hz
GB 0
PC 1.00

3c

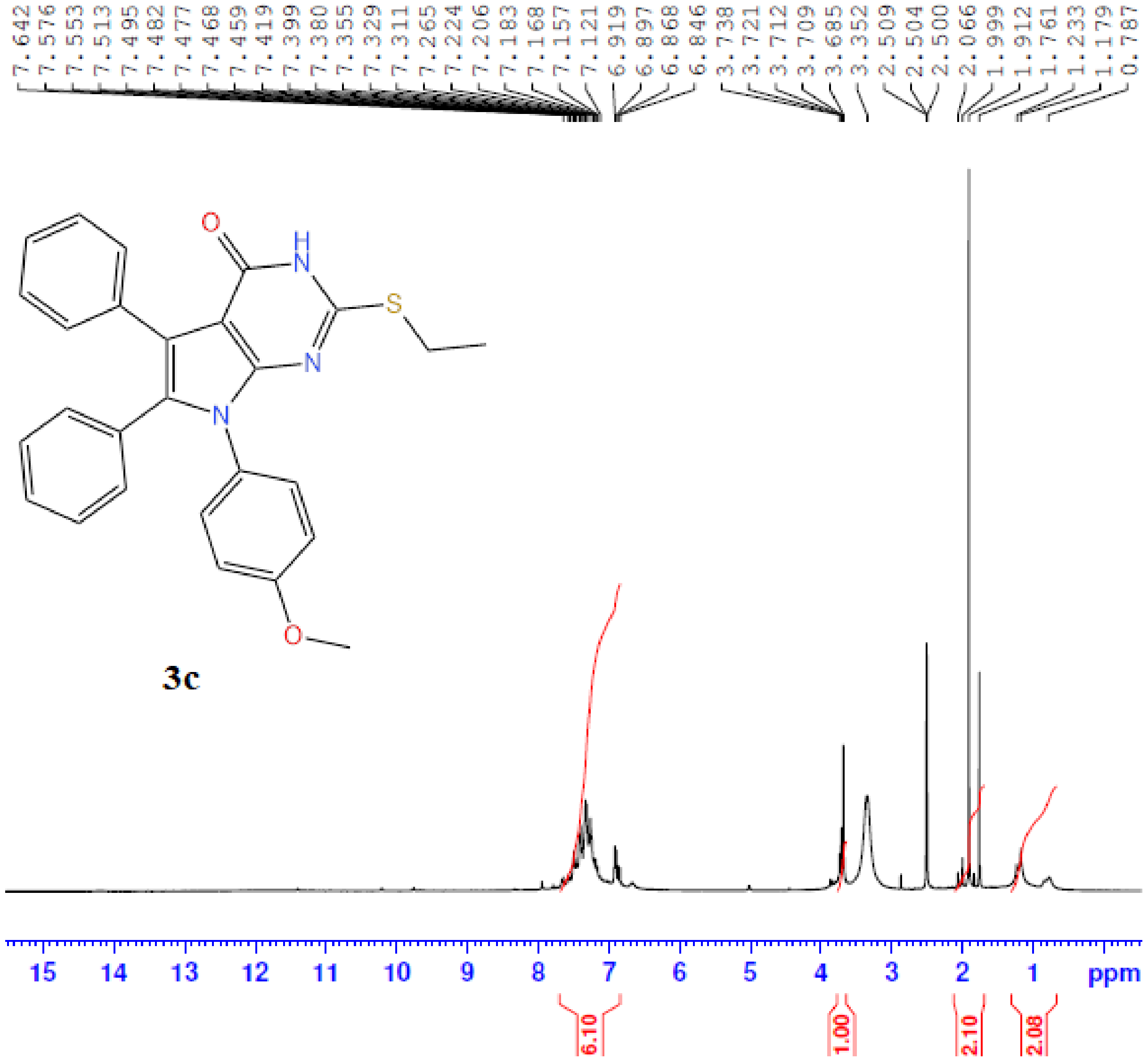

BRUKER

Current Data Parameters
NAME rania-helmy-14
EXPNO 1
PROCNO 1

F2 - Acquisition Parameters
Date_ 20230427
Time 11.56
INSTRUM spect
PROBHD 5 mm PABBO BB/
PULPROG zg30
TD 65536
SOLVENT DMSO
NS 96
DS 2
SWH 8012.820 Hz
FIDRES 0.122266 Hz
AQ 4.0894465 sec
RG 205.37
DW 62.400 usec
DE 6.50 usec
TE 300.0 K
D1 1.00000000 sec
TD0 1

======== CHANNEL f1 ========
SFO1 400.1524711 MHz
NUC1 1H
P1 12.00 usec
PLW1 18.00000000 W

F2 - Processing parameters
SI 65536
SF 400.1500000 MHz
WDW EM
SSB 0
LB 0.30 Hz
GB 0
PC 1.00

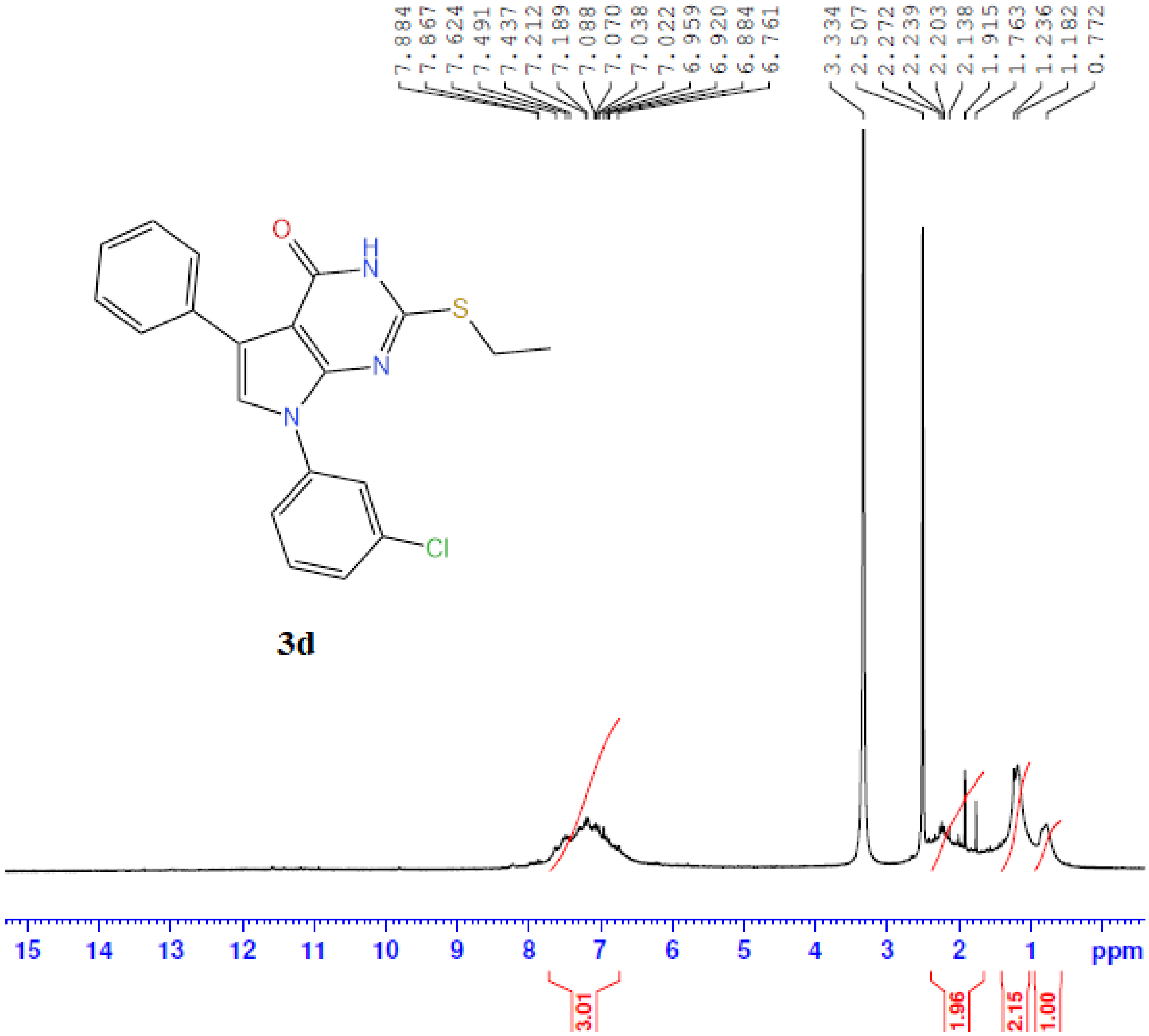


```
Current Data Parameters
NAME          rania-helmy-3
EXPNO                     1
PROCNO                    1

F2 - Acquisition Parameters
Date_              20230427
Time                   9.46
INSTRUM               spect
PROBHD    5 mm PABBO BB/
PULPROG                zg30
TD                    65536
SOLVENT                DMSO
NS                      102
DS                        2
SWH                8012.820 Hz
FIDRES             0.122266 Hz
AQ                4.0894465 sec
RG                   205.37
DW                   62.400 usec
DE                     6.50 usec
TE                    300.0 K
D1               1.00000000 sec
TD0                       1

======= CHANNEL f1 =======
SFO1         400.1524711 MHz
NUC1                     1H
P1                    12.00 usec
PLW1         18.00000000 W

F2 - Processing parameters
SI                    65536
SF              400.1500000 MHz
WDW                      EM
SSB      0
LB                     0.30 Hz
GB       0
PC                     1.00
```

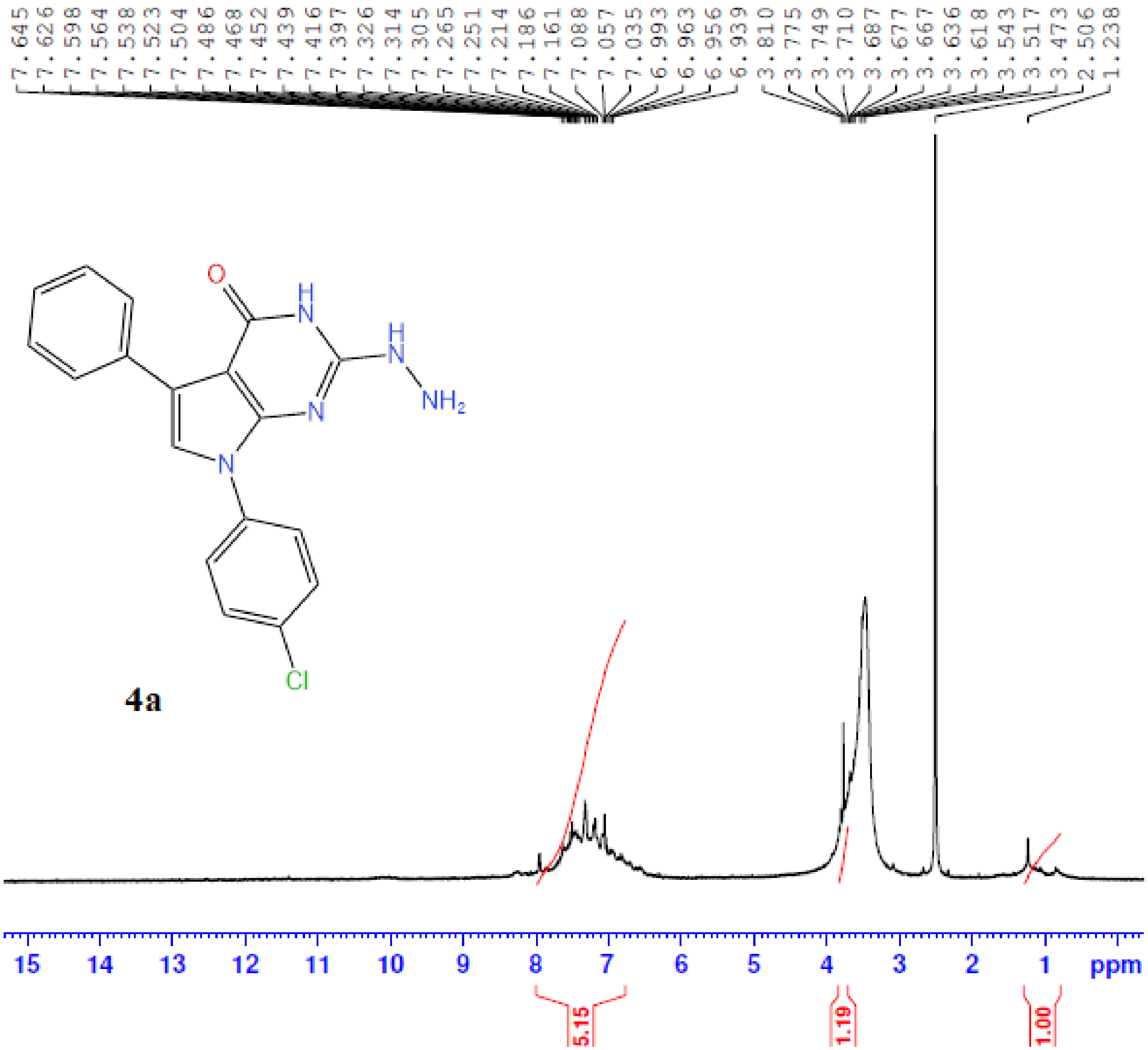

BRUKER

Current Data Parameters
NAME rania-helmy-6
EXPNO 1
PROCNO 1

F2 - Acquisition Parameters
Date_ 20230427
Time 10.11
INSTRUM spect
PROBHD 5 mm PABBO BB/
PULPROG zg30
TD 65536
SOLVENT DMSO
NS 107
DS 2
SWH 8012.820 Hz
FIDRES 0.122266 Hz
AQ 4.0894465 sec
RG 205.37
DW 62.400 usec
DE 6.50 usec
TE 300.0 K
D1 1.00000000 sec
TD0 1

======= CHANNEL f1 =======
SFO1 400.1524711 MHz
NUC1 1H
P1 12.00 usec
PLW1 18.00000000 W

F2 - Processing parameters
SI 65536
SF 400.1500000 MHz
WDW EM
SSB 0
LB 0.30 Hz
GB 0
PC 1.00

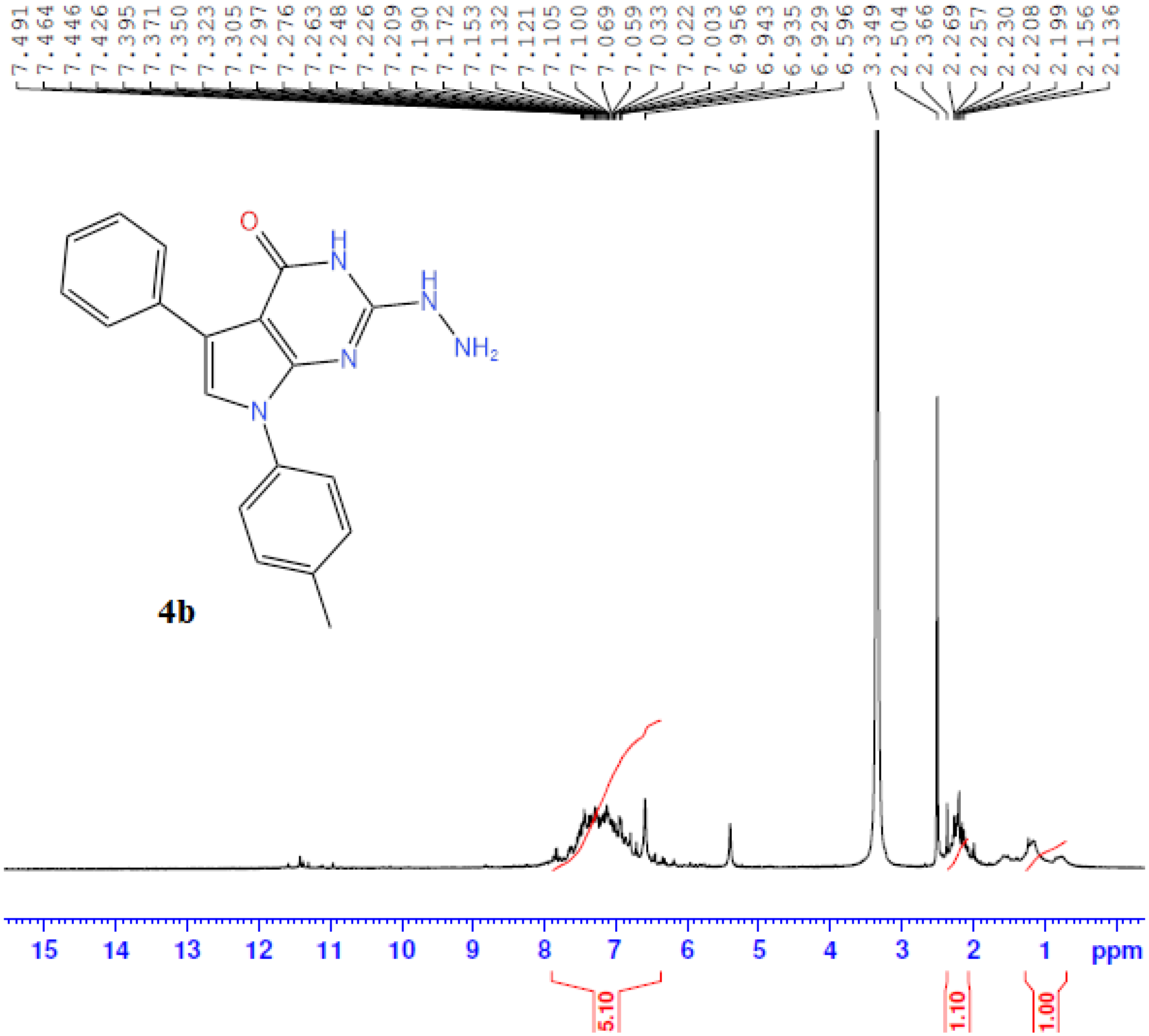

BRUKER

Current Data Parameters
NAME rania-helmy-15
EXPNO 1
PROCNO 1

F2 - Acquisition Parameters
Date_ 20230427
Time 12.09
INSTRUM spect
PROBHD 5 mm PABBO BB/
PULPROG zg30
TD 65536
SOLVENT DMSO
NS 112
DS 2
SWH 8012.820 Hz
FIDRES 0.122266 Hz
AQ 4.0894465 sec
RG 205.37
DW 62.400 usec
DE 6.50 usec
TE 300.0 K
D1 1.00000000 sec
TD0 1

======= CHANNEL f1 =======
SFO1 400.1524711 MHz
NUC1 1H
P1 12.00 usec
PLW1 18.00000000 W

F2 - Processing parameters
SI 65536
SF 400.1500000 MHz
WDW EM
SSB 0
LB 0.30 Hz
GB 0
PC 1.00

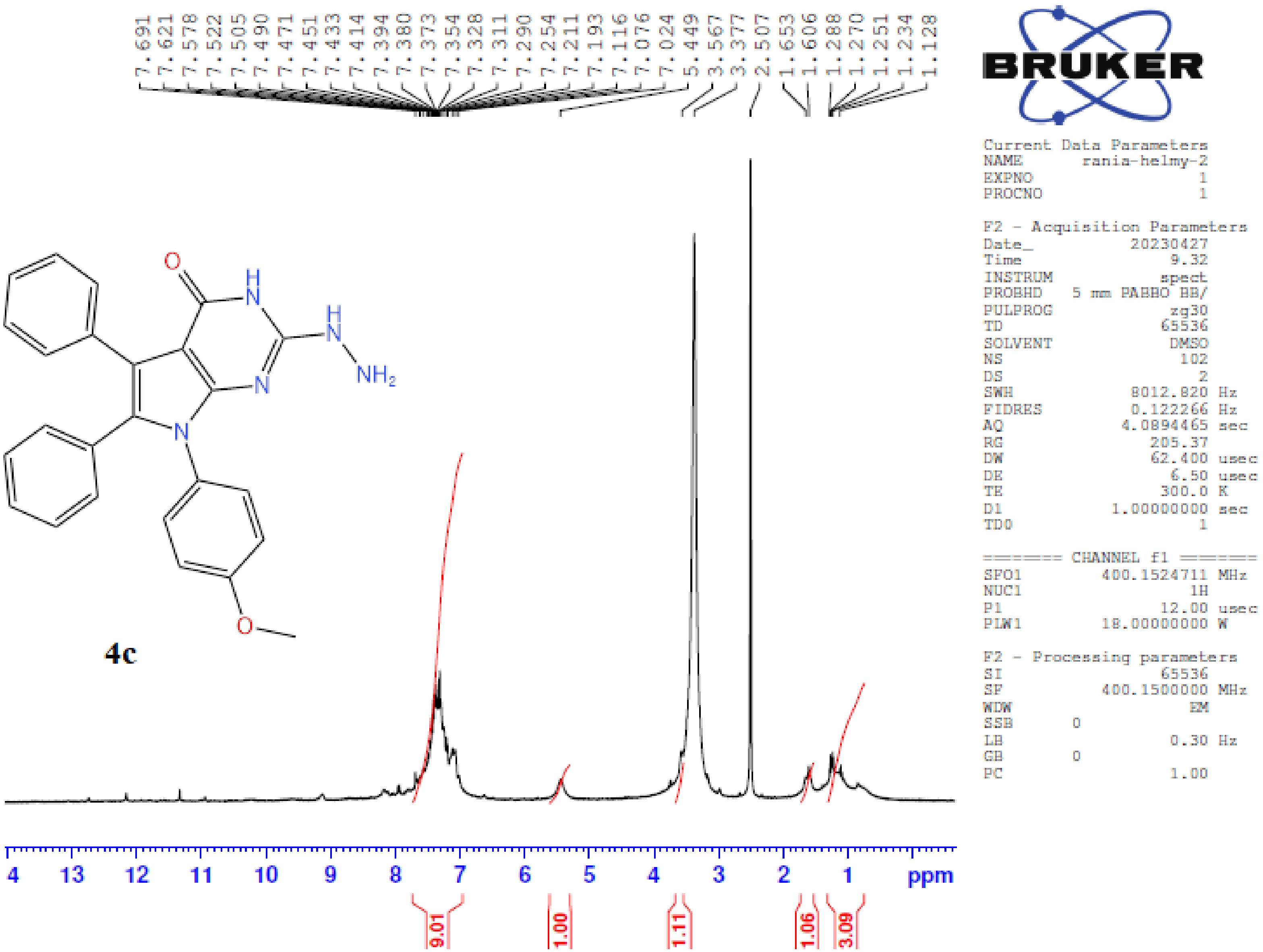
7.691 7.621 7.578 7.522 7.505 7.490 7.471 7.451 7.433 7.414 7.394 7.380 7.373 7.354 7.328 7.311 7.290 7.254 7.211 7.193 7.116 7.076 7.024 5.449 3.567 3.377 2.507 1.653 1.606 1.288 1.270 1.251 1.234 1.128
BRUKER
O
H
N
H
N
NH2
N
N
O
4c
Current Data Parameters
NAME rania-helmy-2
EXPNO 1
PROCNO 1
F2 - Acquisition Parameters
Date_ 20230427
Time 9.32
INSTRUM spect
PROBHD 5 mm PABBO BB/
PULPROG zg30
TD 65536
SOLVENT DMSO
NS 102
DS 2
SWH 8012.820 Hz
FIDRES 0.122266 Hz
AQ 4.0894465 sec
RG 205.37
DW 62.400 usec
DE 6.50 usec
TE 300.0 K
D1 1.00000000 sec
TD0 1
======= CHANNEL f1 =======
SFO1 400.1524711 MHz
NUC1 1H
P1 12.00 usec
PLW1 18.00000000 W
F2 - Processing parameters
SI 65536
SF 400.1500000 MHz
WDW EM
SSB 0
LB 0.30 Hz
GB 0
PC 1.00
4 13 12 11 10 9 8 7 6 5 4 3 2 1 ppm
9.01
1.00
1.11
1.06
3.09

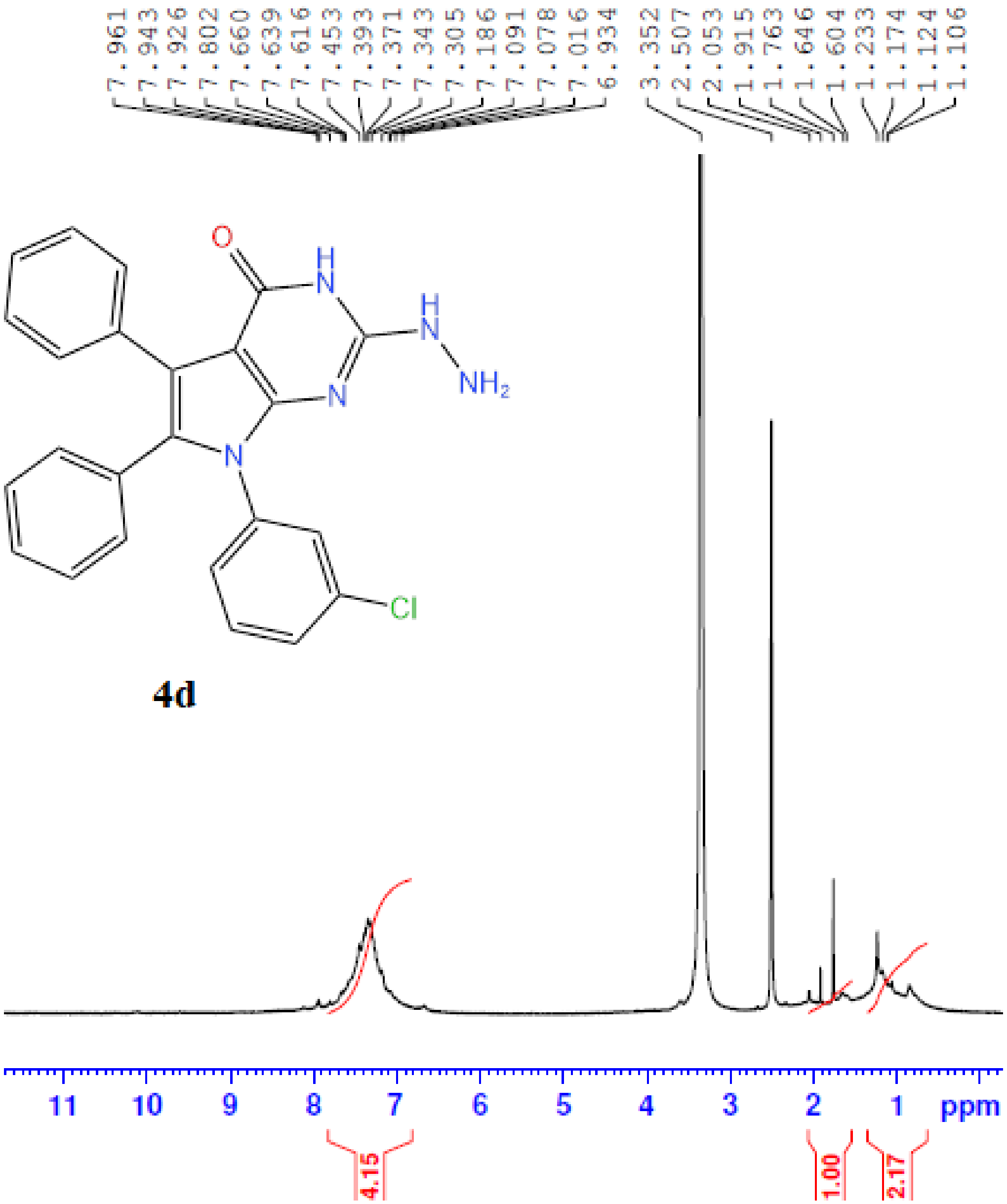

4d

BRUKER

Current Data Parameters
NAME rania-helmy-9'
EXPNO 2
PROCNO 1

F2 - Acquisition Parameters
Date_ 20230430
Time 11.54
INSTRUM spect
PROBHD 5 mm PABBO BB/
PULPROG zg30
TD 65536
SOLVENT DMSO
NS 76
DS 2
SWH 8012.820 Hz
FIDRES 0.122266 Hz
AQ 4.0894465 sec
RG 205.37
DW 62.400 usec
DE 6.50 usec
TE 299.9 K
D1 1.00000000 sec
TD0 1

======= CHANNEL f1 =======
SFO1 400.1524711 MHz
NUC1 1H
P1 12.00 usec
PLW1 18.00000000 W

F2 - Processing parameters
SI 65536
SF 400.1500000 MHz
WDW EM
SSB 0
LB 0.30 Hz
GB 0
PC 1.00

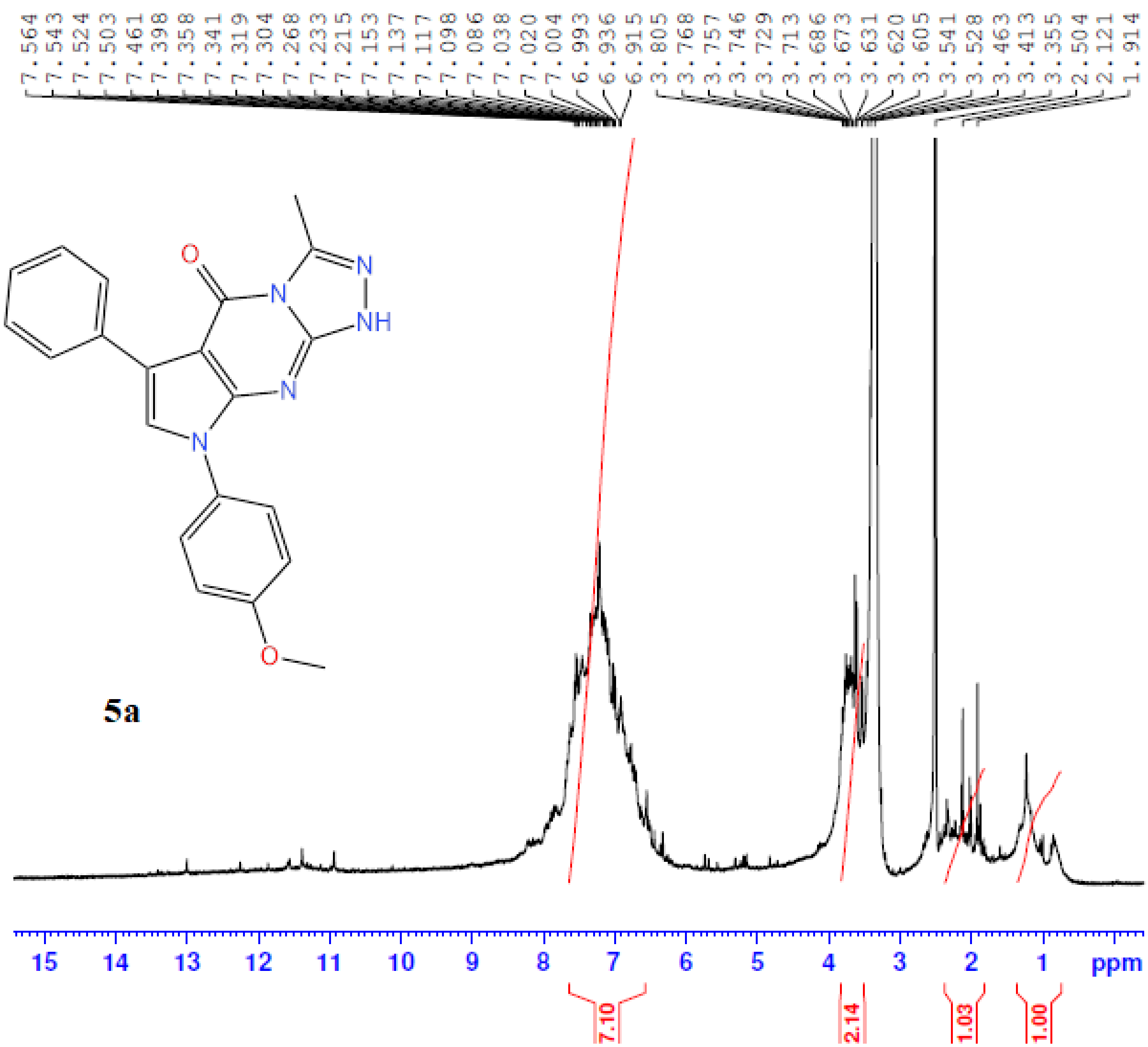

Current Data Parameters
NAME rania-helmy-19
EXPNO 1
PROCNO 1

F2 - Acquisition Parameters
Date_ 20230427
Time 12.31
INSTRUM spect
PROBHD 5 mm PABBO BB/
PULPROG zg30
TD 65536
SOLVENT DMSO
NS 91
DS 2
SWH 8012.820 Hz
FIDRES 0.122266 Hz
AQ 4.0894465 sec
RG 205.37
DW 62.400 usec
DE 6.50 usec
TE 300.0 K
D1 1.00000000 sec
TD0 1

======= CHANNEL f1 =======
SFO1 400.1524711 MHz
NUC1 1H
P1 12.00 usec
PLW1 18.00000000 W

F2 - Processing parameters
SI 65536
SF 400.1500000 MHz
WDW EM
SSB 0
LB 0.30 Hz
GB 0
PC 1.00

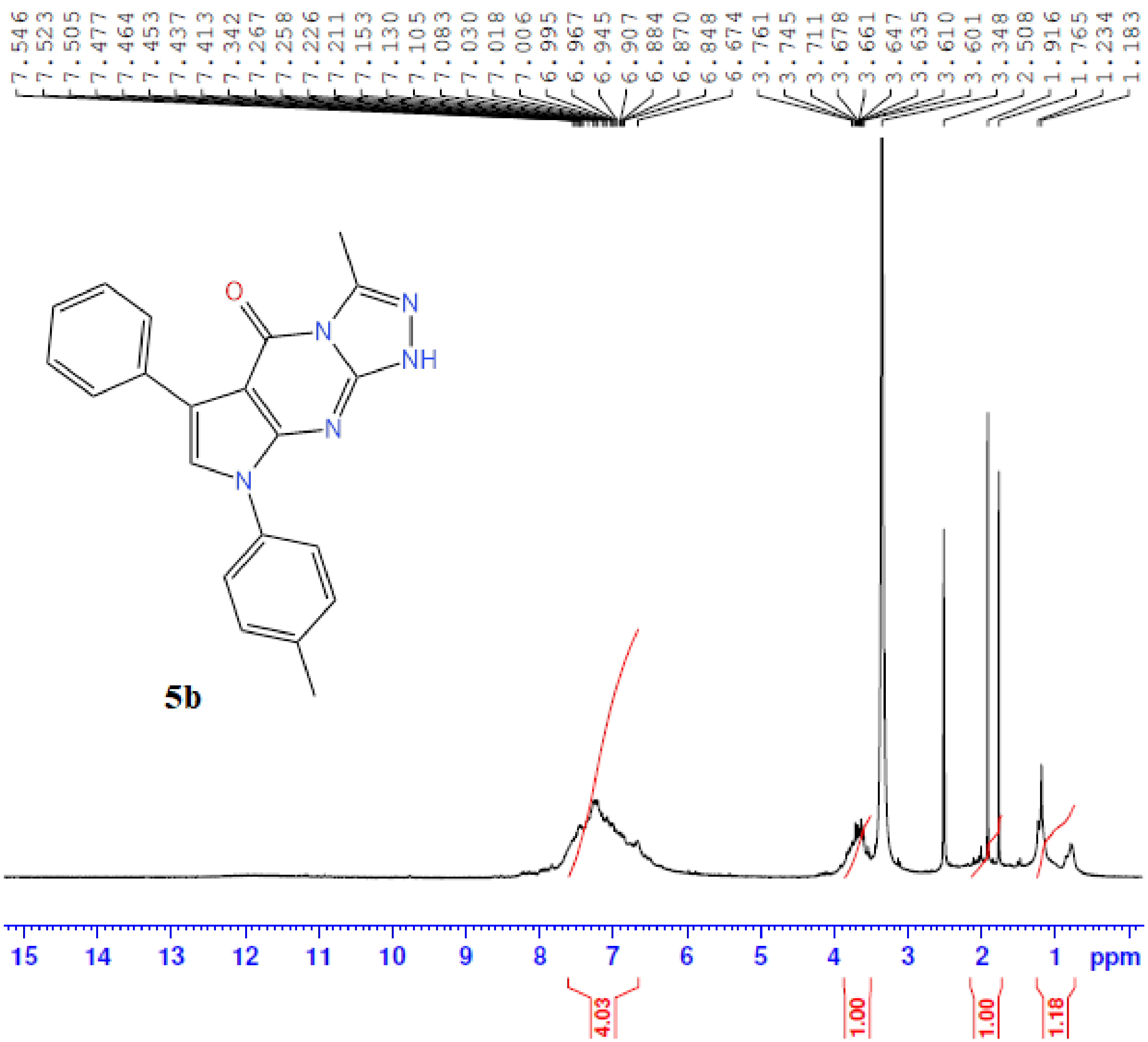

Current Data Parameters
NAME rania-helmy-4
EXPNO 2
PROCNO 1

F2 - Acquisition Parameters
Date_ 20230430
Time 11.25
INSTRUM spect
PROBHD 5 mm PABBO BB/
PULPROG zg30
TD 65536
SOLVENT DMSO
NS 98
DS 2
SWH 8012.820 Hz
FIDRES 0.122266 Hz
AQ 4.0894465 sec
RG 205.37
DW 62.400 usec
DE 6.50 usec
TE 300.0 K
D1 1.00000000 sec
TD0 1

======= CHANNEL f1 =======
SFO1 400.1524711 MHz
NUC1 1H
P1 12.00 usec
PLW1 18.00000000 W

F2 - Processing parameters
SI 65536
SF 400.1500000 MHz
WDW EM
SSB 0
LB 0.30 Hz
GB 0
PC 1.00

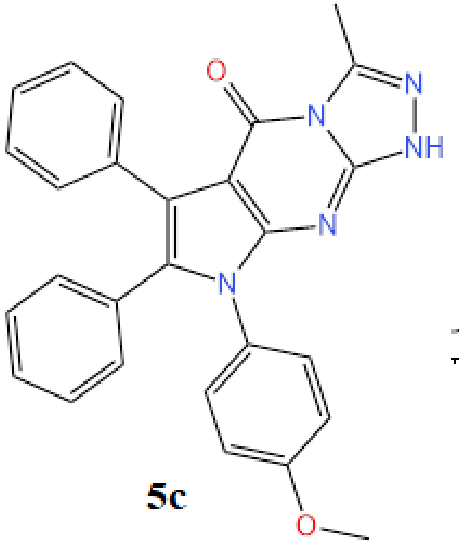

5c

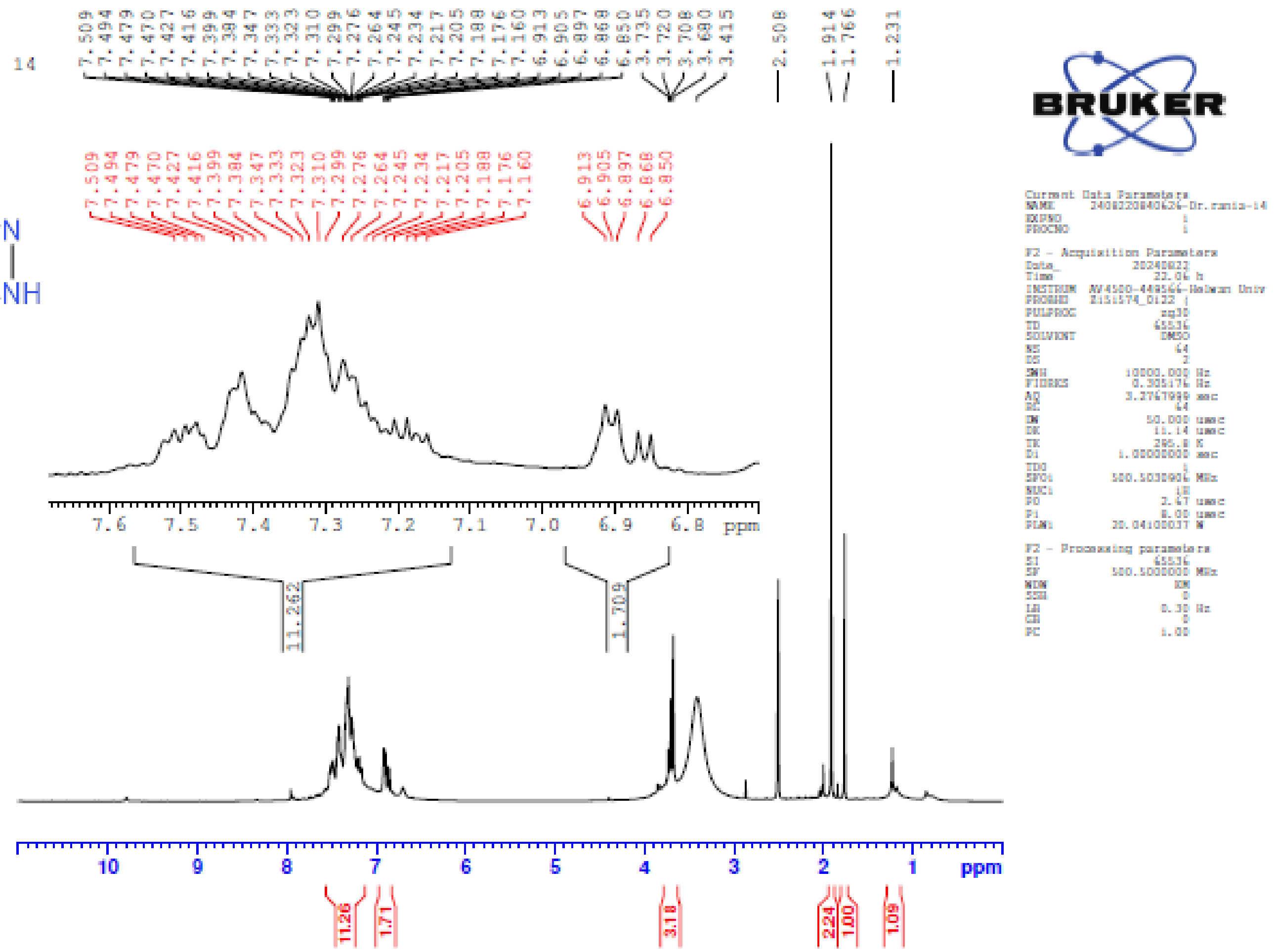

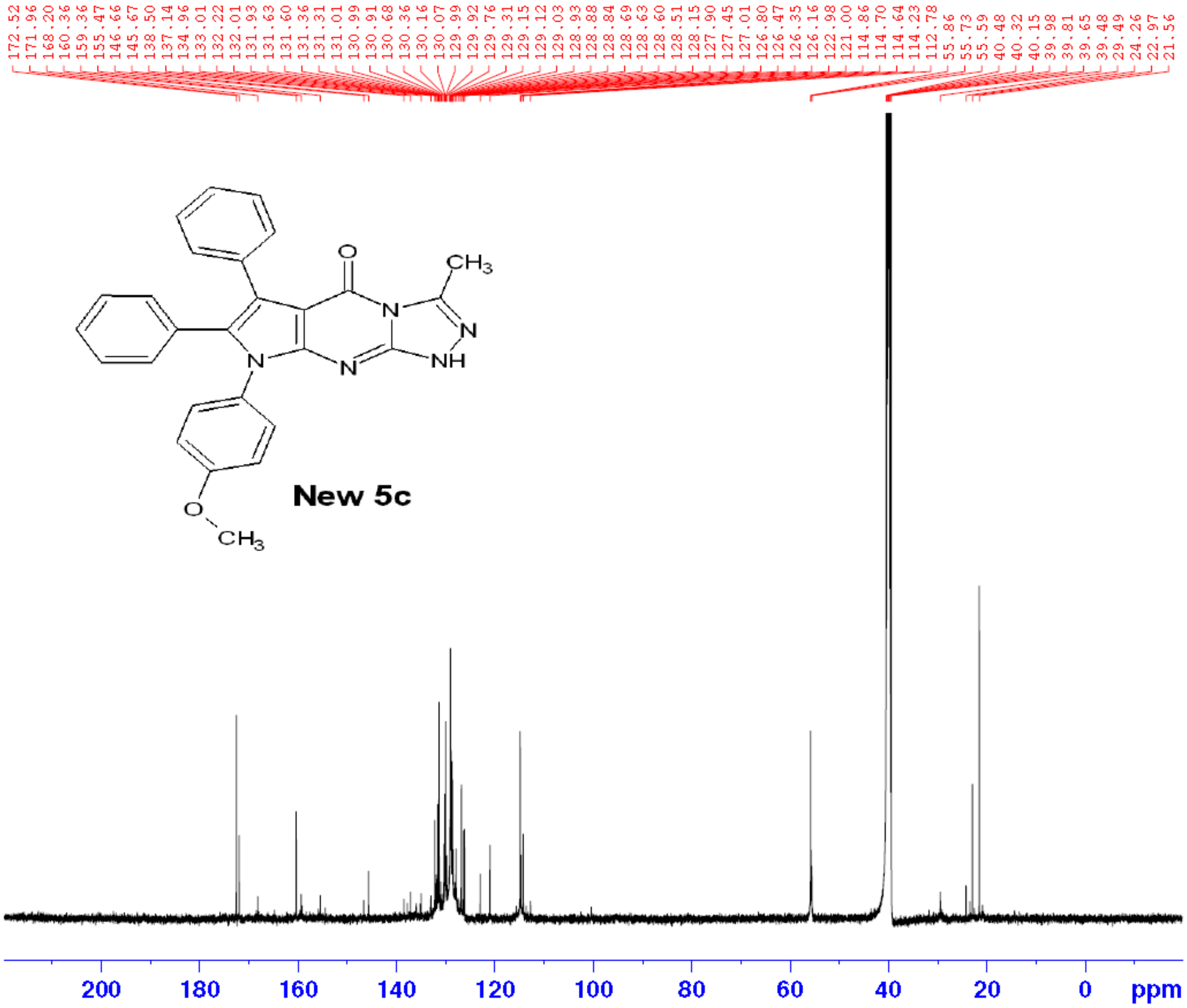

New 5c

```
Current Data Parameters
NAME      241020-035-0719-0Dr.Rania-1
EXPNO                     1
PROCNO                    1

F2 - Acquisition Parameters
Date_              20241021
Time                   2.47 h
INSTRUM  AV4500-449566-Helwan Univ
PROBHD   Z151574_0122 (
PULPROG             zgpg30
TD                   65536
SOLVENT               DMSO
NS                    4096
DS                       4
SWH              30120.482 Hz
FIDRES            0.919204 Hz
AQ               1.0878977 sec
RG                     101
DW                  16.600 usec
DE                    6.50 usec
TE                   298.4 K
D1              2.00000000 sec
D11             0.03000000 sec
TD0                      1
SFO1           125.8634102 MHz
NUC1                   13C
P0                    3.00 usec
P1                    9.00 usec
PLW1           96.27300262 W
SFO2           500.5020020 MHz
NUC2                    1H
CPDPRG[2           waltz65
PCPD2                80.00 usec
PLW2           20.04100037 W
PLW12           0.20040999 W
PLW13           0.10081000 W

F2 - Processing parameters
SI                   32768
SF             125.8508251 MHz
WDW                     EM
SSB                      0
LB                    1.00 Hz
GB                       0
PC                    1.40
```

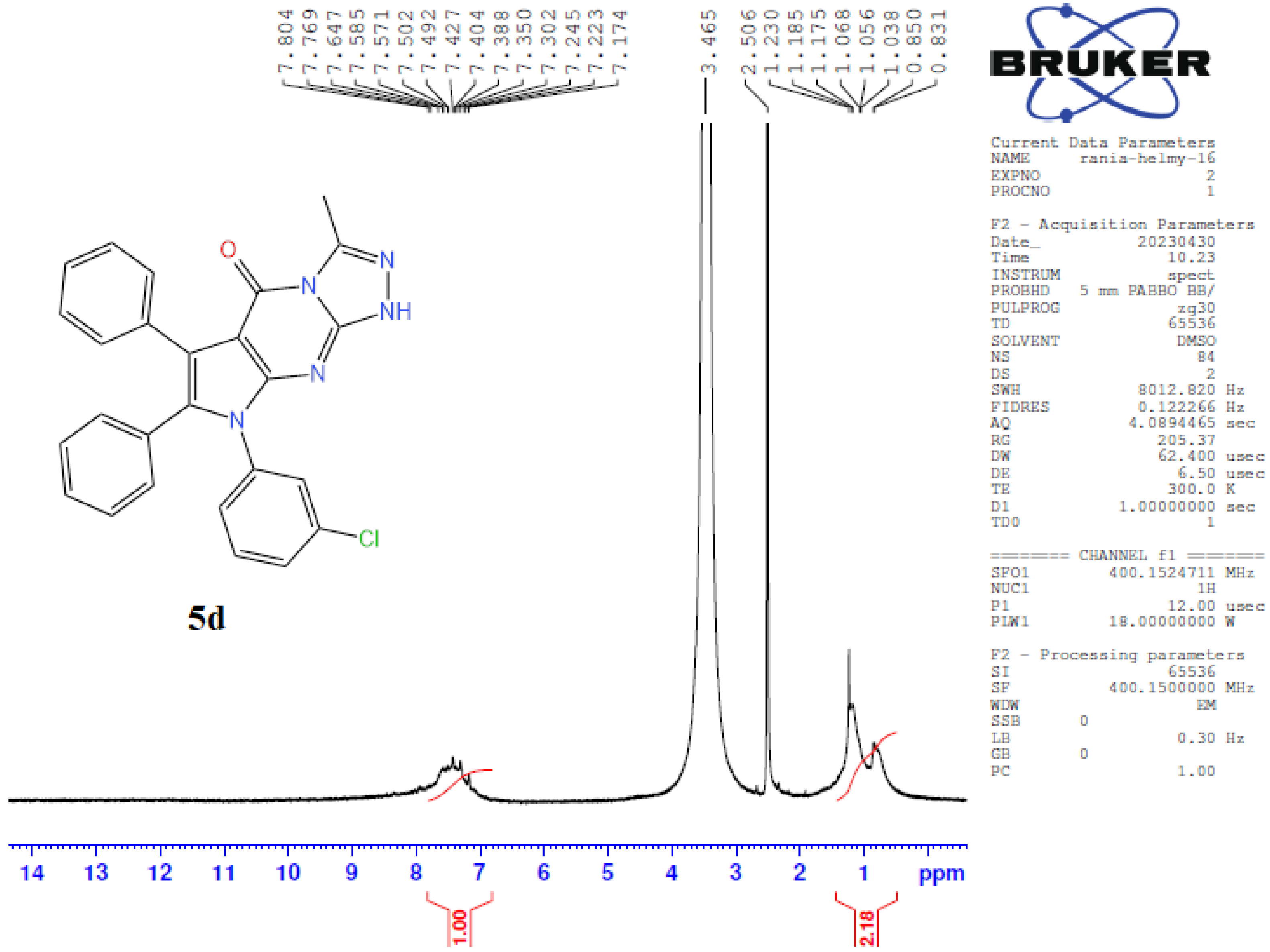

7.804 7.769 7.647 7.585 7.571 7.502 7.492 7.427 7.404 7.388 7.350 7.302 7.245 7.223 7.174
3.465
2.506 1.230 1.185 1.175 1.068 1.056 1.038 0.850 0.831
O
N
N
NH
N
N
Cl
5d
14 13 12 11 10 9 8 7 6 5 4 3 2 1 ppm
1.00
2.18
BRUKER
Current Data Parameters
NAME rania-helmy-16
EXPNO 2
PROCNO 1
F2 - Acquisition Parameters
Date_ 20230430
Time 10.23
INSTRUM spect
PROBHD 5 mm PABBO BB/
PULPROG zg30
TD 65536
SOLVENT DMSO
NS 84
DS 2
SWH 8012.820 Hz
FIDRES 0.122266 Hz
AQ 4.0894465 sec
RG 205.37
DW 62.400 usec
DE 6.50 usec
TE 300.0 K
D1 1.00000000 sec
TD0 1
======= CHANNEL f1 =======
SFO1 400.1524711 MHz
NUC1 1H
P1 12.00 usec
PLW1 18.00000000 W
F2 - Processing parameters
SI 65536
SF 400.1500000 MHz
WDW EM
SSB 0
LB 0.30 Hz
GB 0
PC 1.00

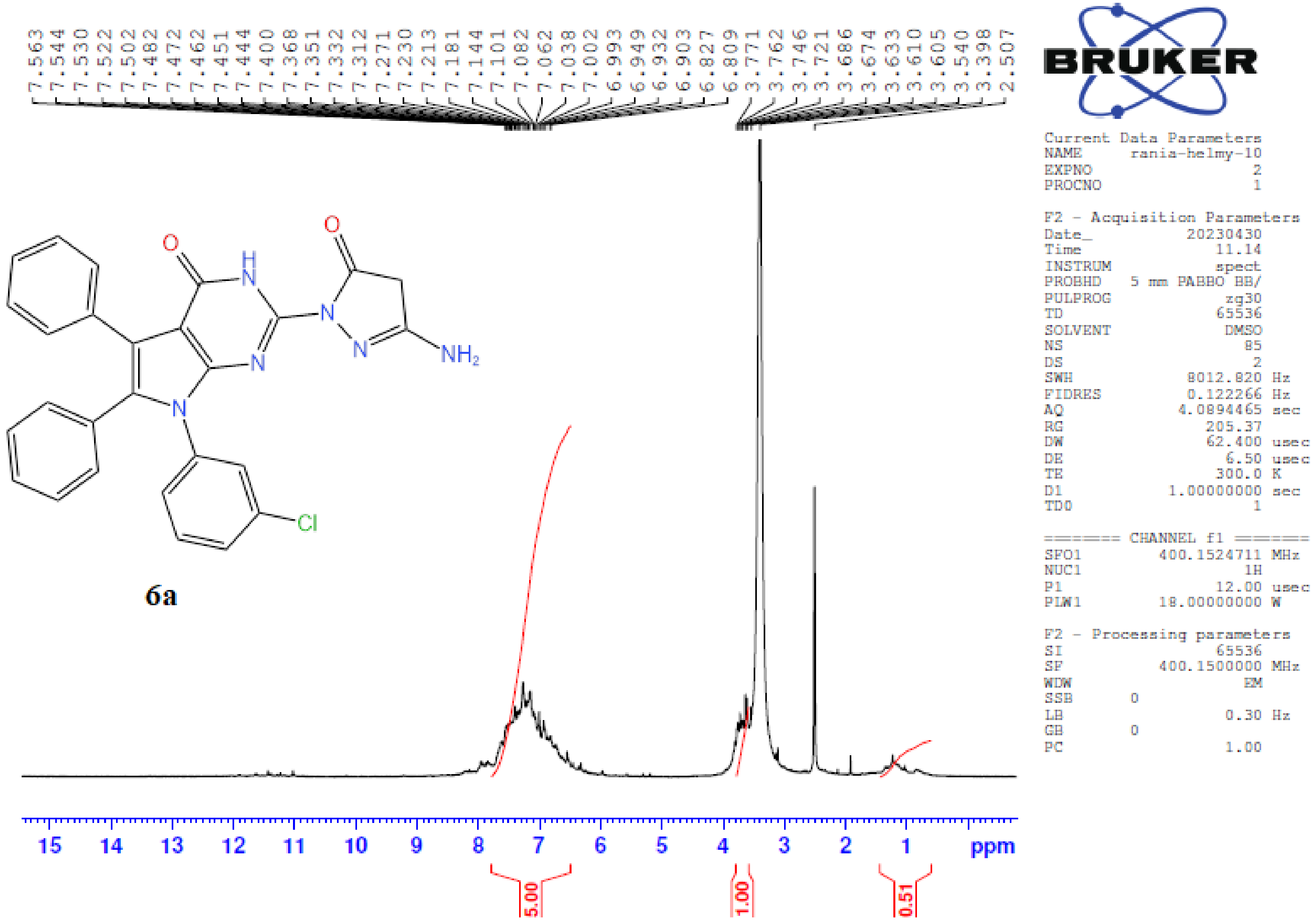
7.563 7.544 7.530 7.522 7.502 7.482 7.472 7.462 7.451 7.444 7.400 7.368 7.351 7.332 7.312 7.271 7.230 7.213 7.181 7.144 7.101 7.082 7.062 7.038 7.002 6.993 6.949 6.932 6.903 6.827 6.809 3.771 3.762 3.746 3.721 3.686 3.674 3.633 3.610 3.605 3.540 3.398 2.507
BRUKER
O
H
N
O
N
N
N
NH2
N
Cl
6a
Current Data Parameters
NAME rania-helmy-10
EXPNO 2
PROCNO 1
F2 - Acquisition Parameters
Date_ 20230430
Time 11.14
INSTRUM spect
PROBHD 5 mm PABBO BB/
PULPROG zg30
TD 65536
SOLVENT DMSO
NS 85
DS 2
SWH 8012.820 Hz
FIDRES 0.122266 Hz
AQ 4.0894465 sec
RG 205.37
DW 62.400 usec
DE 6.50 usec
TE 300.0 K
D1 1.00000000 sec
TD0 1
======== CHANNEL f1 ========
SFO1 400.1524711 MHz
NUC1 1H
P1 12.00 usec
PLW1 18.00000000 W
F2 - Processing parameters
SI 65536
SF 400.1500000 MHz
WDW EM
SSB 0
LB 0.30 Hz
GB 0
PC 1.00
15 14 13 12 11 10 9 8 7 6 5 4 3 2 1 ppm
5.00
1.00
0.51

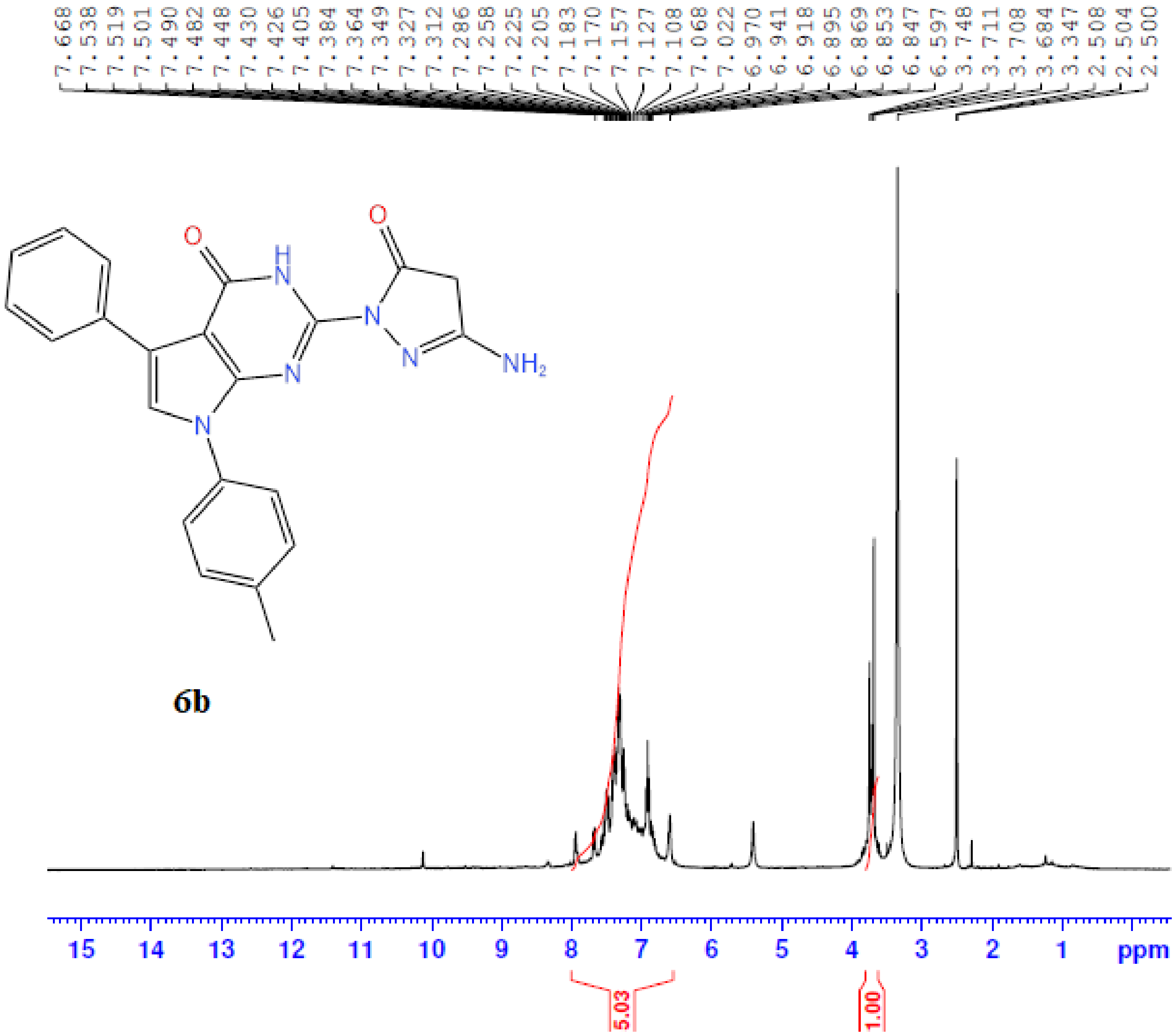

6b

Current Data Parameters
NAME rania-helmy-11
EXPNO 2
PROCNO 1

F2 - Acquisition Parameters
Date_ 20230427
Time 11.32
INSTRUM spect
PROBHD 5 mm PABBO BB/
PULPROG zg30
TD 65536
SOLVENT DMSO
NS 97
DS 2
SWH 8012.820 Hz
FIDRES 0.122266 Hz
AQ 4.0894465 sec
RG 205.37
DW 62.400 usec
DE 6.50 usec
TE 300.0 K
D1 1.00000000 sec
TD0 1

======= CHANNEL f1 =======
SFO1 400.1524711 MHz
NUC1 1H
P1 12.00 usec
PLW1 18.00000000 W

F2 - Processing parameters
SI 65536
SF 400.1500000 MHz
WDW EM
SSB 0
LB 0.30 Hz
GB 0
PC 1.00

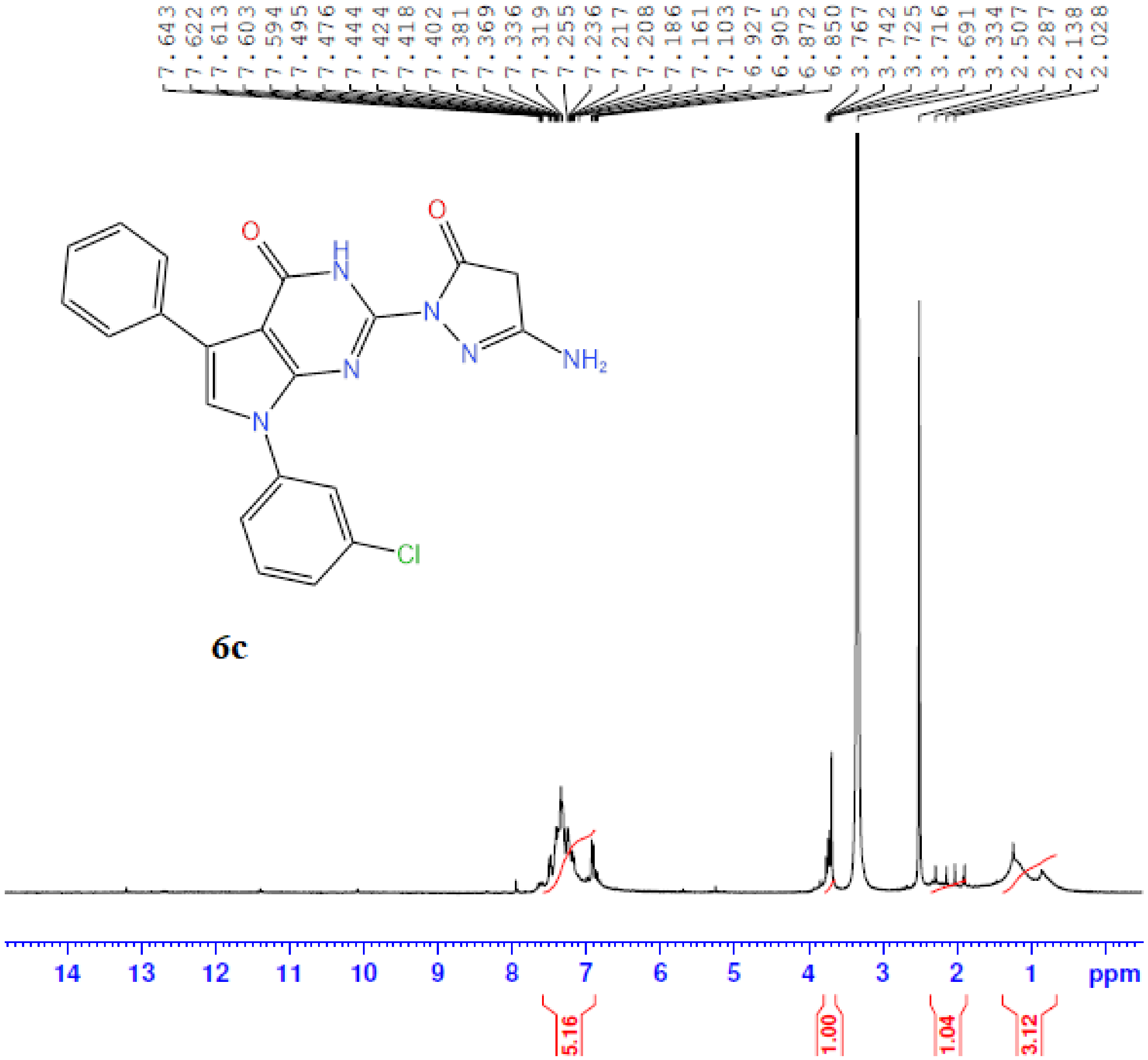
7.643
7.622
7.613
7.603
7.594
7.495
7.476
7.444
7.424
7.418
7.402
7.381
7.369
7.336
7.319
7.255
7.236
7.217
7.208
7.186
7.161
7.103
6.927
6.905
6.872
6.850
3.767
3.742
3.725
3.716
3.691
3.334
2.507
2.287
2.138
2.028
14 13 12 11 10 9 8 7 6 5 4 3 2 1 ppm
5.16
1.00
1.04
3.12
BRUKER
Current Data Parameters
NAME rania-helmy-5
EXPNO 1
PROCNO 1
F2 - Acquisition Parameters
Date_ 20230427
Time 9.57
INSTRUM spect
PROBHD 5 mm PABBO BB/
PULPROG zg30
TD 65536
SOLVENT DMSO
NS 102
DS 2
SWH 8012.820 Hz
FIDRES 0.122266 Hz
AQ 4.0894465 sec
RG 205.37
DW 62.400 usec
DE 6.50 usec
TE 300.0 K
D1 1.00000000 sec
TD0 1
======= CHANNEL f1 =======
SFO1 400.1524711 MHz
NUC1 1H
P1 12.00 usec
PLW1 18.00000000 W
F2 - Processing parameters
SI 65536
SF 400.1500000 MHz
WDW EM
SSB 0
LB 0.30 Hz
GB 0
PC 1.00
O
H
N
O
N
N
N
NH2
N
Cl

6c

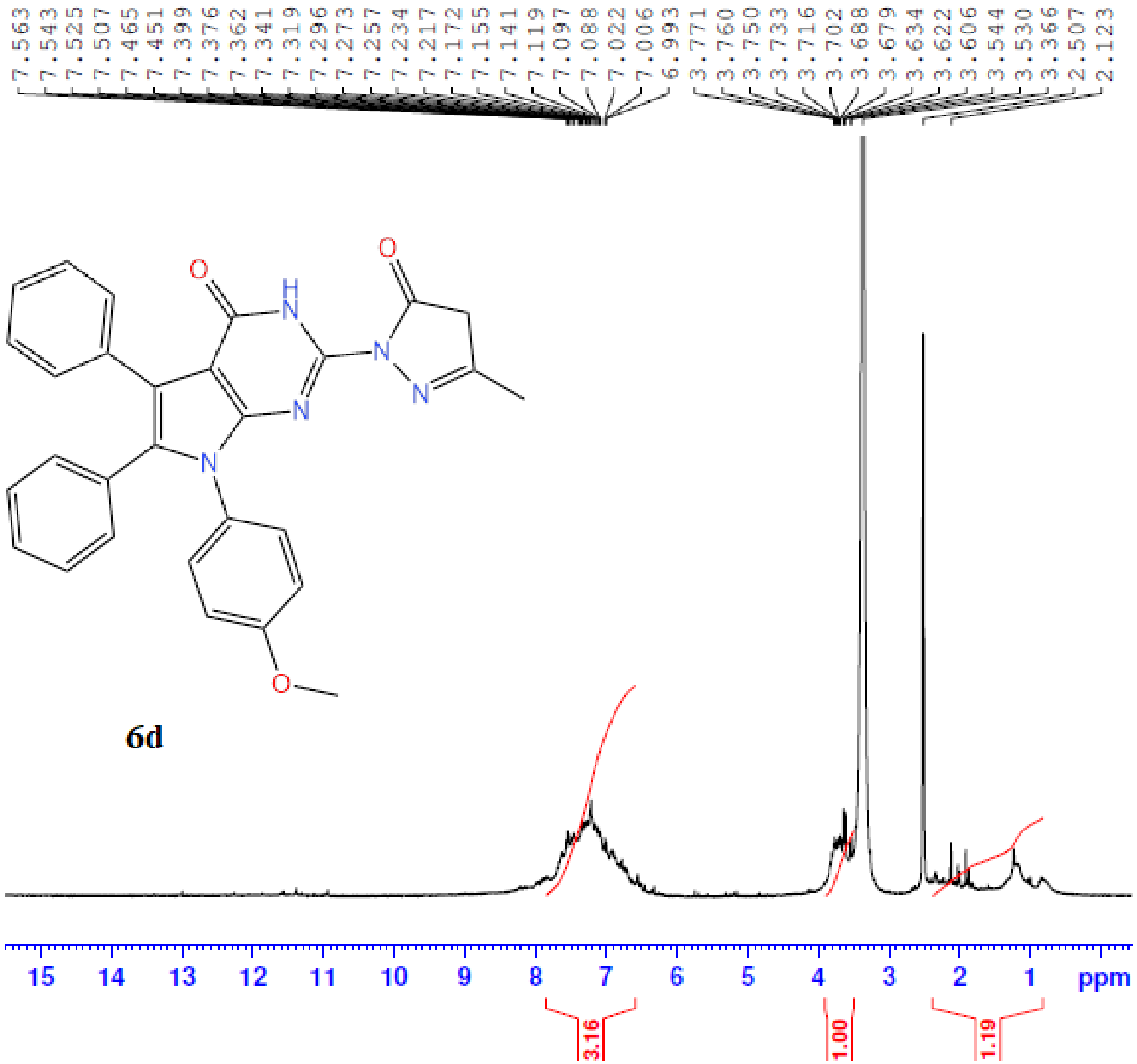


```
Current Data Parameters
NAME         rania-helmy-9
EXPNO                    2
PROCNO                   1

F2 - Acquisition Parameters
Date_             20230430
Time                 11.45
INSTRUM              spect
PROBHD   5 mm PABBO BB/
PULPROG               zg30
TD                   65536
SOLVENT               DMSO
NS                      75
DS                       2
SWH              8012.820 Hz
FIDRES           0.122266 Hz
AQ              4.0894465 sec
RG                  205.37
DW                  62.400 usec
DE                    6.50 usec
TE                   300.0 K
D1             1.00000000 sec
TD0                      1

======= CHANNEL f1 =======
SFO1         400.1524711 MHz
NUC1                    1H
P1                   12.00 usec
PLW1           18.00000000 W

F2 - Processing parameters
SI                   65536
SF           400.1500000 MHz
WDW                     EM
SSB      0
LB                    0.30 Hz
GB       0
PC                    1.00
```

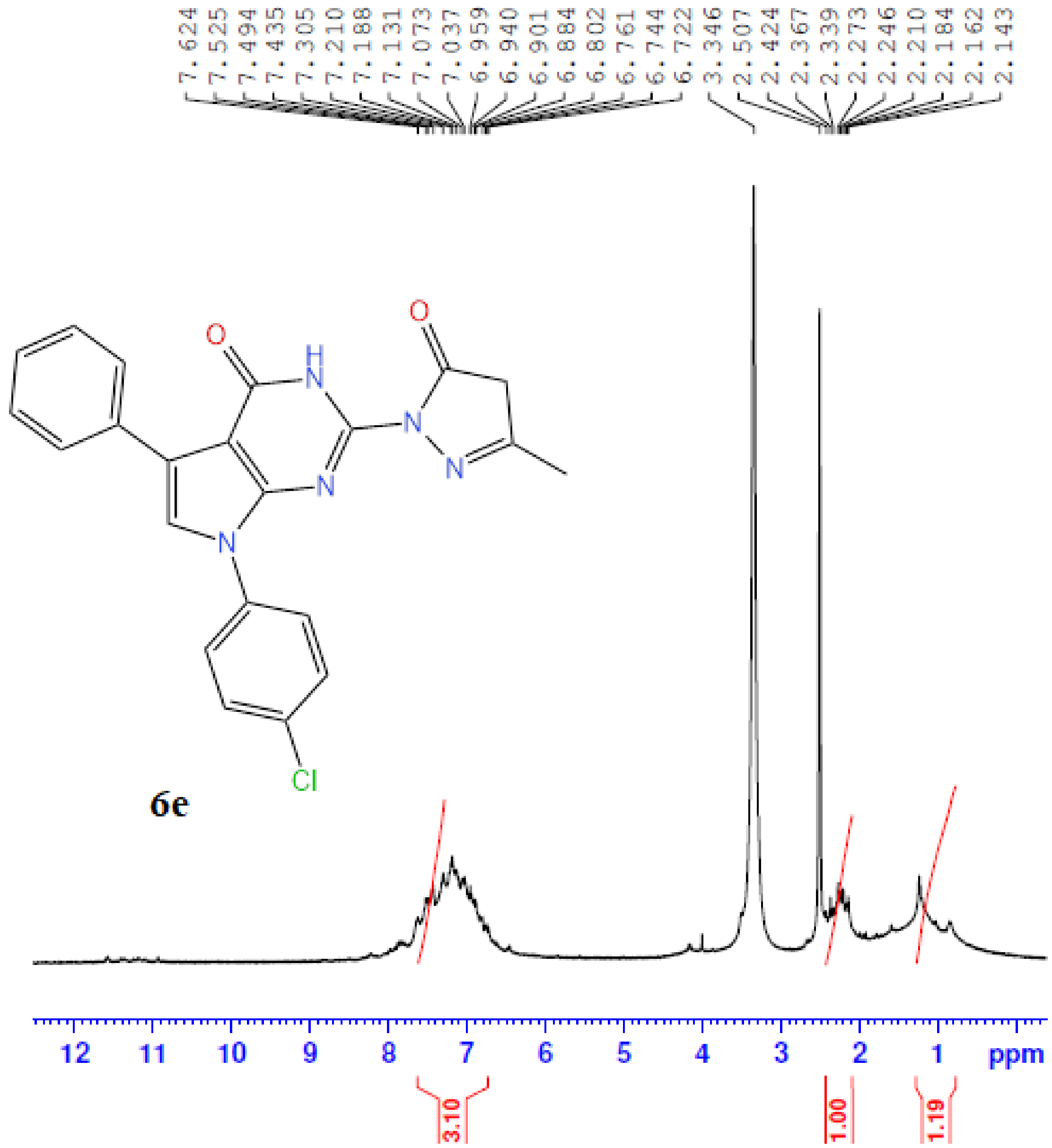


```
Current Data Parameters
NAME          rania-helmy-7
EXPNO                     1
PROCNO                    1

F2 - Acquisition Parameters
Date_              20230427
Time                  10.23
INSTRUM               spect
PROBHD   5 mm PABBO BB/
PULPROG                zg30
TD                    65536
SOLVENT                DMSO
NS                      102
DS                        2
SWH            8012.820 Hz
FIDRES         0.122266 Hz
AQ           4.0894465 sec
RG                   205.37
DW             62.400 usec
DE               6.50 usec
TE                300.0 K
D1          1.00000000 sec
TD0                       1

======= CHANNEL f1 =======
SFO1        400.1524711 MHz
NUC1                     1H
P1              12.00 usec
PLW1        18.00000000 W

F2 - Processing parameters
SI                    65536
SF          400.1500000 MHz
WDW                      EM
SSB       0
LB                  0.30 Hz
GB        0
PC                     1.00
```

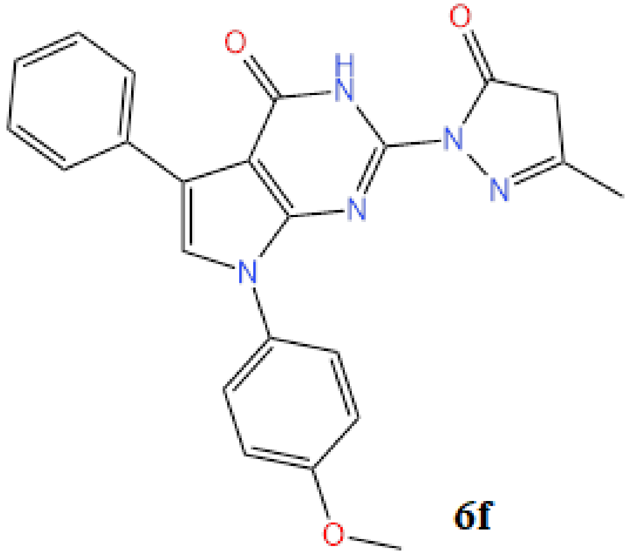

6f

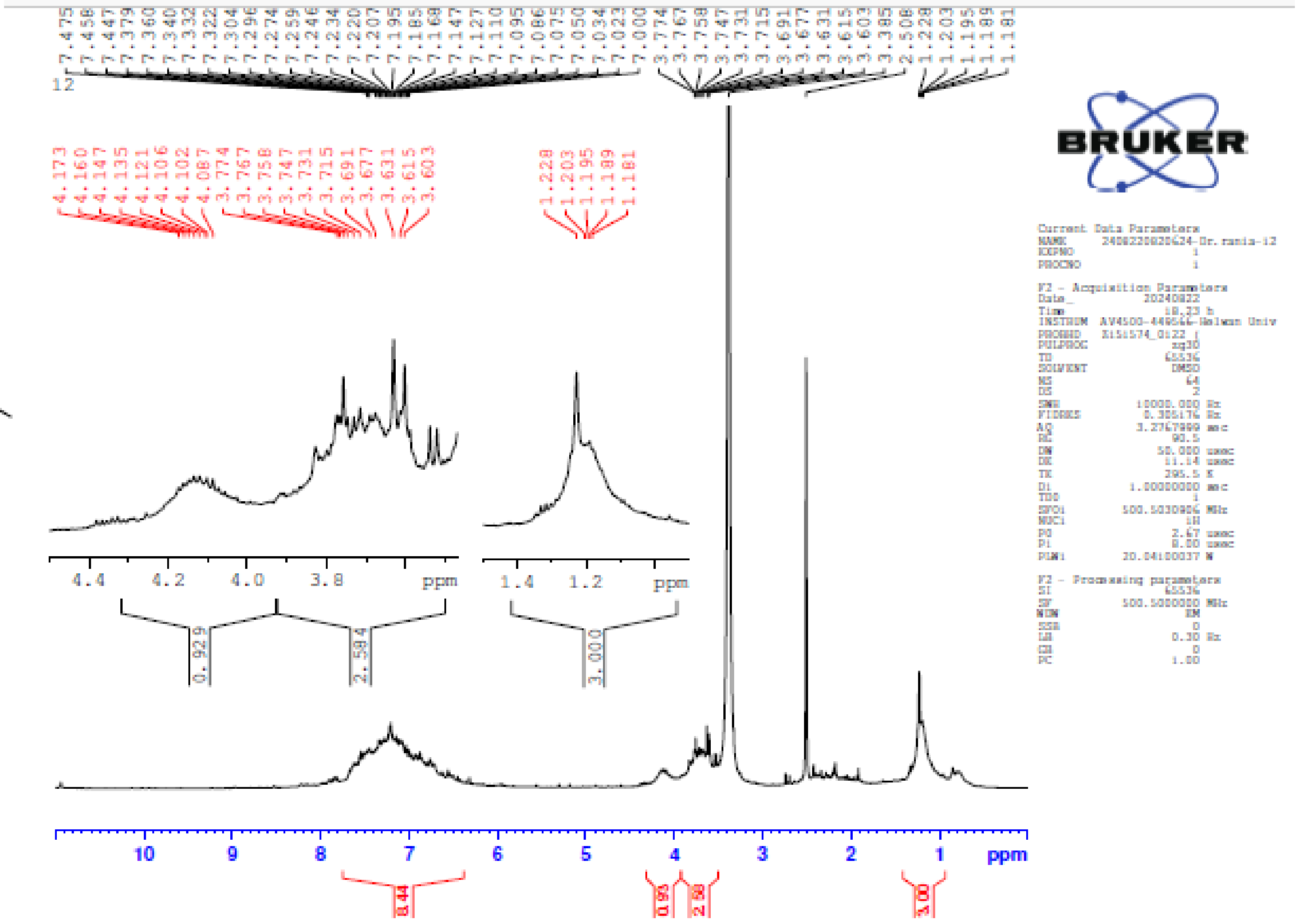
BRUKER
Current Data Parameters
F2 - Acquisition Parameters
F2 - Processing parameters
ppm

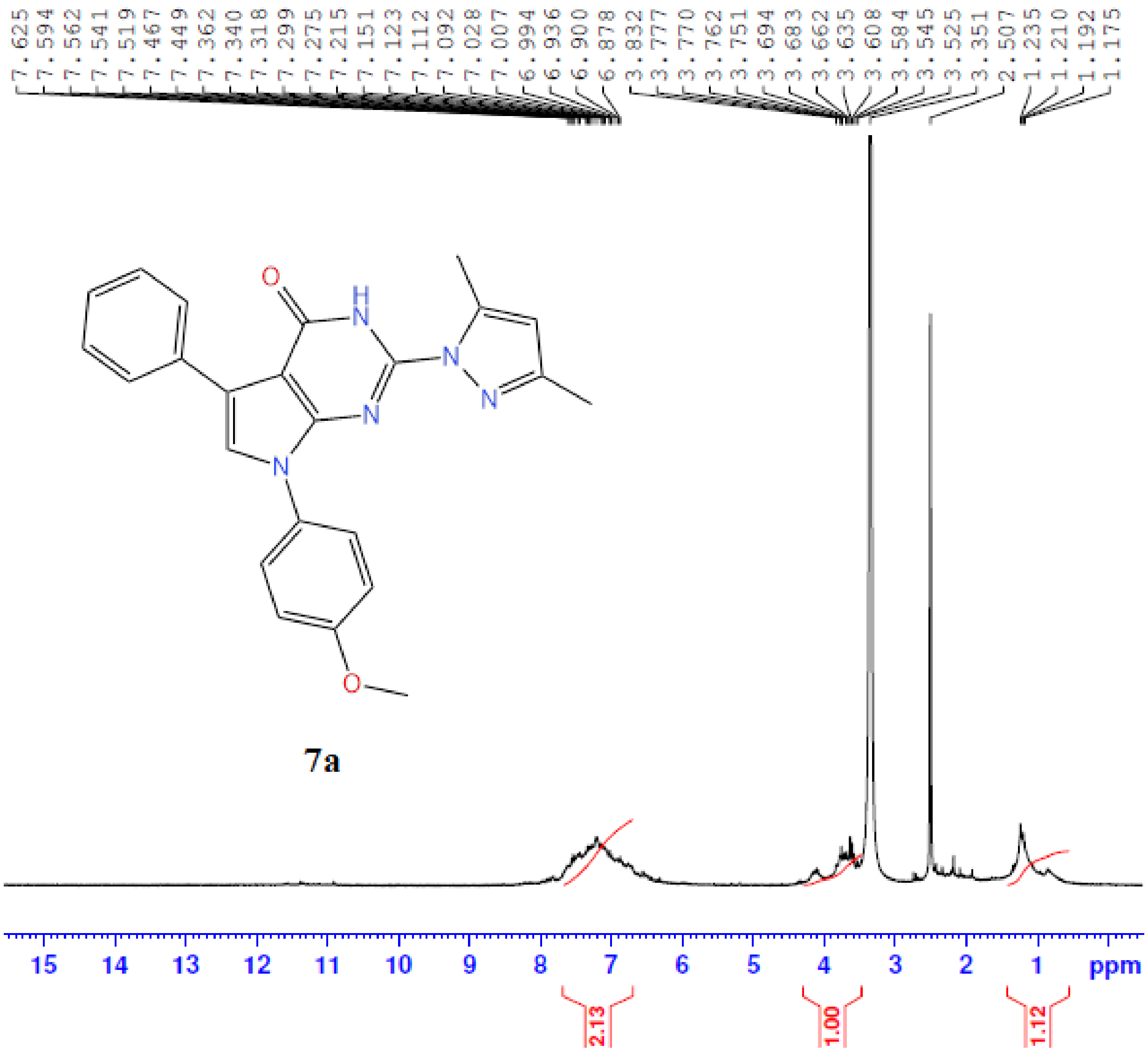

Current Data Parameters
NAME rania-helmy-12
EXPNO 2
PROCNO 1

F2 - Acquisition Parameters
Date_ 20230430
Time 11.04
INSTRUM spect
PROBHD 5 mm PABBO BB/
PULPROG zg30
TD 65536
SOLVENT DMSO
NS 91
DS 2
SWH 8012.820 Hz
FIDRES 0.122266 Hz
AQ 4.0894465 sec
RG 205.37
DW 62.400 usec
DE 6.50 usec
TE 300.0 K
D1 1.00000000 sec
TD0 1

======= CHANNEL f1 =======
SFO1 400.1524711 MHz
NUC1 1H
P1 12.00 usec
PLW1 18.00000000 W

F2 - Processing parameters
SI 65536
SF 400.1500000 MHz
WDW EM
SSB 0
LB 0.30 Hz
GB 0
PC 1.00

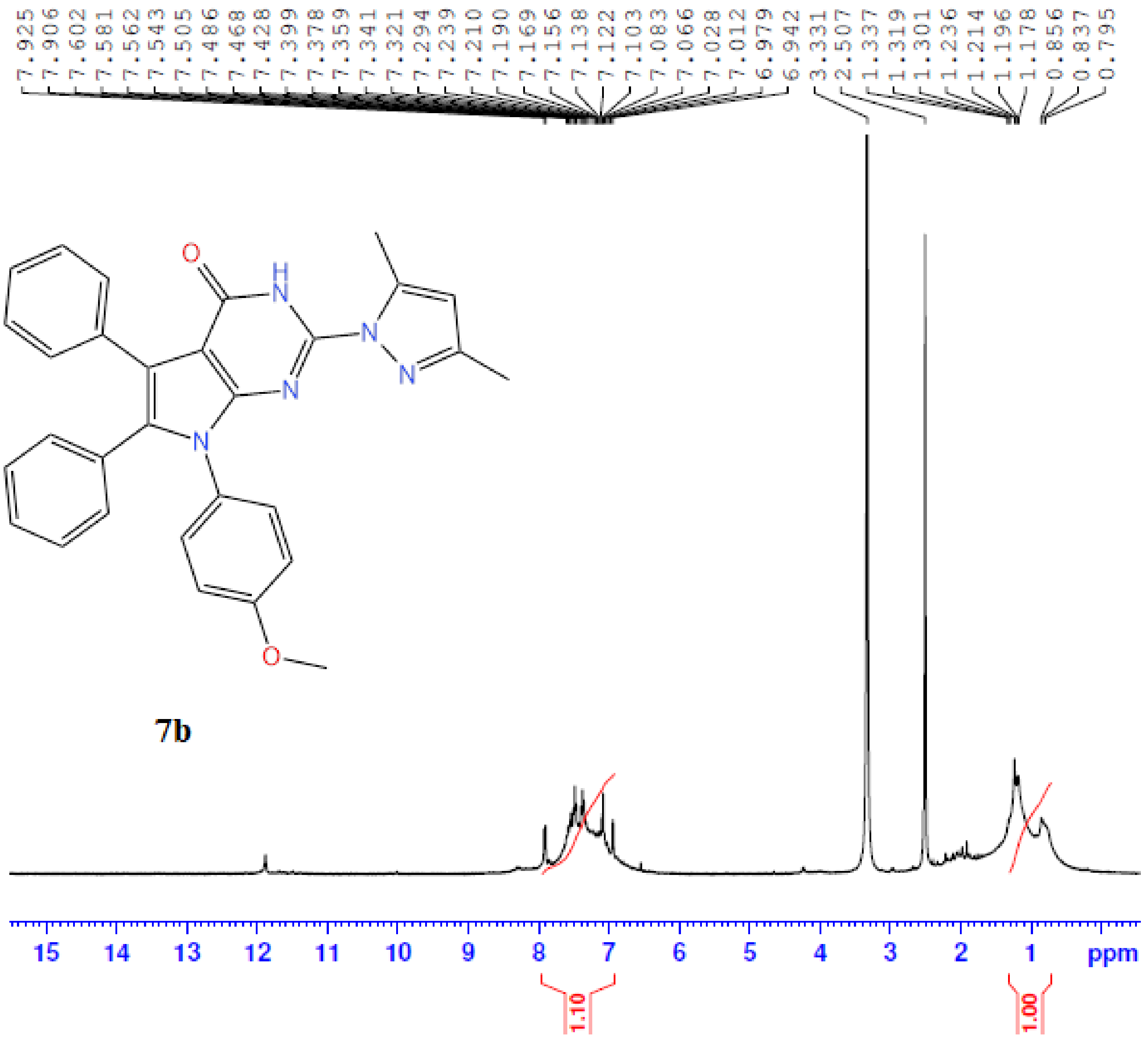

Current Data Parameters
NAME rania-helmy-8
EXPNO 1
PROCNO 1

F2 - Acquisition Parameters
Date_ 20230427
Time 10.37
INSTRUM spect
PROBHD 5 mm PABBO BB/
PULPROG zg30
TD 65536
SOLVENT DMSO
NS 128
DS 2
SWH 8012.820 Hz
FIDRES 0.122266 Hz
AQ 4.0894465 sec
RG 205.37
DW 62.400 usec
DE 6.50 usec
TE 300.0 K
D1 1.00000000 sec
TD0 1

======= CHANNEL f1 =======
SFO1 400.1524711 MHz
NUC1 1H
P1 12.00 usec
PLW1 18.00000000 W

F2 - Processing parameters
SI 65536
SF 400.1500000 MHz
WDW EM
SSB 0
LB 0.30 Hz
GB 0
PC 1.00

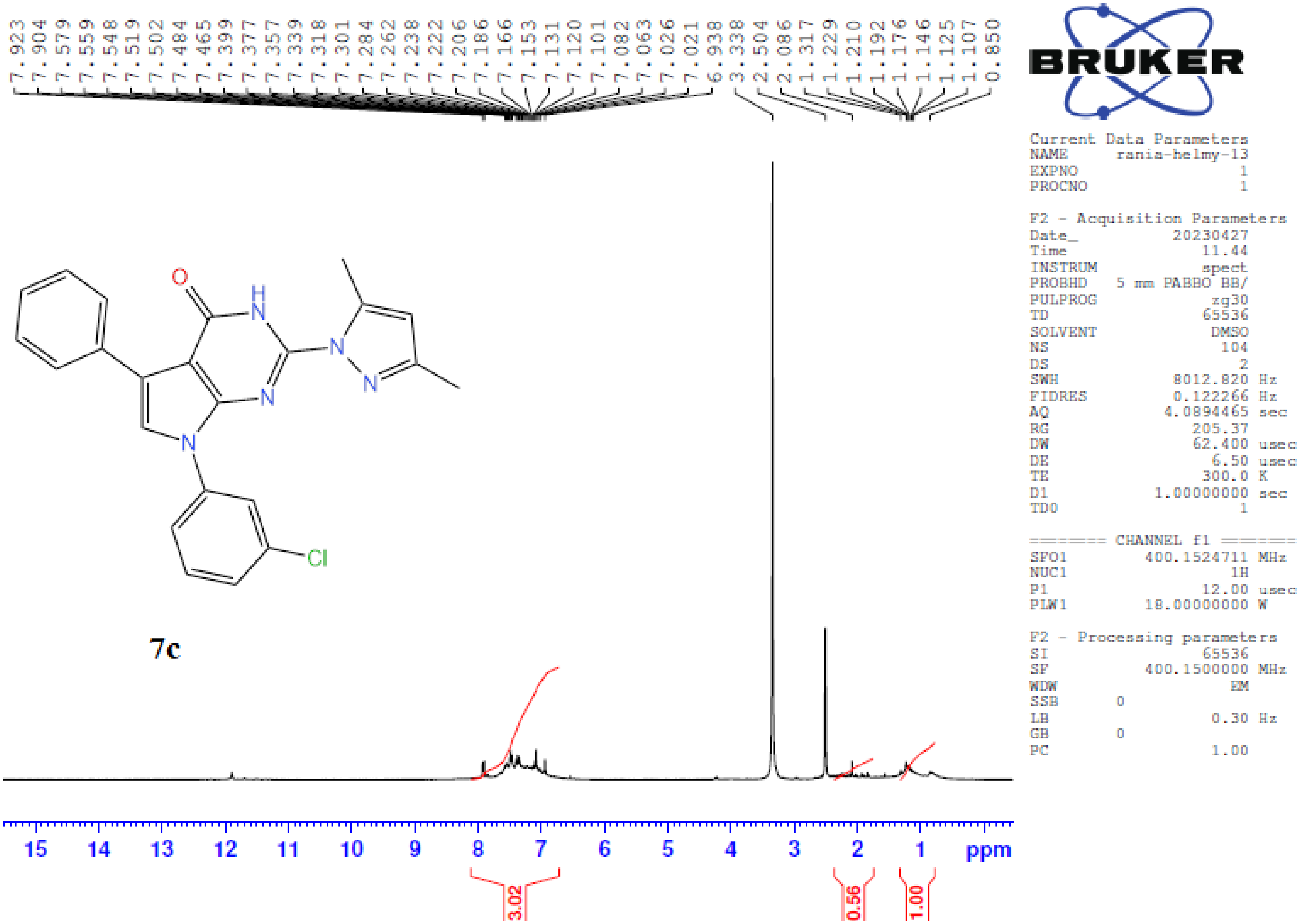
7.923 7.904 7.579 7.559 7.548 7.519 7.502 7.484 7.465 7.399 7.377 7.357 7.339 7.318 7.301 7.284 7.262 7.238 7.222 7.206 7.186 7.166 7.153 7.131 7.120 7.101 7.082 7.063 7.026 7.021 6.938 3.338 2.504 2.086 1.317 1.229 1.210 1.192 1.176 1.146 1.125 1.107 0.850
BRUKER
Current Data Parameters
NAME rania-helmy-13
EXPNO 1
PROCNO 1
F2 - Acquisition Parameters
Date_ 20230427
Time 11.44
INSTRUM spect
PROBHD 5 mm PABBO BB/
PULPROG zg30
TD 65536
SOLVENT DMSO
NS 104
DS 2
SWH 8012.820 Hz
FIDRES 0.122266 Hz
AQ 4.0894465 sec
RG 205.37
DW 62.400 usec
DE 6.50 usec
TE 300.0 K
D1 1.00000000 sec
TD0 1
======= CHANNEL f1 =======
SFO1 400.1524711 MHz
NUC1 1H
P1 12.00 usec
PLW1 18.00000000 W
F2 - Processing parameters
SI 65536
SF 400.1500000 MHz
WDW EM
SSB 0
LB 0.30 Hz
GB 0
PC 1.00
15 14 13 12 11 10 9 8 7 6 5 4 3 2 1 ppm
3.02
0.56
1.00
O
H
N
N
N
N
N
Cl

7c

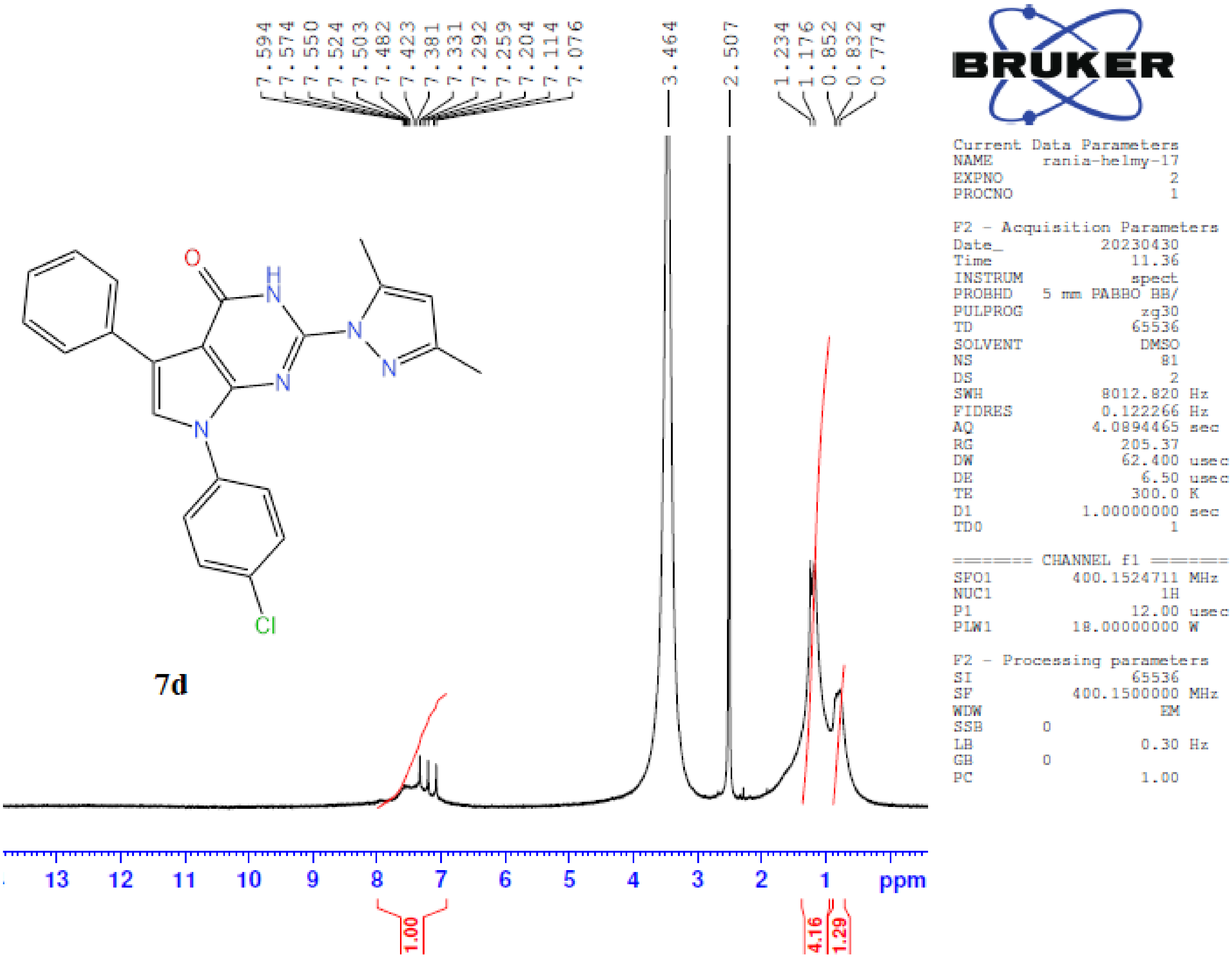
7.594
7.574
7.550
7.524
7.503
7.482
7.423
7.381
7.331
7.292
7.259
7.204
7.114
7.076
3.464
2.507
1.234
1.176
0.852
0.832
0.774
O
H
N
N
N
N
N
Cl
13 12 11 10 9 8 7 6 5 4 3 2 1 ppm
1.00
4.16
1.29
BRUKER
Current Data Parameters
NAME rania-helmy-17
EXPNO 2
PROCNO 1
F2 - Acquisition Parameters
Date_ 20230430
Time 11.36
INSTRUM spect
PROBHD 5 mm PABBO BB/
PULPROG zg30
TD 65536
SOLVENT DMSO
NS 81
DS 2
SWH 8012.820 Hz
FIDRES 0.122266 Hz
AQ 4.0894465 sec
RG 205.37
DW 62.400 usec
DE 6.50 usec
TE 300.0 K
D1 1.00000000 sec
TD0 1
======= CHANNEL f1 =======
SFO1 400.1524711 MHz
NUC1 1H
P1 12.00 usec
PLW1 18.00000000 W
F2 - Processing parameters
SI 65536
SF 400.1500000 MHz
WDW EM
SSB 0
LB 0.30 Hz
GB 0
PC 1.00

7d

HRMS Revised Results

Center for DRUG DISCOVERY RESEARCH and DEVELOPMENT
Openlynx Report -

Sample: 2097 Vial:1:B,3
File:10 Date:27-Aug-2024
Description:10

Printed: Tue Aug 27 11:55:24 2024

Peak ID	Time
11	7.65

11:(Time: 7.65)

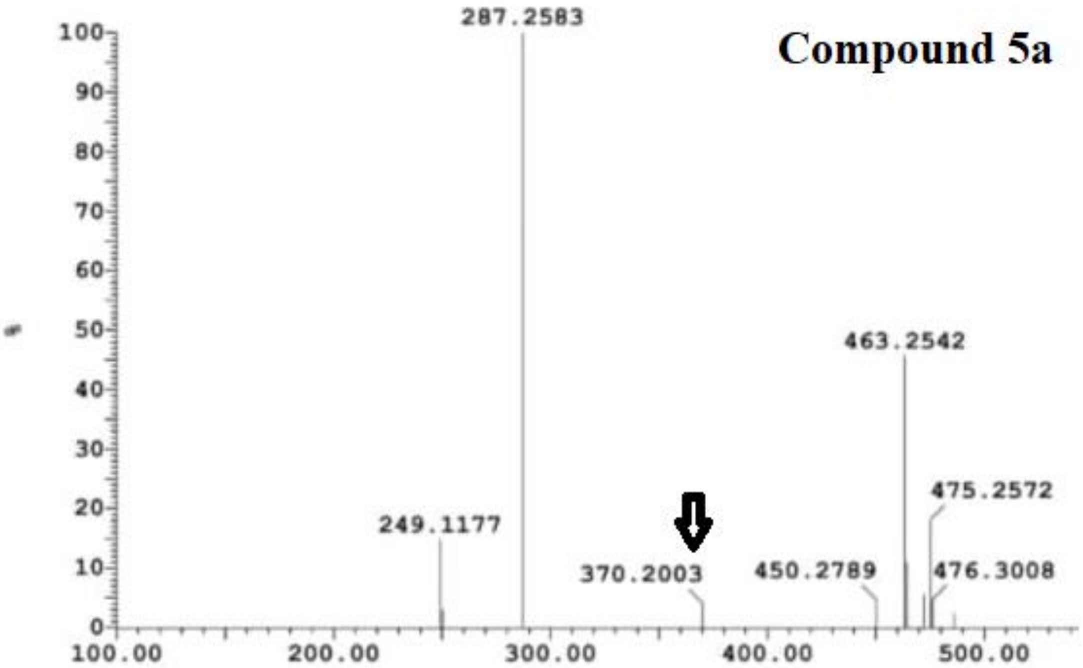

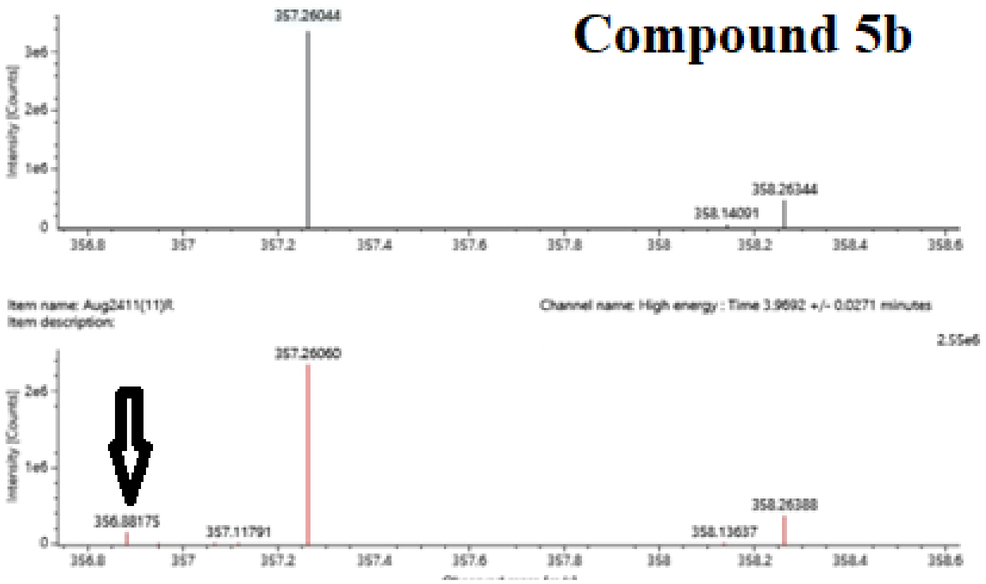

Center for DRUG DISCOVERY RESEARCH and DEVELOPMENT
Openlynx Report -

Sample: 2097 Vial:1:B,3
File:10 Date:27-Aug-2024
Description:10

Printed: Tue Aug 27 11:55:24 2024

Peak ID	Time
24	9.99

24: (Time: 9.99)

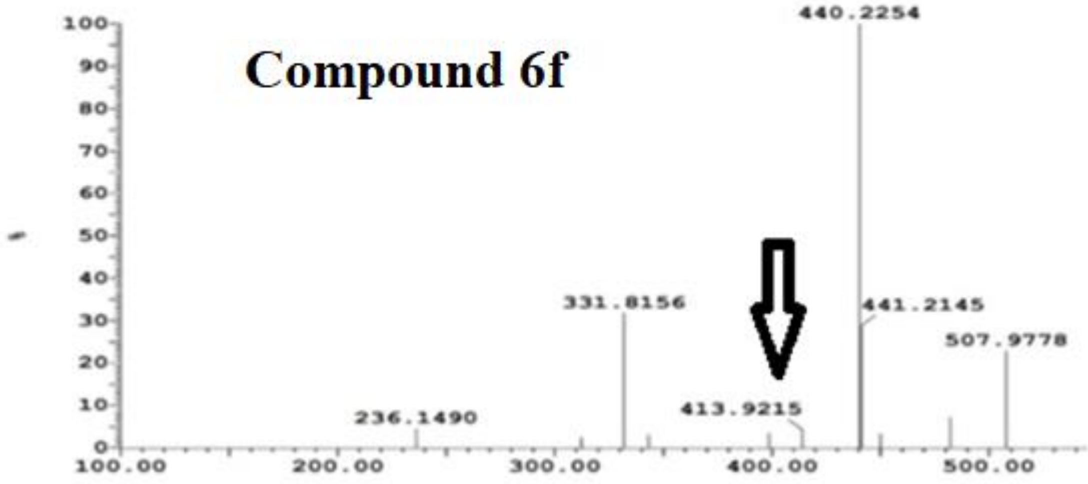

Compound 2a

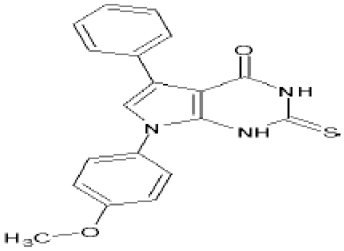
O
NH
NH
S
N
H_3C—O

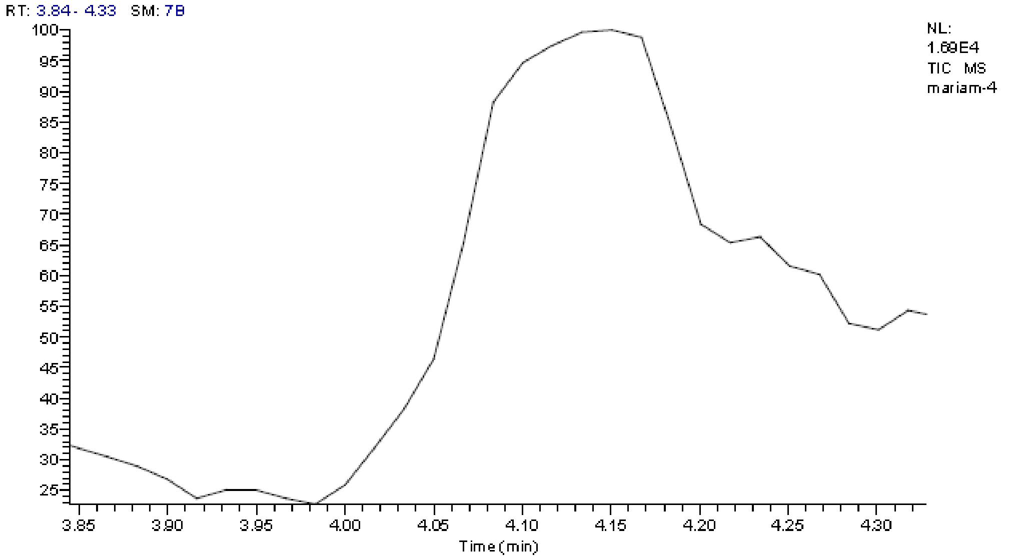
RT: 3.84- 4.33 SM: 7B
NL:
1.69E4
TIC MS
mariam-4
Time (min)

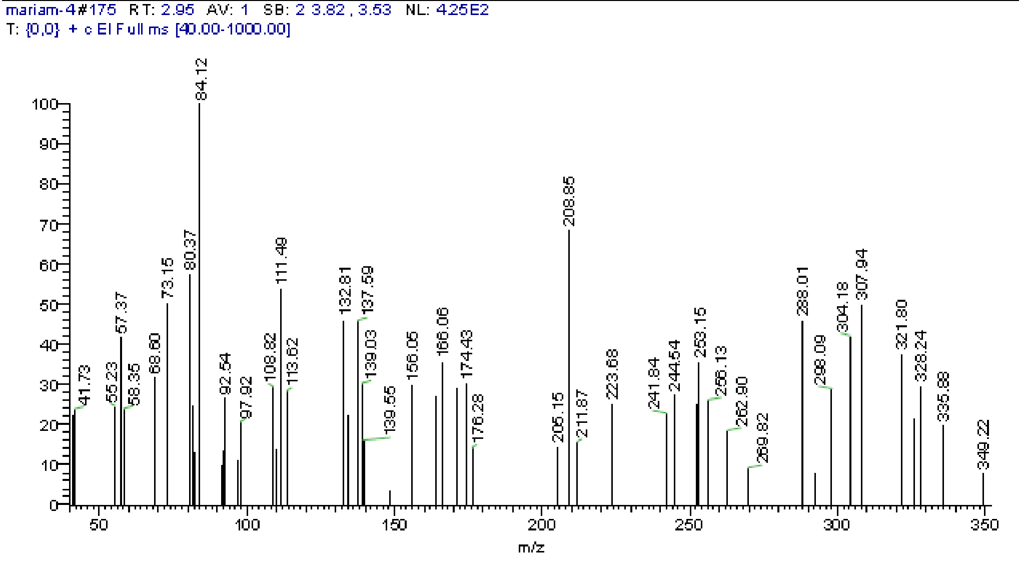
mariam-4#175 RT: 2.95 AV: 1 SB: 2 3.82 , 3.53 NL: 4.25E2
T: {0,0} + c EI Full ms [40.00-1000.00]
84.12
208.85
80.37
73.15
111.49
132.81
137.59
307.94
288.01
304.18
321.80
57.37
68.60
41.73
55.23
58.35
92.54
97.92
108.82
113.62
139.03
139.55
156.05
166.06
174.43
176.28
205.15
211.87
223.68
241.84
244.54
253.15
256.13
262.90
269.82
298.09
328.24
335.88
349.22
m/z

Compound 2b

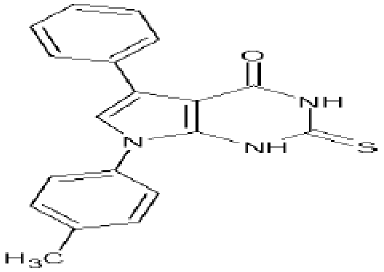
O
NH
NH
S
N
H3C

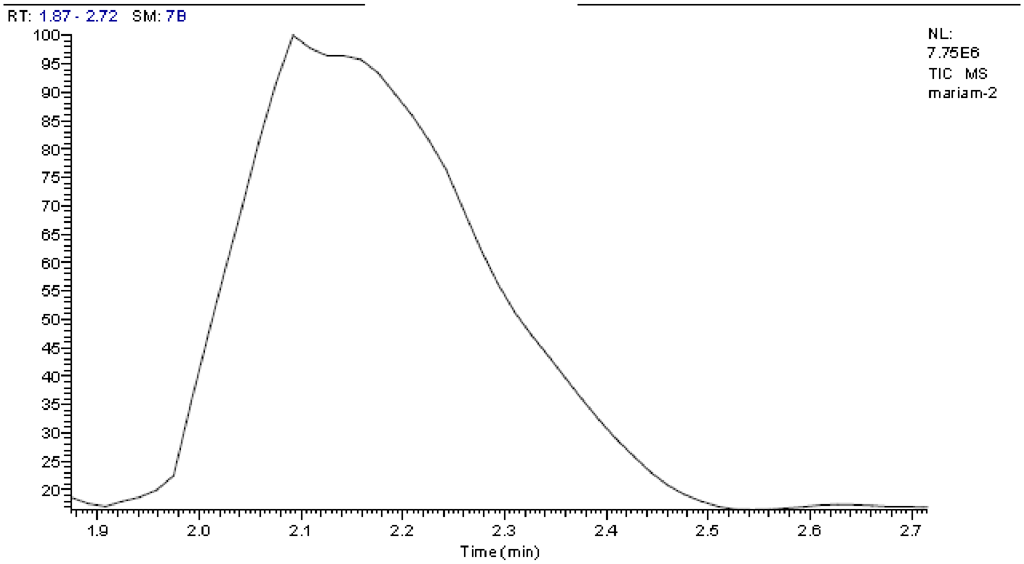
RT: 1.87 - 2.72 SM: 7B
NL:
7.75E6
TIC MS
mariam-2
Time (min)

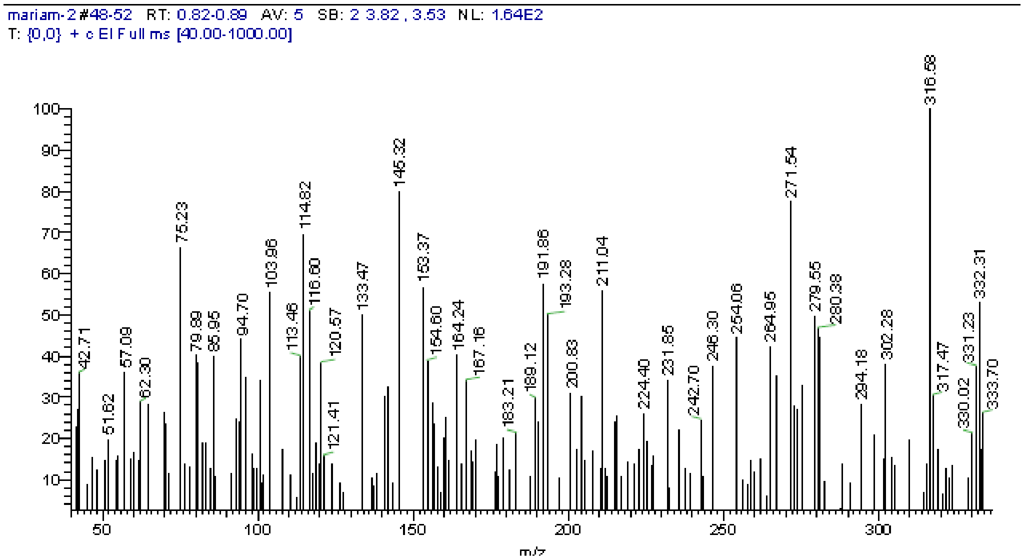
mariam-2 #48-52 RT: 0.82-0.89 AV: 5 SB: 2 3.82 , 3.53 NL: 1.64E2
T: {0,0} + c EI Full ms [40.00-1000.00]
m/z

Compound 2c

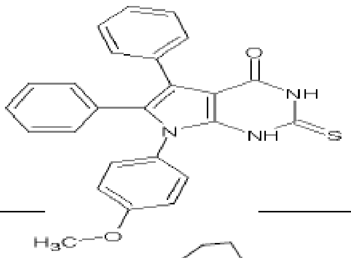
O
NH
NH
S
N
H3C—O

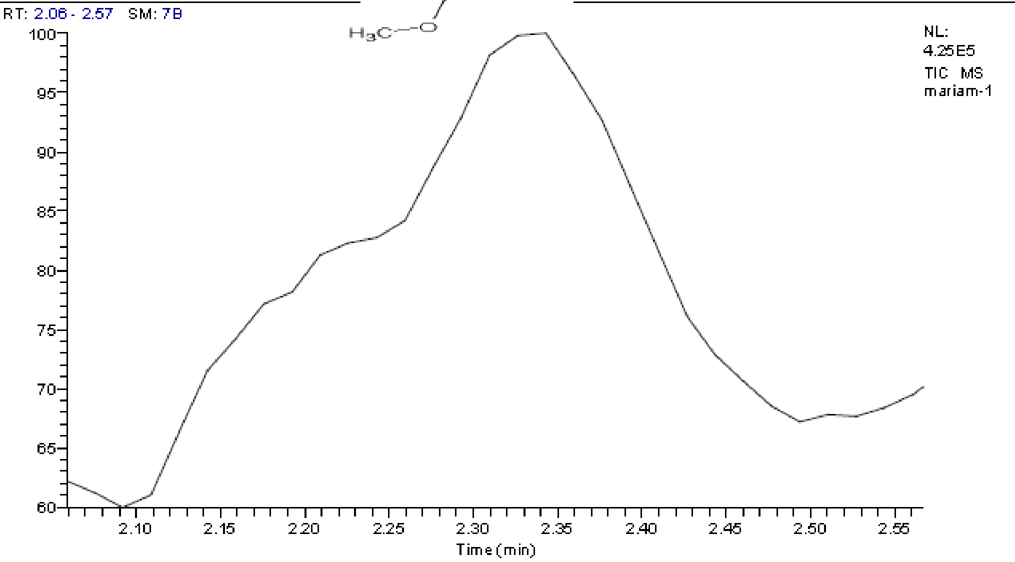
RT: 2.06 - 2.57 SM: 7B
NL:
4.25E5
TIC MS
mariam-1
100
95
90
85
80
75
70
65
60
2.10
2.15
2.20
2.25
2.30
2.35
2.40
2.45
2.50
2.55
Time (min)

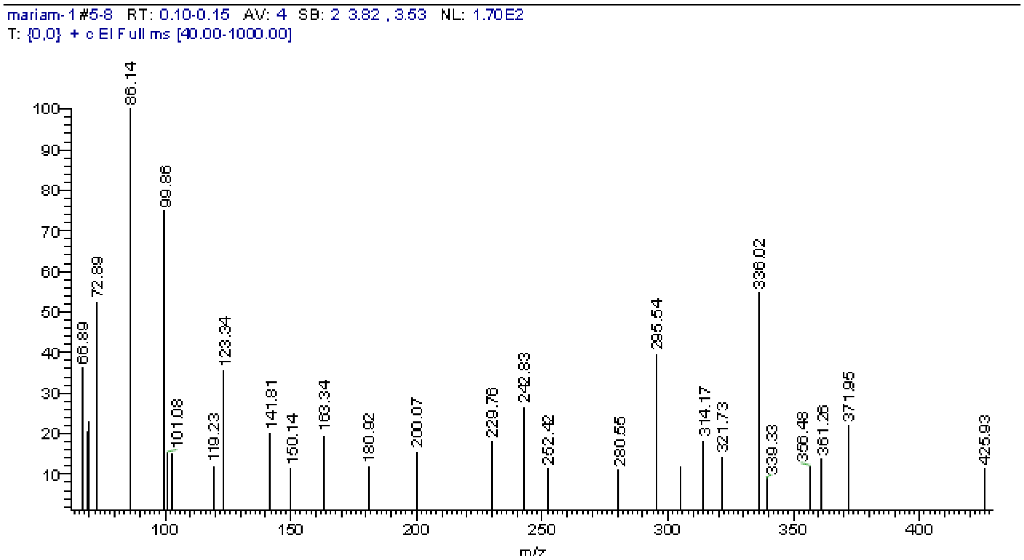
mariam-1 #5-8 RT: 0.10-0.15 AV: 4 SB: 2 3.82 , 3.53 NL: 1.70E2
T: {0,0} + c EI Full ms [40.00-1000.00]
86.14
99.86
72.89
66.89
101.08
119.23
123.34
141.81
150.14
163.34
180.92
200.07
229.76
242.83
252.42
280.55
295.54
314.17
321.73
336.02
339.33
356.48
361.26
371.95
425.93
100
90
80
70
60
50
40
30
20
10
100
150
200
250
300
350
400
m/z

Compound 3a

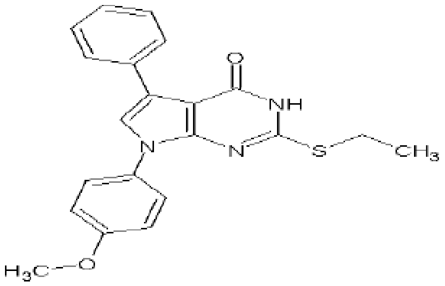

O
NH
N
N
S
CH3
H3C
O

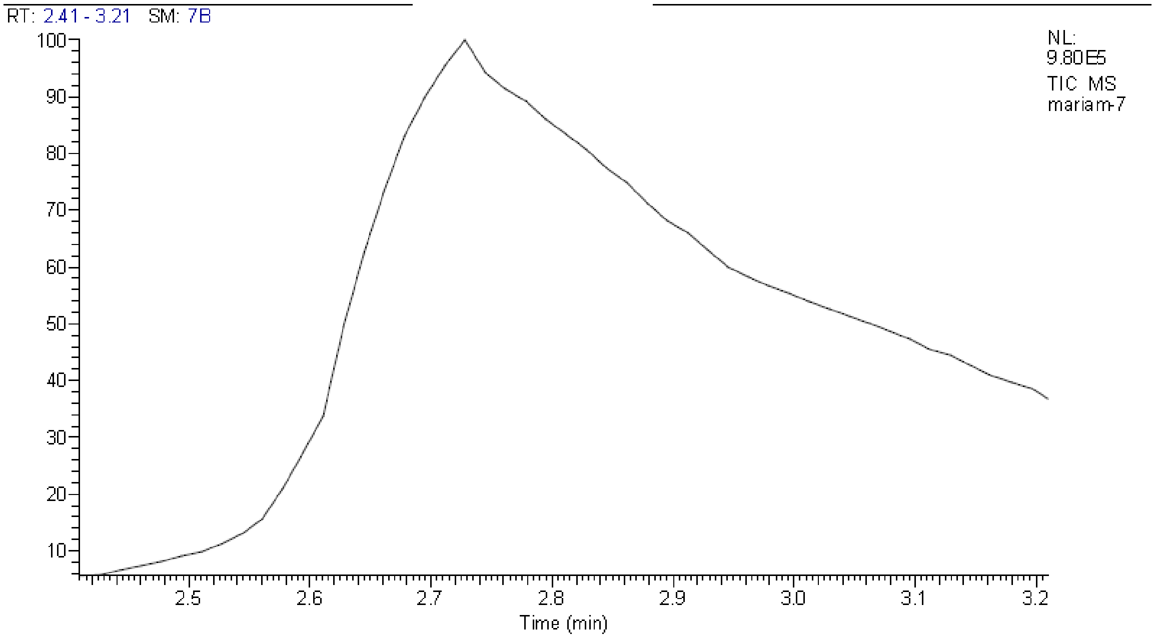

RT: 2.41 - 3.21 SM: 7B
NL:
9.80E5
TIC MS
mariam-7
Time (min)

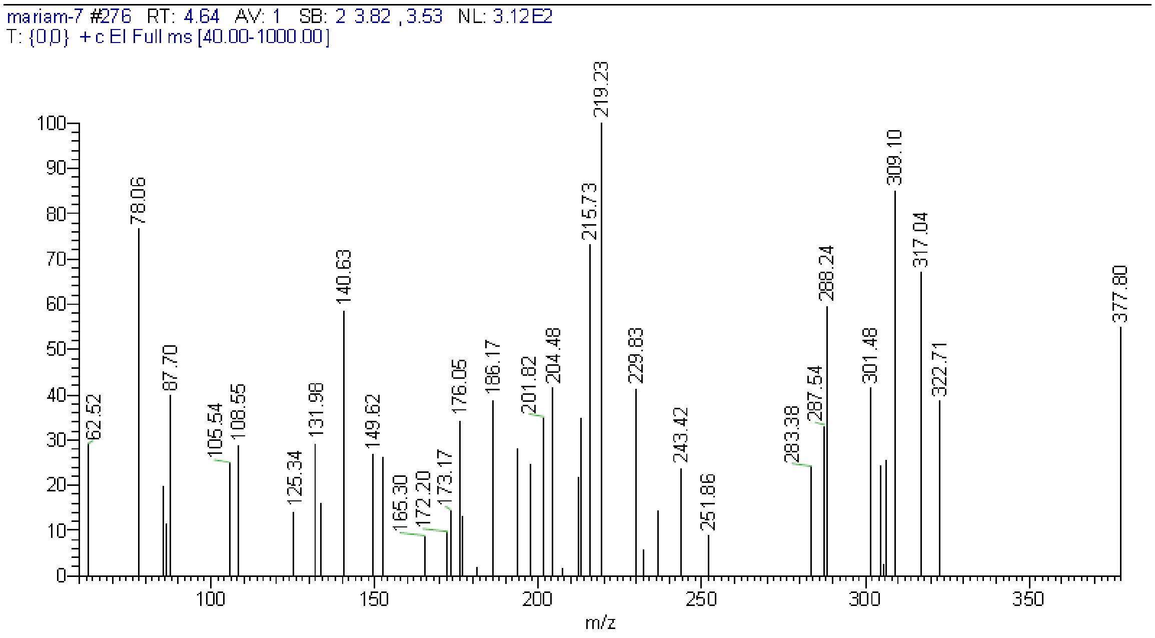

mariam-7 #276 RT: 4.64 AV: 1 SB: 2 3.82 , 3.53 NL: 3.12E2
T: {0,0} + c EI Full ms [40.00-1000.00]
219.23
309.10
78.06
215.73
317.04
288.24
140.63
377.80
204.48
301.48
229.83
322.71
87.70
186.17
201.82
176.05
62.52
105.54
108.55
131.98
149.62
283.38
287.54
243.42
125.34
165.30
172.20
173.17
251.86
m/z

Compound 3c

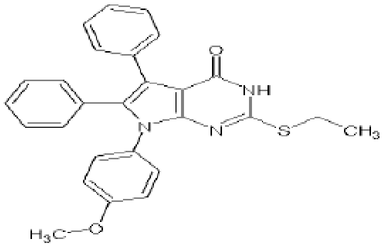

O
NH
N
N
S
CH_3
H_3C
O

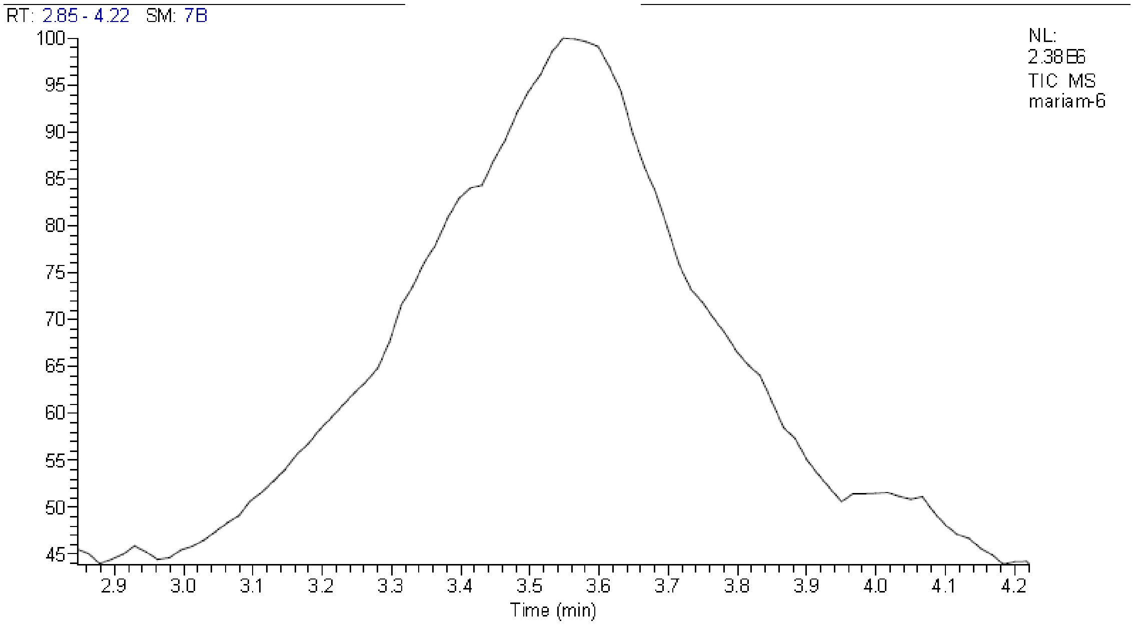

RT: 2.85 - 4.22 SM: 7B
NL:
2.38E6
TIC MS
mariam-6
Time (min)

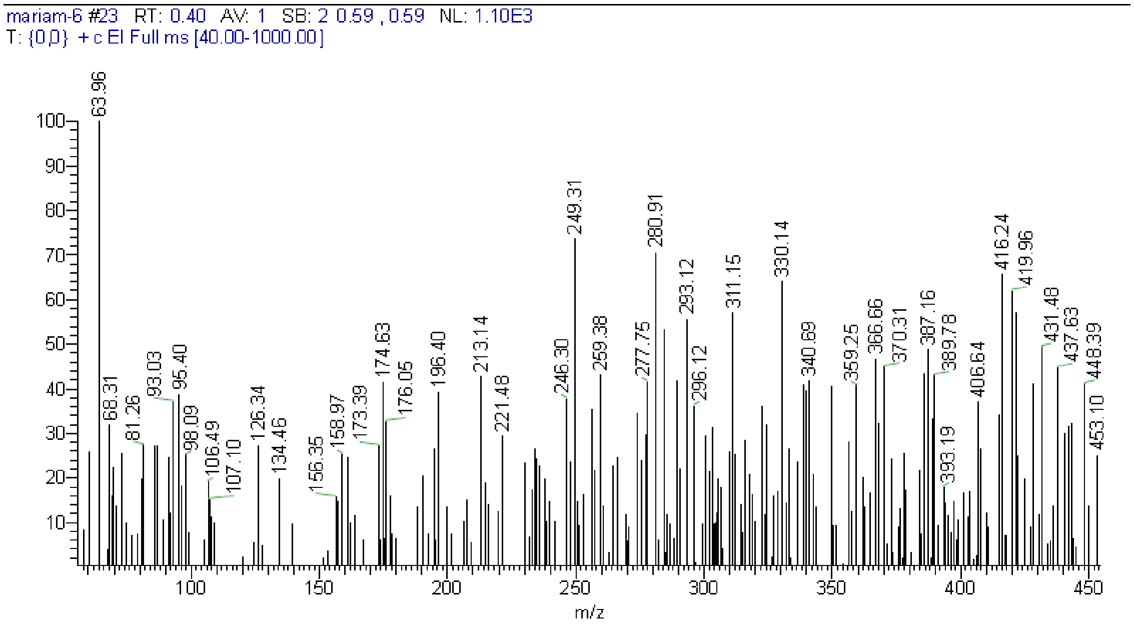

mariam-6 #23 RT: 0.40 AV: 1 SB: 2 0.59 , 0.59 NL: 1.10E3
T: {0,0} + c EI Full ms [40.00-1000.00]
63.96
68.31
81.26
93.03
95.40
98.09
106.49
107.10
126.34
134.46
156.35
158.97
173.39
174.63
176.05
196.40
213.14
221.48
246.30
249.31
259.38
277.75
280.91
293.12
296.12
311.15
330.14
340.69
359.25
366.66
370.31
387.16
389.78
393.19
406.64
416.24
419.96
431.48
437.63
448.39
453.10
m/z

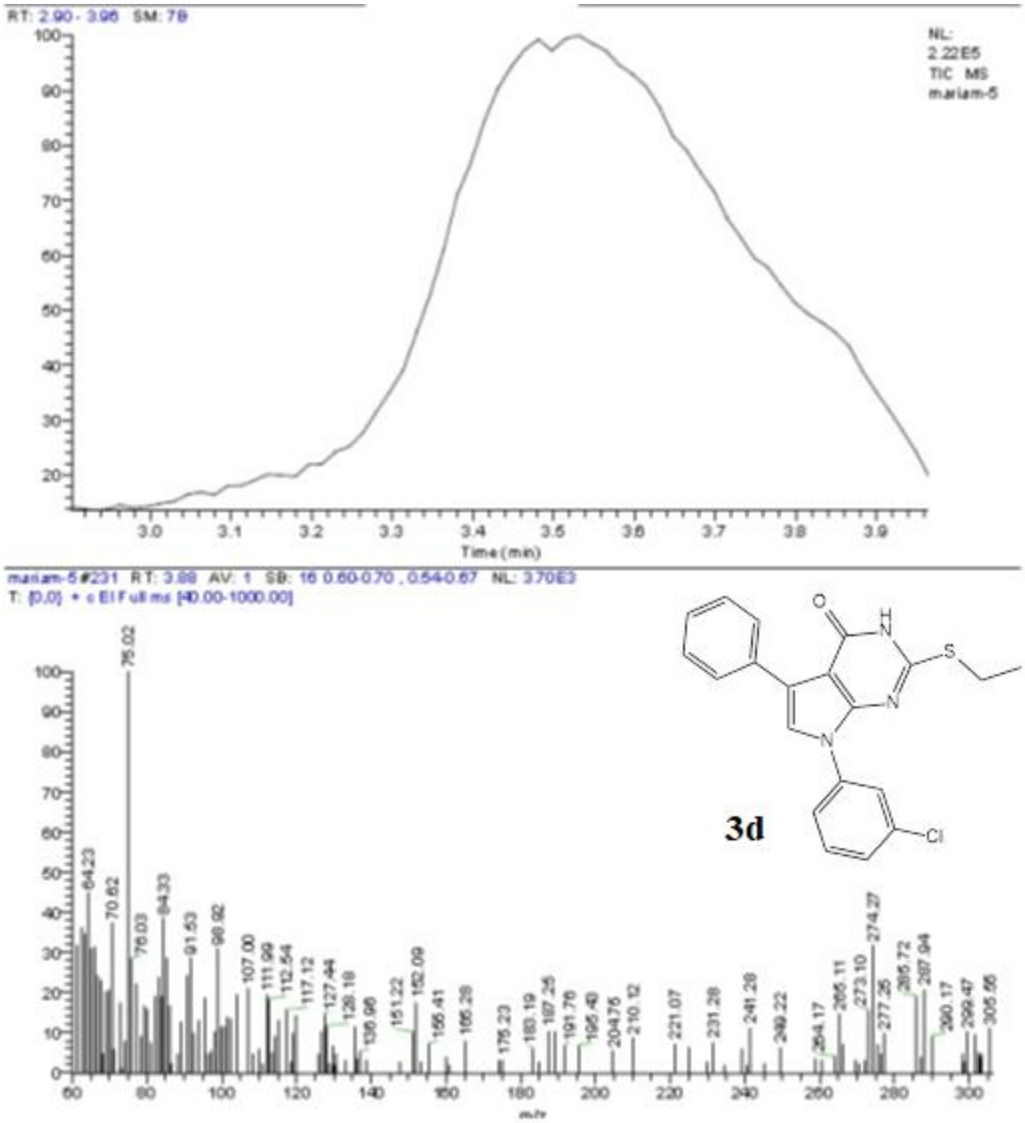
RT: 2.90 - 3.96 SM: 7B
NL: 2.22E5 TIC MS mariam-5
Time (min)
mariam-5#231 RT: 3.88 AV: 1 SB: 16 0.60-0.70 , 0.54-0.67 NL: 3.70E3
T: {0,0} + c EI Full ms [40.00-1000.00]
m/z
O
H
N
S
N
N
Cl
3d

Compound 4a

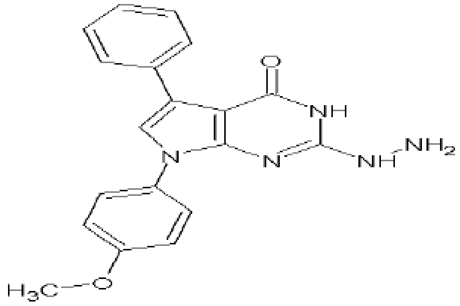

O
NH
NH
NH2
N
N
H3C
O

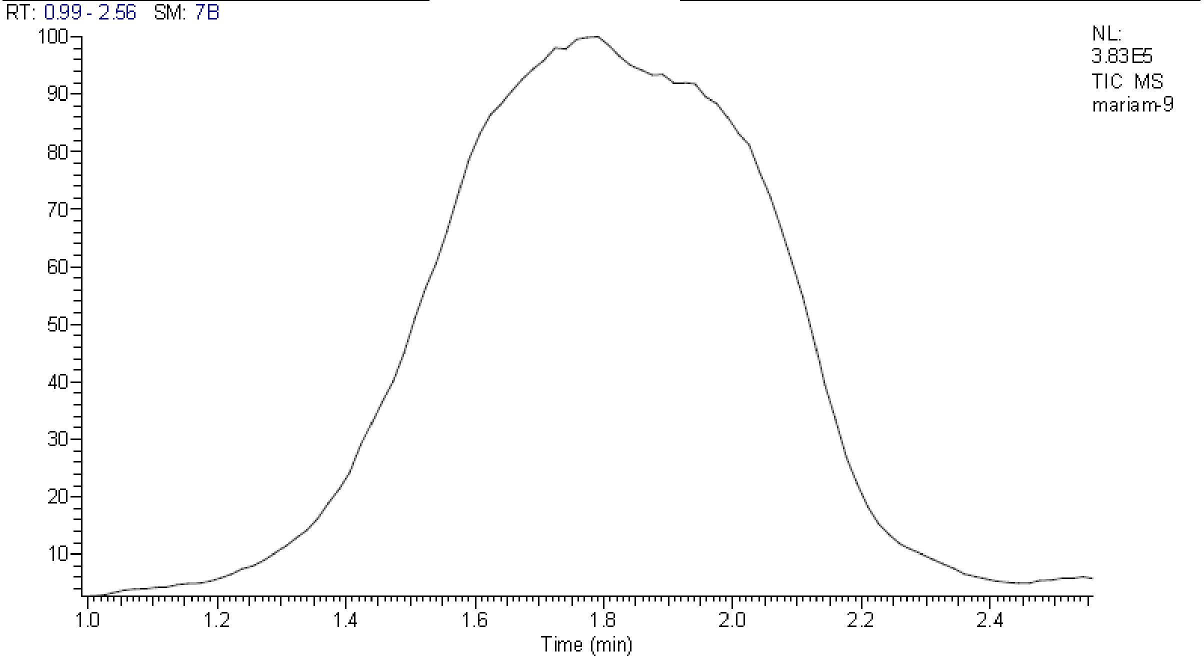

RT: 0.99 - 2.56 SM: 7B
NL:
3.83E5
TIC MS
mariam-9
Time (min)

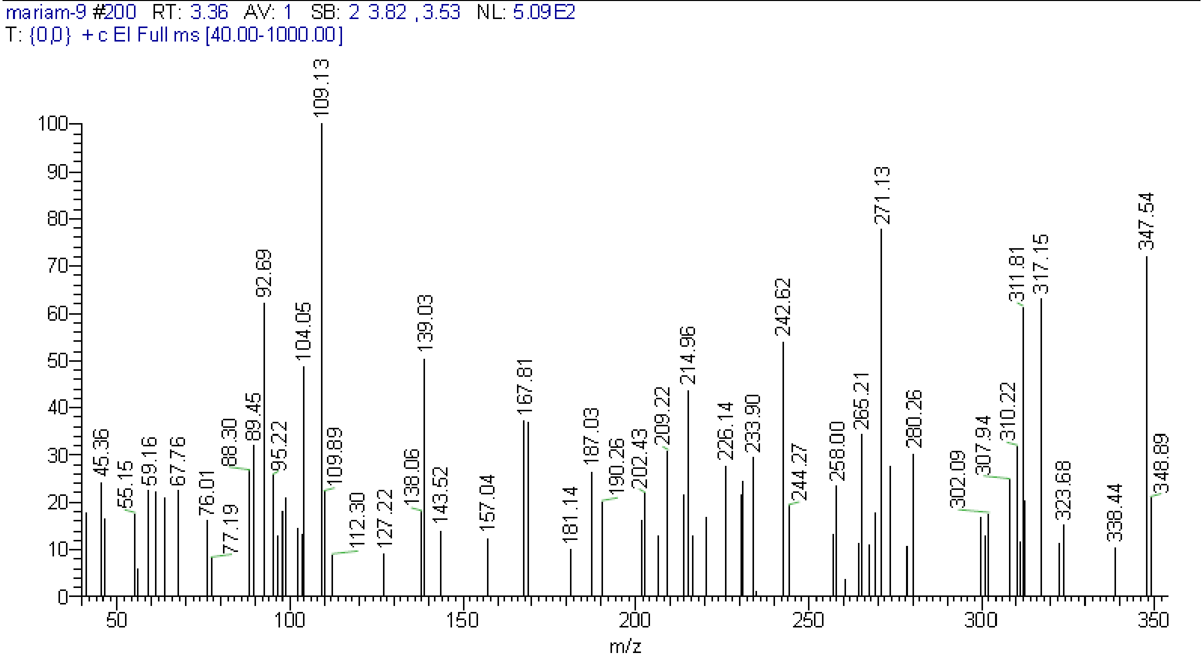

mariam-9 #200 RT: 3.36 AV: 1 SB: 2 3.82 , 3.53 NL: 5.09E2
T: {0,0} + c EI Full ms [40.00-1000.00]
109.13
271.13
347.54
317.15
311.81
92.69
242.62
104.05
139.03
214.96
167.81
187.03
209.22
226.14
233.90
265.21
280.26
310.22
307.94
302.09
258.00
244.27
202.43
190.26
181.14
157.04
143.52
138.06
127.22
112.30
109.89
95.22
89.45
88.30
77.19
76.01
67.76
59.16
55.15
45.36
323.68
338.44
348.89
m/z

Compound 5a

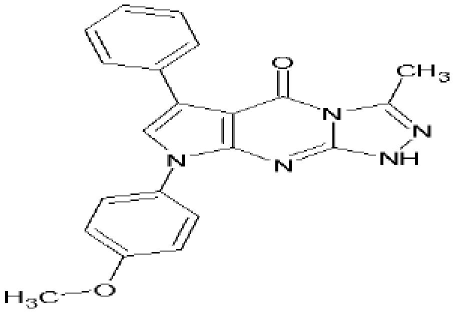

O
CH3
N
N
NH
N
N
H3C—O

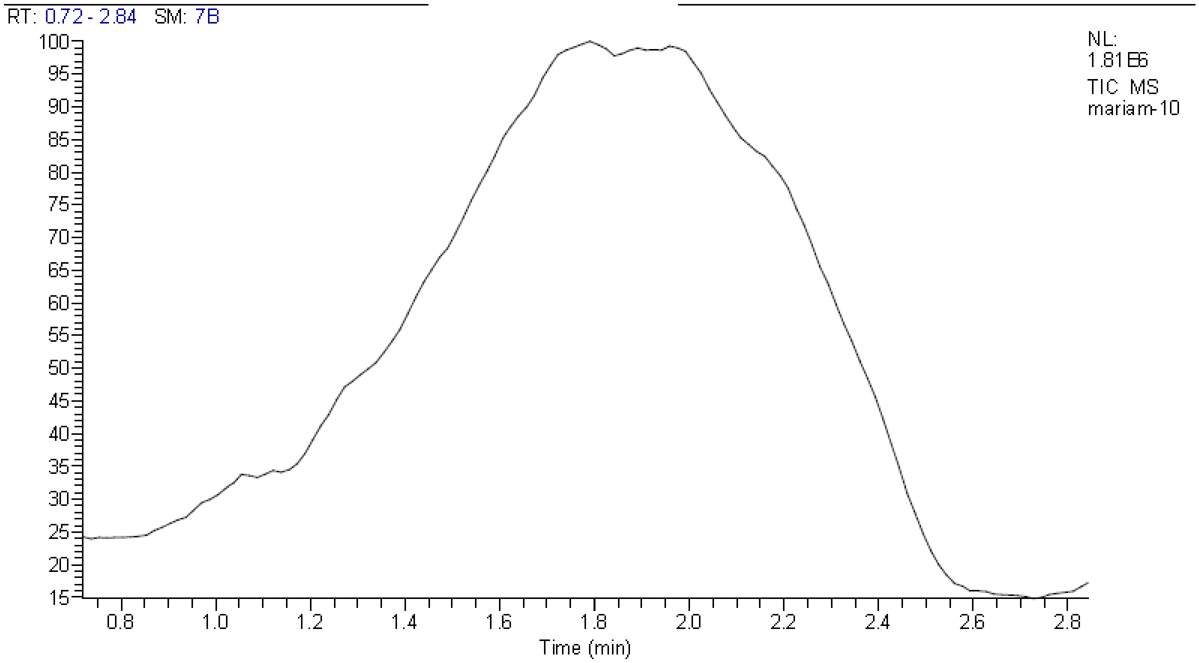

RT: 0.72 - 2.84 SM: 7B
NL: 1.81E6
TIC MS
mariam-10
Time (min)

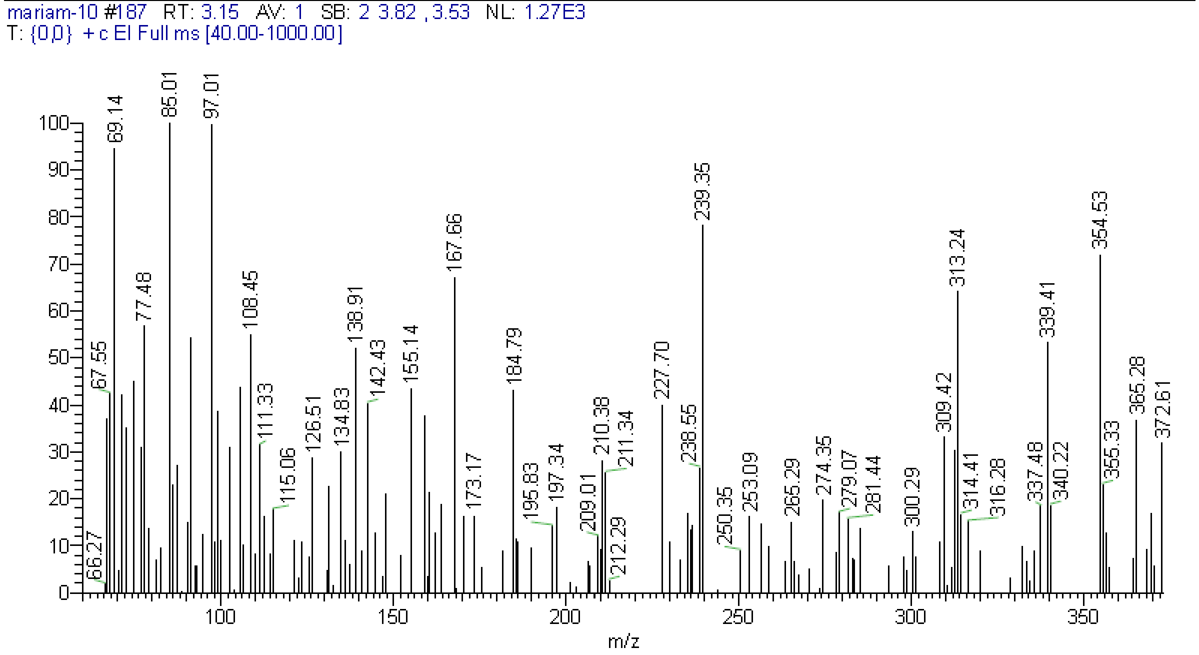

mariam-10 #187 RT: 3.15 AV: 1 SB: 2 3.82 , 3.53 NL: 1.27E3
T: {0,0} + c EI Full ms [40.00-1000.00]
69.14
85.01
97.01
67.55
77.48
108.45
111.33
115.06
126.51
134.83
138.91
142.43
155.14
167.66
173.17
184.79
195.83
197.34
209.01
210.38
211.34
212.29
227.70
238.55
239.35
250.35
253.09
265.29
274.35
279.07
281.44
300.29
309.42
313.24
314.41
316.28
337.48
339.41
340.22
354.53
355.33
365.28
372.61
66.27
m/z

Compound 5b

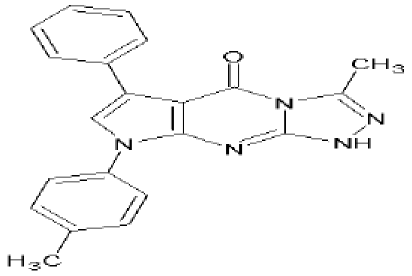
O
CH_3
N
N
NH
N
N
H_3C

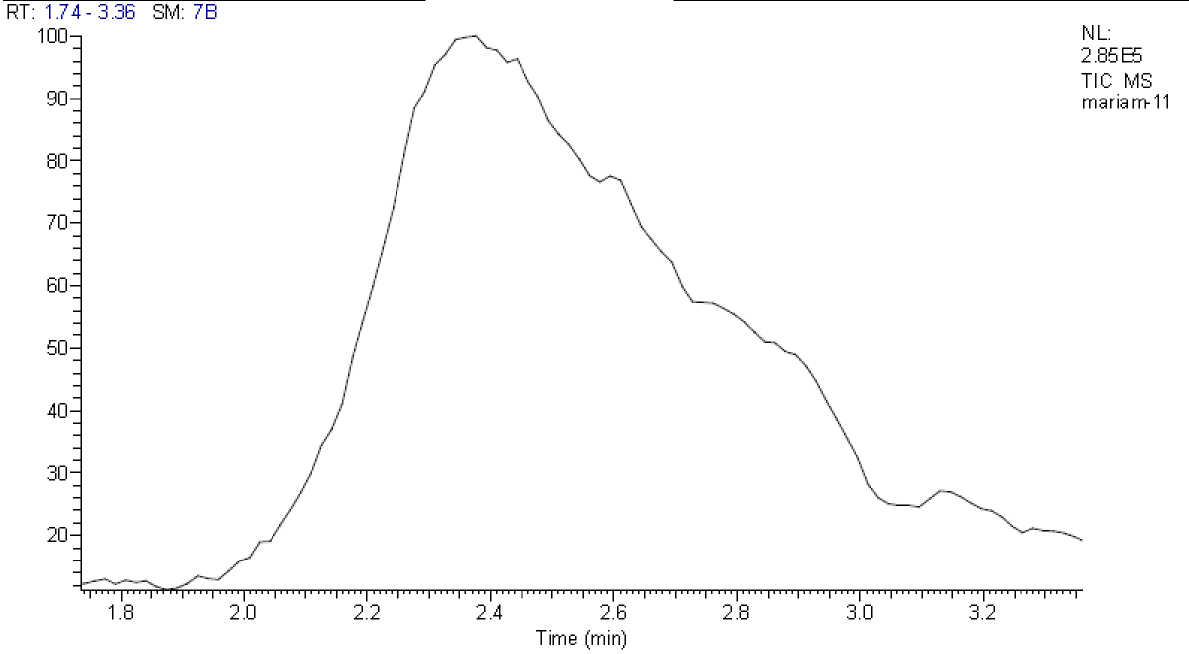
RT: 1.74 - 3.36 SM: 7B
NL:
2.85E5
TIC MS
mariam-11
Time (min)

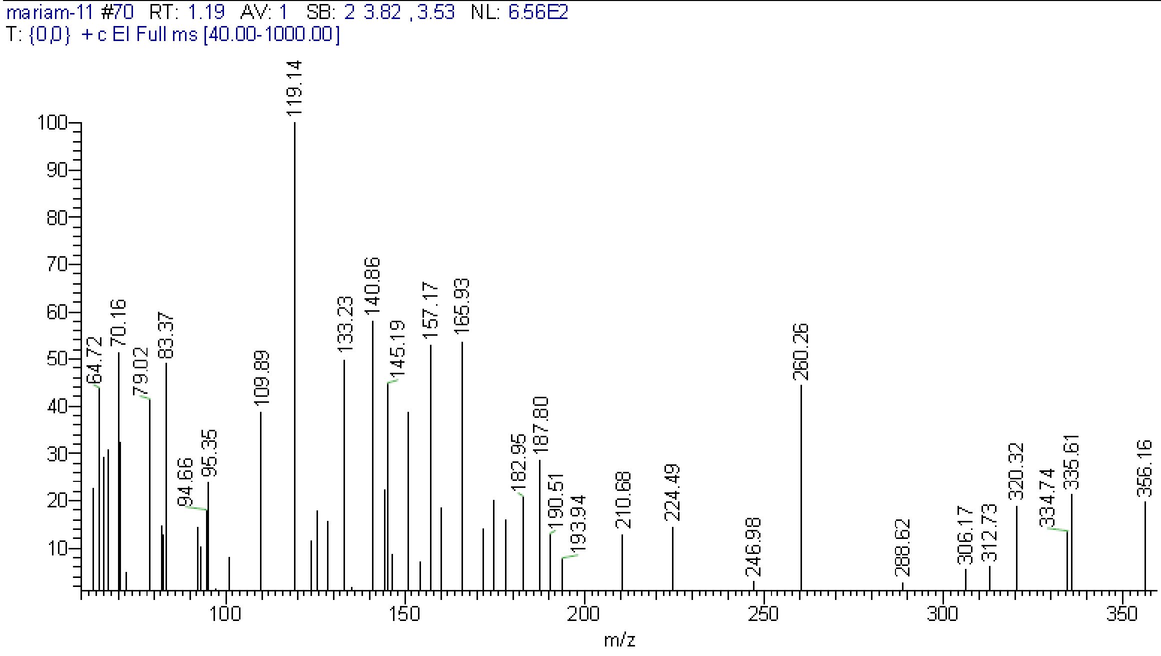
mariam-11 #70 RT: 1.19 AV: 1 SB: 2 3.82 , 3.53 NL: 6.56E2
T: {0,0} + c EI Full ms [40.00-1000.00]
119.14
140.86
133.23
165.93
157.17
145.19
70.16
83.37
64.72
79.02
109.89
260.26
95.35
94.66
187.80
182.95
190.51
193.94
210.68
224.49
246.98
288.62
306.17
312.73
320.32
334.74
335.61
356.16
m/z

Compound 5c

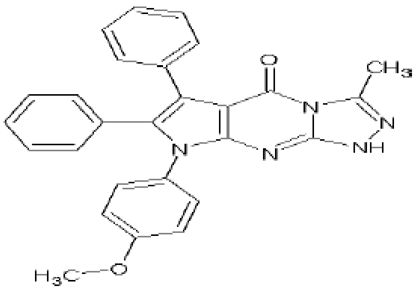
CH3
N
N
NH
N
N
O
H3C

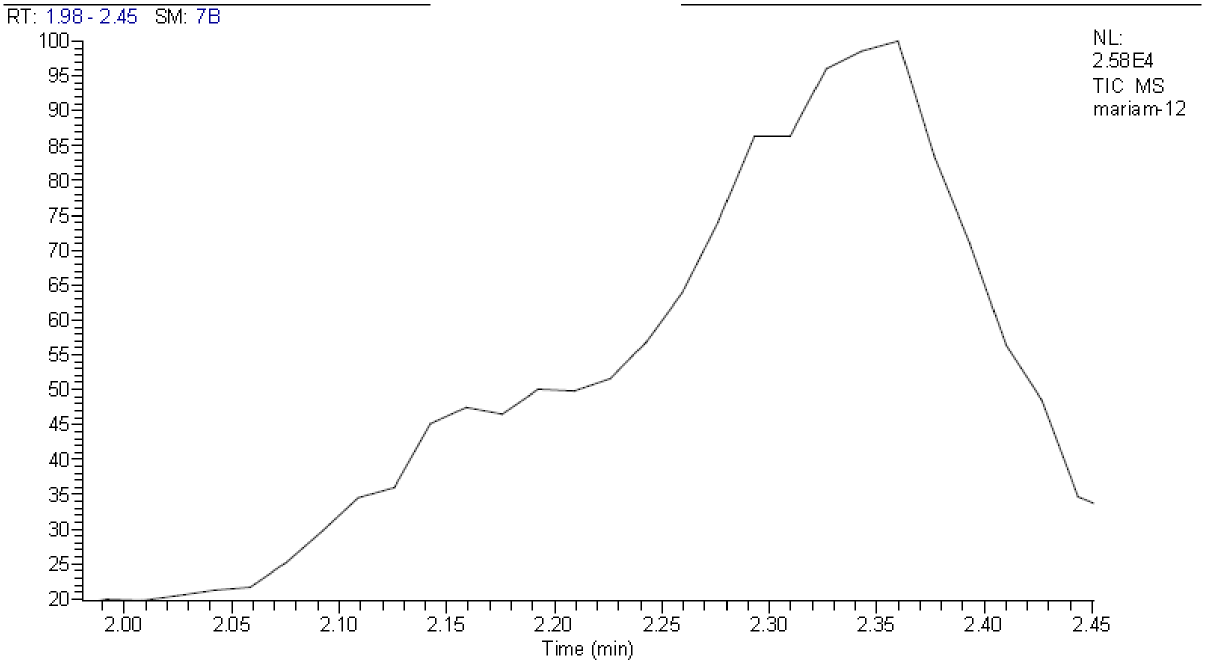
RT: 1.98 - 2.45 SM: 7B
NL:
2.58E4
TIC MS
mariam-12
Time (min)

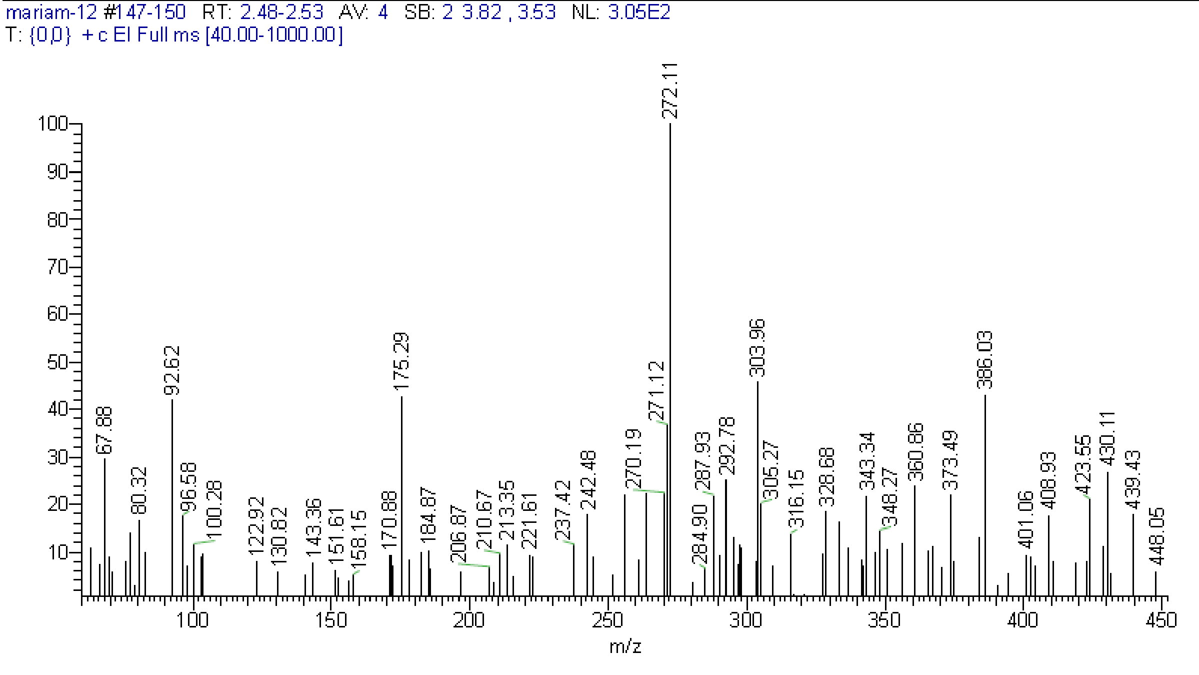
mariam-12 #147-150 RT: 2.48-2.53 AV: 4 SB: 2 3.82 , 3.53 NL: 3.05E2
T: {0,0} + c EI Full ms [40.00-1000.00]
272.11
303.96
386.03
175.29
92.62
271.12
67.88
430.11
m/z

Compound 6b

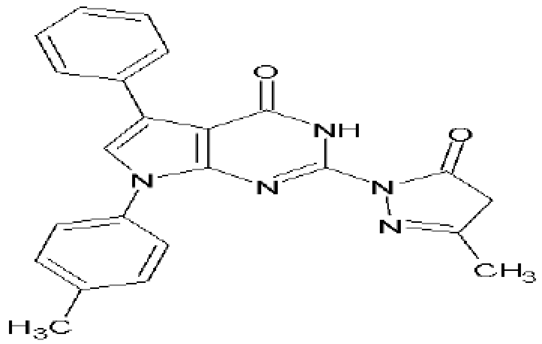

O
NH
O
N
N
N
N
N
CH3
H3C

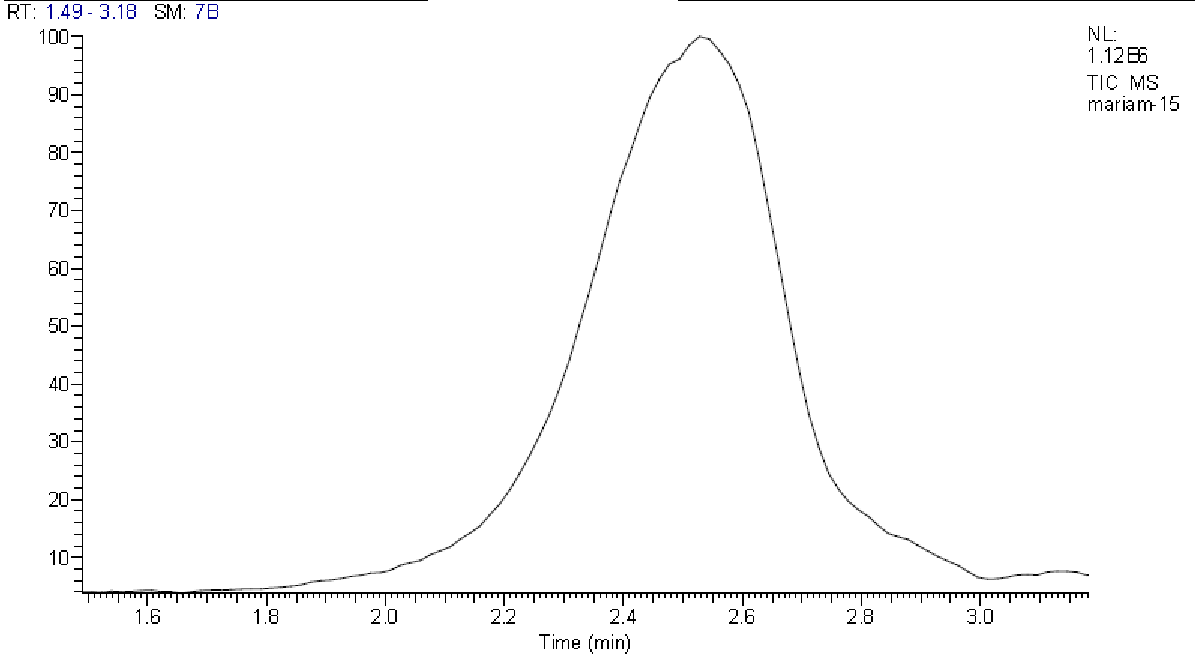

RT: 1.49 - 3.18 SM: 7B
NL: 1.12E6
TIC MS
mariam-15
Time (min)

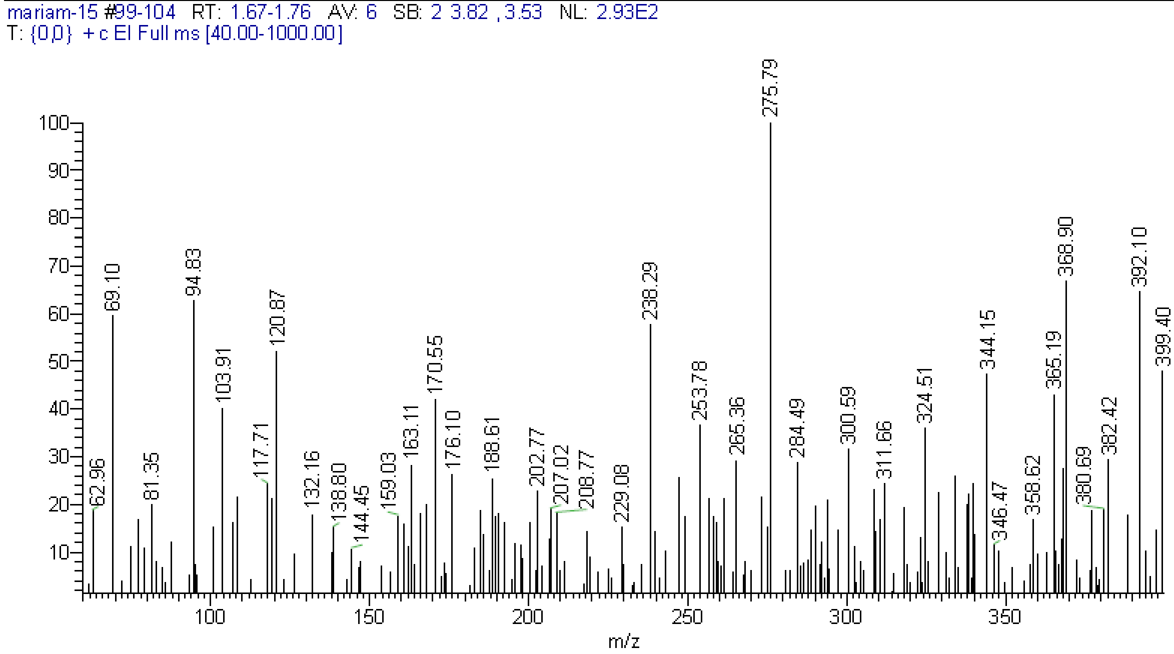

mariam-15 #99-104 RT: 1.67-1.76 AV: 6 SB: 2 3.82 , 3.53 NL: 2.93E2
T: {0,0} + c EI Full ms [40.00-1000.00]
275.79
392.10
368.90
399.40
238.29
344.15
94.83
69.10
120.87
170.55
103.91
365.19
253.78
324.51
300.59
265.36
284.49
311.66
m/z

Compound 7a

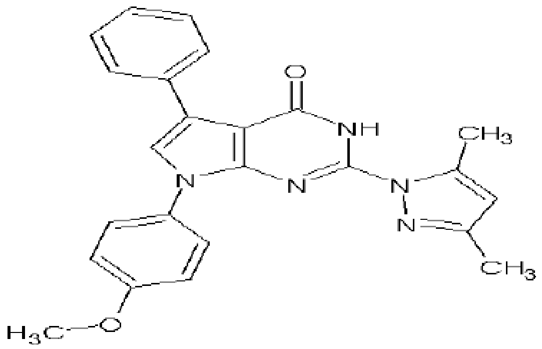
O
NH
CH3
N
N
N
N
N
CH3
H3C
O

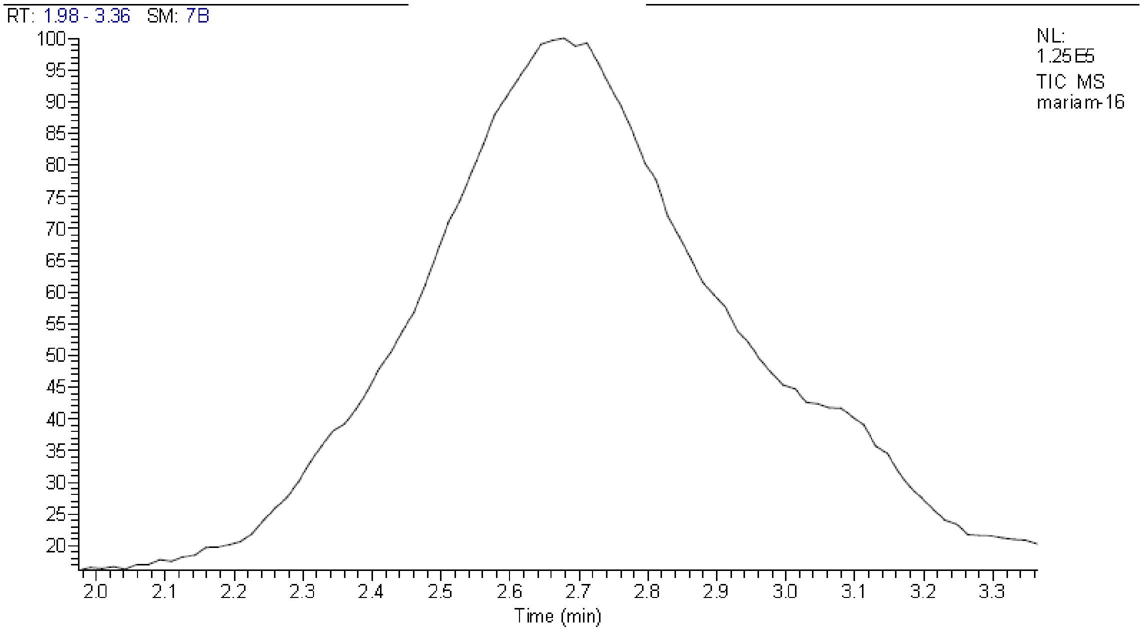
RT: 1.98 - 3.36 SM: 7B
NL: 1.25E5
TIC MS
mariam-16
Time (min)

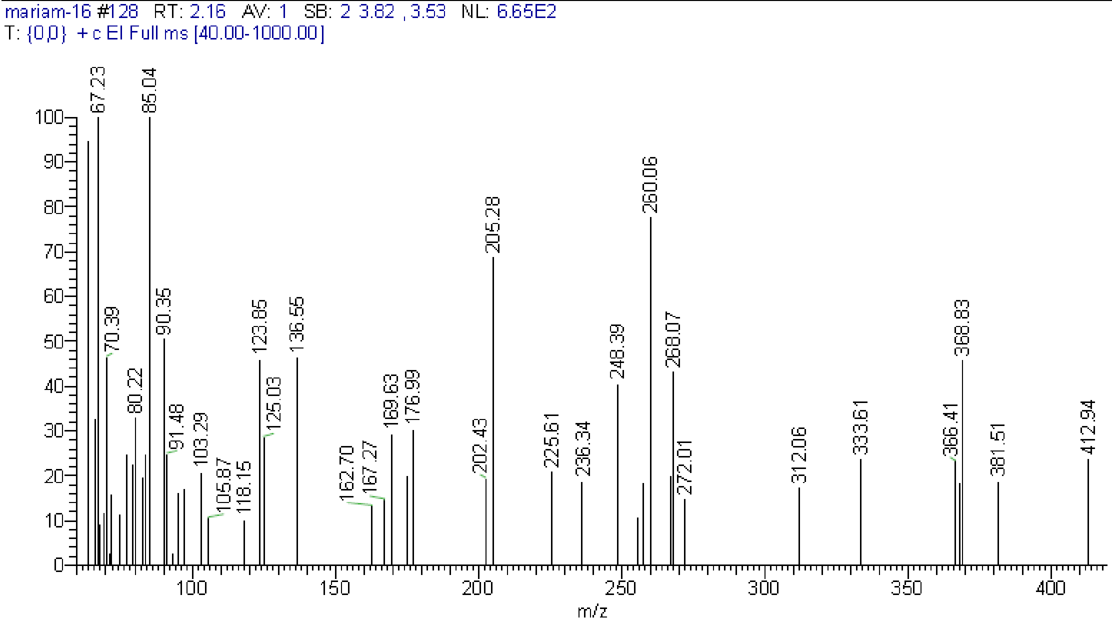
mariam-16 #128 RT: 2.16 AV: 1 SB: 2 3.82 , 3.53 NL: 6.65E2
T: {0,0} + c EI Full ms [40.00-1000.00]
67.23
85.04
70.39
80.22
90.35
91.48
103.29
105.87
118.15
123.85
125.03
136.55
162.70
167.27
169.63
176.99
202.43
205.28
225.61
236.34
248.39
260.06
268.07
272.01
312.06
333.61
366.41
368.83
381.51
412.94
m/z

Compound 7c

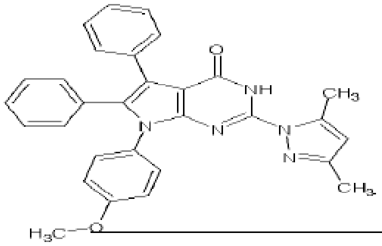

O
NH
CH3
N
N
N
N
CH3
H3C—O

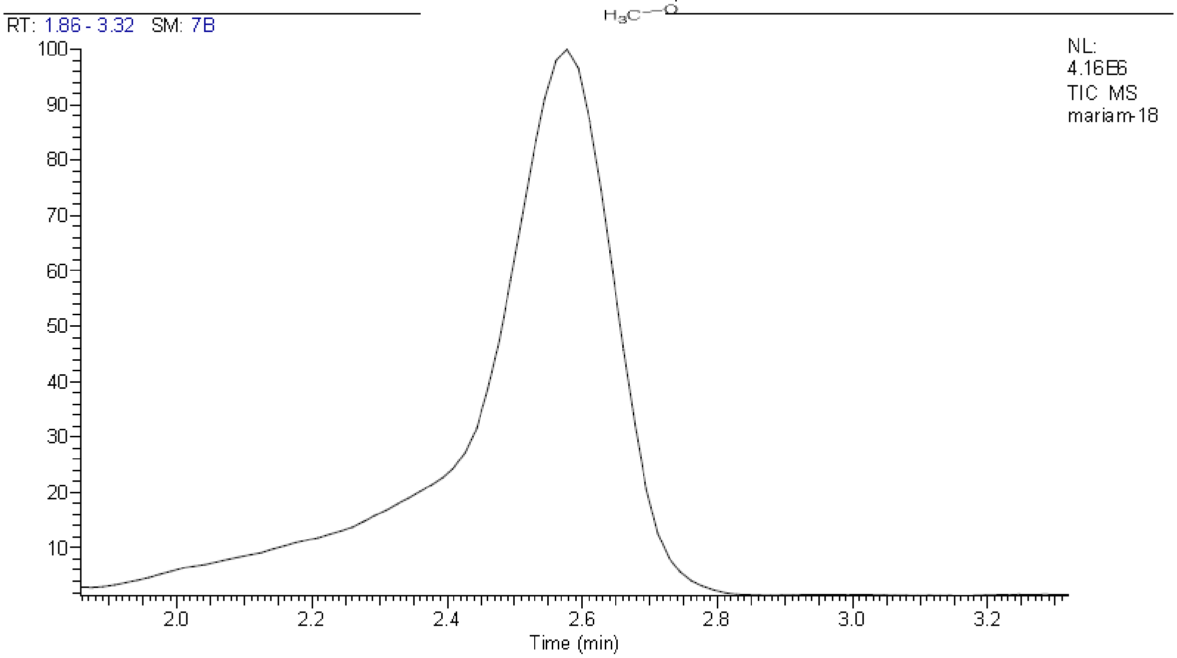

RT: 1.86 - 3.32 SM: 7B
NL: 4.16E6 TIC MS mariam-18
Time (min)

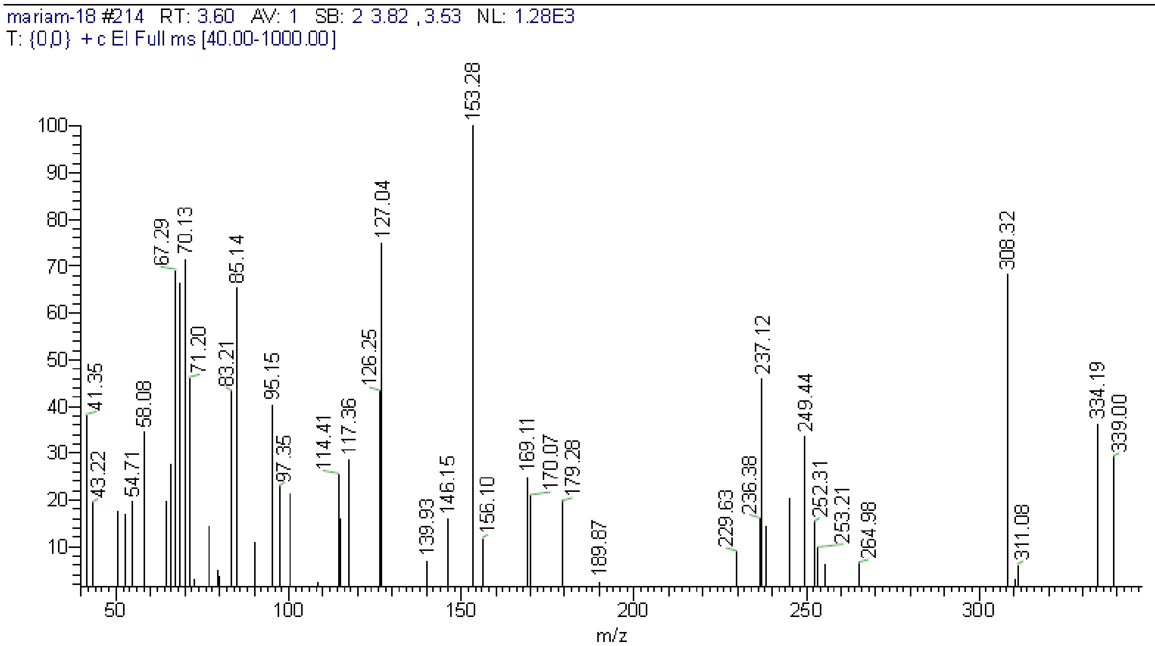

mariam-18 #214 RT: 3.60 AV: 1 SB: 2 3.82 , 3.53 NL: 1.28E3
T: {0,0} + c EI Full ms [40.00-1000.00]
153.28
127.04
308.32
70.13
67.29
85.14
237.12
71.20
83.21
95.15
126.25
41.35
334.19
339.00
249.44
58.08
117.36
114.41
169.11
170.07
179.28
43.22
54.71
97.35
146.15
252.31
236.38
156.10
253.21
229.63
264.98
139.93
311.08
189.87
m/z

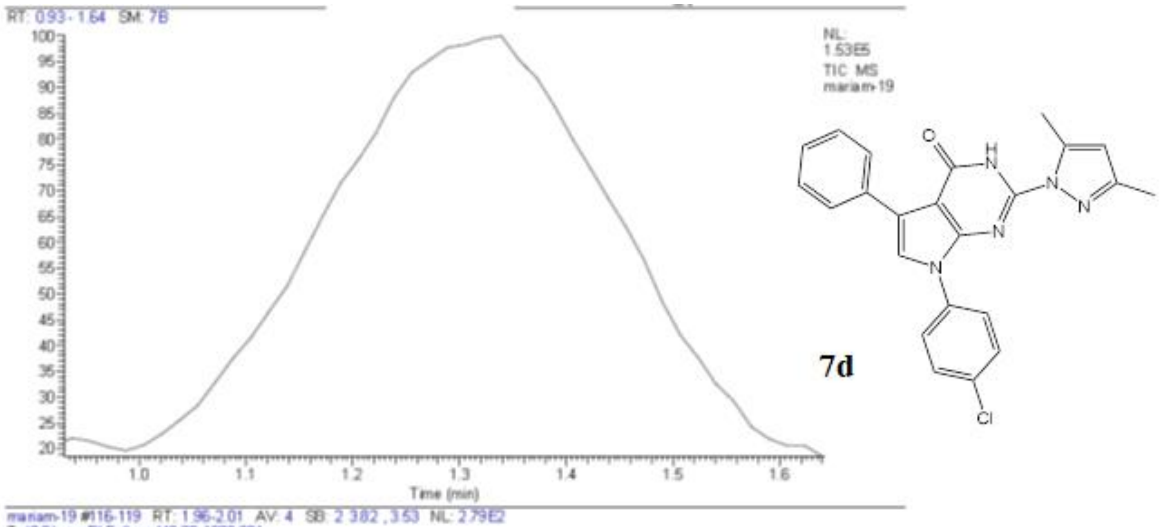
RT: 0.93 - 1.64 SM: 7B
NL:
1.53E5
TIC MS
mariam-19
Time (min)
O
H
N
N
N
N
N
Cl

7d

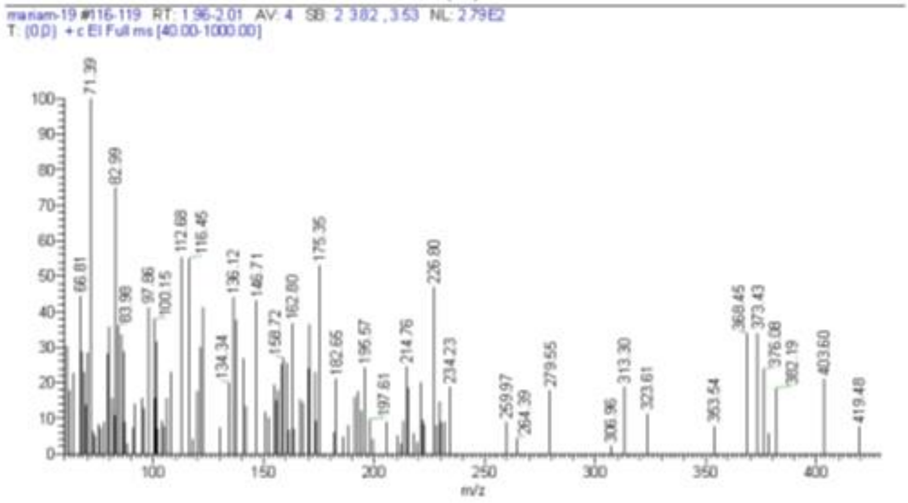
mariam-19 #116-119 RT: 1.96-2.01 AV: 4 SB: 2 3.82 , 3.53 NL: 2.79E2
T: {0,0} + c EI Full ms [40.00-1000.00]
71.39
82.99
66.81
83.98
97.86
100.15
112.68
116.45
134.34
136.12
146.71
158.72
162.80
175.35
182.65
195.57
197.61
214.76
226.80
234.23
259.97
264.39
279.55
306.96
313.30
323.61
353.54
368.45
373.43
376.08
382.19
403.60
419.48
m/z
